# Neurocognitive Function in HIV Infected Patients on Antiretroviral Therapy

**DOI:** 10.1371/journal.pone.0061949

**Published:** 2013-04-30

**Authors:** Alan Winston, Alejandro Arenas-Pinto, Wolfgang Stöhr, Martin Fisher, Chloe M. Orkin, Kazeem Aderogba, Andrew De Burgh-Thomas, Nigel O'Farrell, Charles JN. Lacey, Clifford Leen, David Dunn, Nicholas I. Paton, for the PIVOT Trial Team

**Affiliations:** 1 Section of Infectious Diseases, Division of Medicine, Imperial College London, St Mary's Hospital Campus, Norfolk Place, London, United Kingdom; 2 Department of HIV and GU Medicine, Imperial College Healthcare NHS Trust, London, United Kingdom; 3 Medical Research Council Clinical Trials Unit, London, United Kingdom; 4 Centre for Sexual Health and HIV Research, University College London, London, United Kingdom; 5 Brighton and Sussex University Hospitals NHS Trust, Brighton, United Kingdom; 6 Barts Health NHS Trust, Ambrose King Centre, Royal London Hospital, London, United Kingdom; 7 East Sussex Healthcare Trust, Avenue House, Eastbourne, United Kingdom; 8 Gloucestershire Care Services, Hope House, Gloucestershire Royal Hospital, Gloucester, United Kingdom; 9 Pasteur Suite, Ealing Hospital, London, United Kingdom; 10 Centre for Immunology and Infection, Hull York Medical School, University of York, York, United Kingdom; 11 Edinburgh Infectious Diseases, Edinburgh University Regional Infectious Diseases Unit, Western General Hospital, Edinburgh, United Kingdom; 12 Edinburgh University, Edinburgh, United Kingdom; 13 National University of Singapore, Singapore; University of Nebraska Medical Center, United States of America

## Abstract

**Objective:**

To describe factors associated with neurocognitive (NC) function in HIV-positive patients on stable combination antiretroviral therapy.

**Design:**

We undertook a cross-sectional analysis assessing NC data obtained at baseline in patients entering the Protease-Inhibitor-Monotherapy-Versus-Ongoing-Triple therapy (PIVOT) trial.

**Main outcome measure:**

NC testing comprised of 5 domains. Raw results were z-transformed using standard and demographically adjusted normative datasets (ND). Global *z*-scores (NPZ-5) were derived from averaging the 5 domains and percentage of subjects with test scores >1 standard deviation (SD) below population means in at least two domains (abnormal Frascati score) calculated. Patient characteristics associated with NC results were assessed using multivariable linear regression.

**Results:**

Of the 587 patients in PIVOT, 557 had full NC results and were included. 77% were male, 68% Caucasian and 28% of Black ethnicity. Mean (SD) baseline and nadir CD4+ lymphocyte counts were 553(217) and 177(117) cells/µL, respectively, and HIV RNA was <50 copies/mL in all. Median (IQR) NPZ-5 score was −0.5 (−1.2/−0) overall, and −0.3 (−0.7/0.1) and −1.4 (−2/−0.8) in subjects of Caucasian and Black ethnicity, respectively. Abnormal Frascati scores using the standard-ND were observed in 51%, 38%, and 81%, respectively, of subjects overall, Caucasian and Black ethnicity (p<0.001), but in 62% and 69% of Caucasian and Black subjects using demographically adjusted-ND (p = 0.20). In the multivariate analysis, only Black ethnicity was associated with poorer NPZ-5 scores (*P*<0.001).

**Conclusions:**

In this large group of HIV-infected subjects with viral load suppression, ethnicity but not HIV-disease factors is closely associated with NC results. The prevalence of abnormal results is highly dependent on control datasets utilised.

**Trial registry:**

ClinicalTrials.gov, NCT01230580

## Introduction

Whilst effective combination antiretroviral therapy (cART) has dramatic effects on the incidence of serious neurological complications secondary to chronic HIV infection, including severe HIV-associated dementia [1], [2], high rates of more subtle cognitive decline in HIV infected subjects are being increasingly described with neurocognitive (NC) impairment rates approaching 50% in some cohorts [3], [4], [5]. Several factors have been implicated with the evolution of NC impairment in the cART era including older age [3], low nadir CD4+ lymphocyte count [6], chronic hepatitis-C-virus (HCV) co-infection [7], [8] and possibly the use of antiretroviral regimens with poor central-nervous-system (CNS) exposure [9].

Given the changing picture of HIV associated CNS disease, a revised nomenclature system often known as the Frascati criteria, has been proposed classifying subjects with abnormal NC testing results into three categories based on patients symptoms measured via the activities of daily living scale [10]. Subjects with abnormal NC testing results, who are otherwise asymptomatic are classified as HIV-associated asymptomatic NC impairment (ANI), who are mildly symptomatic are classified as HIV-associated mild NC disorder (MND) and in whom are severely symptomatic classified as HIV-associated dementia (HAD).

Although high rates of NC impairment in HIV infected subjects are reported, the clinical characteristics of these reported cohorts often differ and may include confounding variables which could influence NC function. For instance antiretroviral treatment status [11], levels of plasma HIV viraemia and HIV disease stage [12] and subjects ethnicity [13] vary widely between reports.

In general NC function improves when initiating cART [14], [15], [16] and the majority of HIV infected people attending for care are now on stable ART with full viral load suppression [17], [18]. The aim of this study was to determine the prevalence of NC impairment and the factors associated with NC function in a large contemporary cohort of patients on stable ART.

## Methods

### Subject selection

This cross-sectional analysis included patients whom had agreed to take part in the Protease Inhibitor monotherapy Versus Ongoing Triple-therapy in the long term management of HIV infection (PIVOT) [19] trial. The protocol for this study is available as supporting information; see Protocol S1. Eligibility criteria included documented HIV infection; receiving a stable cART regimen comprising two nucleoside-reverse-transcriptase-inhibitors (NRTIs) with either a non-nucleoside-reverse-transcriptase-inhibitor (NNRTI) or a protease-inhibitor (PI) for at least 24 weeks prior to study entry; a plasma HIV RNA <50 copies/mL at screening and for at least 24 weeks prior to screening; and a CD4+ lymphocyte count >100 cells µL. This paper reports the results of the NC tests done at baseline in patients entering the PIVOT study, prior to randomisation.

The PIVOT study is registered as ClinicalTrials.gov NCT01230580. National Research Ethics Service (NRES) approval for the trial, including the NC assessments, was obtained from the East of England Cambridge South Ethics Committee.

### Neurocognitive measures

NC testing was administered by a designated study nurse or investigator at each study site who had completed standardised training to administer the test procedures. Five domains were assessed as follows; Verbal learning and memory were assessed using *Hopkins Verbal Learning Test-Revised* (HVLT-R) for learning and recall [20], fine motor skills assessed using *Grooved Pegboard* [21], and attention and executive function assessed using *Colour Trails Tests* (CTT) 1 and 2 [22]. These tests were specifically chosen to assess the cognitive domains reported to be predominantly affected in chronic HIV infection [23] and were feasible to undertake within a multicentre clinical study.

Subjects also self-completed an anxiety and depression score questionnaire (EQ-5D, [24]) selecting one of the three following options: 1) I am not anxious or depressed, 2) I am moderately anxious or depressed, 3) I am extremely anxious or depressed.

### Normative standards

Raw scores for each cognitive test were transformed to *z*-scores using the manufacturers' normative data [20], [21], [22] matched for each participant by age (for all tests) and also by years of education (only for the CTT). These reference data were of US origin and in general derived from a large number of subjects (over 1000) of predominantly Caucasian ethnicity. Additionally we utilise normative data considering ethnicity which were available for the CTT [22] and the HVLT domains [25]: Here, normative data were provided separately for subjects of Caucasians and Black (African-American) ethnicity although subject numbers for the latter group (approximately 150 control subjects) were smaller. In addition, for HVLT and CTT these alternative normative data did not distinguish between age groups and years of education, respectively, and for CTT the age categories for Black ethnicity did only cover the range of 20 to 50 years.

### Statistical analyses

Only subjects in whom full NC testing results were available from all 5 domains were included in the analyses. Baseline patient characteristics, and ART and HIV disease histories for all eligible subjects were tabulated separately for subjects of Caucasian and Black ethnicity. We calculated the CNS Penetration Effectiveness (CPE) score based on the 2010 scoring system for study baseline antiretroviral regimens [26] and calculated each subjects 10 year cardiovascular risk [27].

For each cognitive domain, *z*-scores were calculated by subtracting the mean and dividing by the standard deviation (SD) of test scores in reference populations using both the standard and demographically adjusted normative datasets. The z-score for the fine motor skills domain was obtained by taking the average of the z-scores for the dominant and non-dominant hands on the Grooved Pegboard tests. Global *z*-scores (NPZ-5) were derived from averaging the 5 domains. For all individual test z scores and the NPZ-5, scores above zero denote above-average neurocognitive function and scores below zero denote below-average neurocognitive function compared to the reference population. Z scores of each individual test were dichotomised based on a threshold of one SD below the mean of the normative dataset (i.e. <−1).

In addition we used two measures for defining abnormal NC function. Firstly, the percentage of subjects having a global NPZ-5<−1. Secondly, we calculated the percentage of subjects with test scores <−1 in at least two individual domains, corresponding to a previously published definition, frequently known as the Frascati score [10]. We categorised subjects into having an ‘abnormal Frascati score’ if at least two cognitive domains scored <−1 or otherwise categorised as having a ‘normal Frascati score’. As no formal activities of daily living scale was assessed, we were not able to subcategorise subjects in the ANI, MCD and HAD categories.

Prevalence of abnormal NC function using these measures were calculated using standard and ethnicity adjusted normative data, for the overall study population, and separately for subjects of Caucasian and Black ethnicity.

Proportions were compared using chi-square test. Simple comparisons of test scores were made using Mann-Whitney or Kruskal-Wallis rank tests. We used univariable and multivariable linear regression models to identify baseline factors associated with test scores (NPZ-5, z-scores), investigating gender, age, ethnicity, years of education, nadir and current CD4+ T-cell count, time since HIV RNA suppression, years on ART, current use of NNRTIs, current or past use of efavirenz, CPE score of current regimen, current and past smoking, cardiovascular (CV) risk, presence of hepatitis C virus (HCV) antibodies, baseline haemoglobin, and anxiety/depression level.

## Results

### Patient characteristics

Full NC results were available for 557 of the 587 subjects enrolled into PIVOT with baseline characteristics shown in *Table 1*. Of interest, and of potential clinical relevance, although current CD4+ lymphocyte count (mean, SD) was relatively high at 553 (217) cells/µL, nadir CD4+ lymphocyte count was 177 (117) cells/µL. Subjects had been receiving cART for a mean of 5 (SD 3) years with 53% of the cohort receiving an NNRTI based regimen and 39% receiving an efavirenz containing regimen at baseline. Thirty-one per cent of subjects reported moderate anxiety or depression with only 2% of subjects reporting extreme anxiety.

**Table 1:** Baseline characteristics, overall, and separately for subjects of Caucasian and Black ethnicity.

Variable	Overall^§^ (n = 557)	Caucasian ethnicity (n = 381)	Black ethnicity (n = 156)
Gender, male	427 (77)	353 (93)	61 (39)
Age, years	44 (9)	45 (10)	44 (8)
Nadir CD4+, cells/µL	177 (117)	189 (117)	147 (109)
Baseline CD4+, cells/µL	553 (217)	569 (219)	512 (205)
Years undetectable HIV RNA	4 (3)	4 (3)	4 (3)
Years education	15 (4)	15 (4)	15 (4)
Years on cART	5 (3)	5 (3)	5 (3)
on NNRTI	296 (53)	187 (49)	102 (65)
Efavirenz			
never on	217 (39)	147 (39)	59 (38)
not currently on but previously	125 (22)	93 (24)	27 (17)
currently on	215 (39)	141 (37)	70 (45)
efavirenz ever stopped due to CNS problems^†^	67 (12)	55 (14)	11 (7)
baseline CPE score	7 (1)	7 (1)	8 (1)
Smoker			
never	261 (47)	143 (38)	113 (72)
current	157 (28)	135 (35)	17 (11)
ex-smoker	139 (25)	103 (27)	26 (17)
CV risk			
<10%	341 (61)	199 (52)	127 (82)
10–20%	187 (34)	158 (41)	26 (17)
>20–30%	28 (5)	24 (6)	2 (1)
Hepatitis C antibody positive	21 (4)	18 (5)	2 (1)
Baseline haemoglobin, g/dL	14 (1)	15 (1)	13 (1)
Anxiety/Depression			
not anxious/depressed	369 (67)	249 (66)	106 (70)
moderately anxious/depressed	173 (31)	121 (32)	46 (30)
extremely anxious/depressed	9 (2)	9 (2)	0

*Table 1* *legend*: ^§^only patients with complete neurocognitive data (including patients of ethnicity other than Caucasian or Black, n = 20); ^†^5/67 (all Caucasian) were back on efavirenz at baseline; cART  =  combination antiretroviral therapy, CPE  =  Clinical Penetration Effectiveness, CV  =  cardiovascular, NNRTI  =  non-nucleoside reverse-transcriptase inhibitor; Data are number (%) or mean (standard deviation).

The majority of subjects were of Caucasian (68%) or Black (28%) ethnicity with only 20 subjects (4%) of other ethnicities. In general, baseline characteristics were similar between these groups apart from the much higher proportion of men in the Caucasian group.

### Neurocognitive test results utilising standard normative data

Using the standard normative dataset, median (IQR) NPZ-5 scores was −0.5 (−1.2, −0) overall, and −0.3 (−0.7, 0.1) and −1.4 (−2, −0.8), respectively, in subjects of Caucasian and Black ethnicity (*Table 2*). In all ethnic groups, NPZ-5 scores were significantly below 0 (P<0.001 in all). Overall, 32% of subjects had a global NPZ-5 score <−1, with a significantly different prevalence in Caucasian and Black subjects (17% vs. 67%; *P*<0.001). When assessing the number of subjects with an abnormal Frascati score a similar trend was observed, although proportions of abnormal findings were generally higher, with 51%, 38% and 81%, respectively, of subjects overall, of Caucasian and of Black ethnicity (*P*<0.001 for difference between Caucasian and Black ethnicity).

**Table 2:** Neurocognitive testing results.

Parameter		Overall^§^ (n = 557)	Caucasian ethnicity (n = 381)	Black ethnicity (n = 156)	Other ethnicity (n = 20)	*P-value for difference between* C*aucasian vs. Black ethnicity*
**Standard normative data**	NPZ-5 score	−0.5 (−1.2, −0) [95%CI −0.8, −0.6]	−0.3 (−0.7, 0.1) [95%CI −0.4, −0.3]	−1.4 (−2, −.8) [95%CI −1.8, −1.4]	−0.8 (−1.3, −0.4) [95%CI −1.3, −0.6]	<*0.001*
	Overall NPZ-5 score >1 SD below population mean	179 (32)	66 (17)	104 (67)	9 (45)	<*0.001*
	At least 2 individual tests with *z*-score >1 SD below population mean	285 (51)	144 (38)	127 (81)	14 (70)	<*0.001*
**Adjusted normative data^¶^**	NPZ-5 score		−0.8 (−1.3, −0.3)	−1.1 (−1.6, −0.6)		<*0.001*
	Overall NPZ-5 score>1 SD below population mean		145 (38)	69 (55)		*0.001*
	At least 2 individual tests with *z*-score>1 SD below population mean		236 (62)	86 (68)		*0.20*

*Table 2* *legend*: ^§^patients with complete neurocognitive data including patients of ethnicity other than Caucasian or Black (n = 20); IQR  =  interquartile range, SD  =  standard deviation; ^¶^Excludes all patients of ethnicity other than Caucasian and Black, as well as 30 subjects of Black ethnicity who fell outside the age range covered by the adjusted normative data; NPZ-5 results using standard normative data in these 30 patients were similar to those of the other 126 patients. Data are number (%) or median (interquartile range).

**Figure 1:**
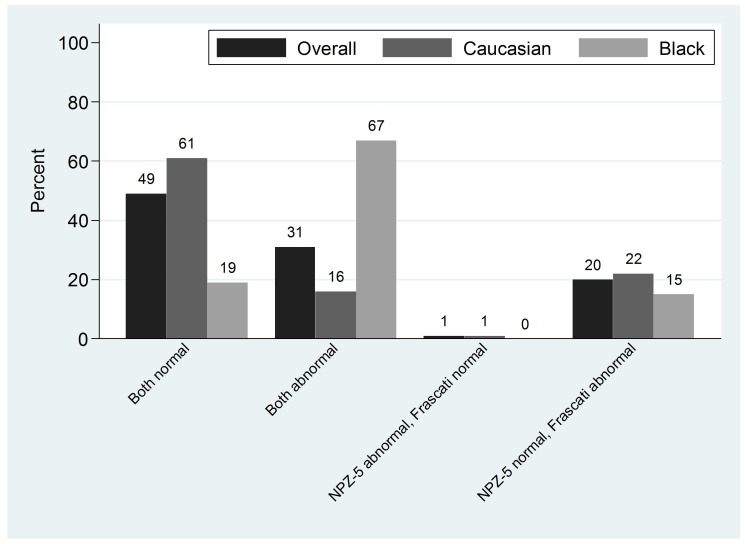
Association between NPZ-5 score and Frascati score.

In 80% of subjects overall, the two definitions of impairment were in agreement (*Figure 1*), and NPZ-5 score and the Frascati criteria were either both ‘normal’ (48%) or both ‘abnormal’ (31%). In 1% of subjects, NPZ-5 score was<−1 but Frascati criteria were classified as normal, and vice versa in 20%. Hence, discordance between findings based on NPZ-5 scores and the Frascati criteria was predominantly due to an abnormal Frascati score definition in the presence of a NPZ-5 score which was above our cut-off of 1 SD below normative means. Agreement was 77% in Caucasians, and 85% in patients of Black ethnicity.

### Factors associated with global cognitive scores

NPZ-5 scores were not quite normally distributed (slightly skewed to the left). However, differences between ethnic groups were very similar using univariable linear regression compared to the statistics given in *Table 2*. These differences between ethnic groups remained nearly unchanged in a multivariable model, that is after adjustment for demographic and clinical variables and other potential influence factors of NC performance. Both Black (−1.1 score points; 95% CI −1.3 to −0.9) and other ethnicity (−0.6 score points; 95% CI −1.0 to −0.3) were associated with poorer NPZ-5 scores compared with Caucasians (overall P value for ethnic group <0.001).

Because of an apparent influence of the reference data used (see below), we decided to assess factors associated with NPZ-5 scores separately for subjects of Caucasian and Black ethnicity; numbers for other ethnicity were considered too small for meaningful multivariable analyses.

In subjects of Caucasian ethnicity, current nevirapine use (0.34 score points less in those on nevirapine; p = 0.021) and HCV (0.33 score points less in those with detectable antibodies; p = 0.047) were associated with lower NPZ-5 scores (*Table 3*). Of note, the association with nevirapine was adjusted for previous cessation of efavirenz due to CNS toxicity, and results were very similar when instead adjusting for never, current, previous use of efavirenz (i.e. ignoring recorded cause of cessation). 20/43 of patients currently on nevirapine have been on efavirenz before, of which 12 had stopped efavirenz due to CNS toxicity, however, in patients on nevirapine adjusted NPZ-5 scores were −0.63 and −0.54, respectively, in those never and previously on efavirenz. Therefore, association with nevirapine was unlikely being confounded by a previous switch from efavirenz.

**Table 3:** Linear regression analysis assessing factors associated with NPZ-5 scores.

	Univariable				Multivariable			
Study parameter	Caucasian ethnicity		Black ethnicity		Caucasian ethnicity		Black ethnicity	
	*Coef. [95% CI]*	*P*	*Coef. [95% CI]*	*P*	*Coef. [95% CI]*	*P*	*Coef.*	*P*
Gender, female vs. male	0.06 [−0.19, 0.32]	0.63	−0.10 [−0.40, 0.21]	0.54	0.03 [−0.25,0.32]	0.83	−0.05 [−0.49,0.40]	0.83
Anxious/depressed at baseline (moderately or extremely vs. Not)	−0.12 [−0.26, 0.02]	0.09	−0.00 [−0.33, 0.32]	0.99	−0.11 [−0.25,0.04]	0.14	0.04 [−0.32,0.40]	0.83
Efavirenz, on at baseline vs. not on	0.04 [−0.09, 0.18]	0.54	−0.12 [−0.41, 0.18]	0.44	−0.05 [−0.22,0.11]	0.53	−0.25 [−0.62,0.12]	0.19
Nevirapine, on at baseline vs. not on	−0.28 [−0.49, −0.07]	**0.009**	−0.07 [−0.44, 0.31]	0.72	−0.34 −0.63, −0.05	**0.021**	−0.08 [−0.62,0.47]	0.79
Ever stopped efavirenz due to CNS problems, (yes v. No)	0.07 [−0.12, 0.26]	0.49	−0.23 [−0.80, 0.34]	0.42	0.09 [−0.11,0.29]	0.39	−0.16 [−0.79,0.47]	0.62
Smoker, vs. never								
current smoker	0.02 [−0.14, 0.18]	0.80	0.63 [0.17, 1.10]	**0.008**	0.05 [−0.13,0.22]	0.60	0.68 [0.11,1.25]	**0.020**
ex-smoker	0.13 [−0.04, 0.30]	0.13	0.30 [−0.09, 0.70]	**0.13**	0.12 [−0.05,0.29]	0.17	0.37 [−0.08,0.82]	**0.11**
Cardiovascular risk, > = 10% vs. <10%	−0.06 [−0.20, 0.07]	0.36	−0.01 [−0.40, 0.38]	0.96	−0.05 [−0.20,0.10]	0.53	−0.32 [−0.80,0.15]	0.18
Hepatitis C antibody, positive vs. negative	−0.30 [−0.61, 0.01]	0.061	−0.44 [−1.74, 0.86]	0.51	−0.33 [−0.65, −0.00]	**0.047**	−0.26 [−1.63,1.11]	0.70
Prior AIDS illness, yes vs. no	0.05 [−0.13, 0.23]	0.58	−0.10 [−0.45, 0.25]	0.58	0.02 [−0.17,0.22]	0.82	−0.12 [−0.54,0.30]	0.57
Age, per 10 years	−0.05 [−0.12, 0.02]	0.14	−0.06 [−0.25, 0.13]	0.55	−0.05 [−0.13,0.03]	0.21	−0.04 [−0.26,0.18]	0.74
CD4+ nadir, per increasing 100 cells/µL	−0.03 [−0.09, 0.02]	0.26	0.00 [−0.13, 0.14]	0.97	−0.01 [−0.08,0.06]	0.81	−0.09 [−0.26,0.09]	0.34
CD4+ at baseline, per increasing 100 cells/µL	−0.03 [−0.06, 0.00]	0.06	−0.00 [−0.07, 0.07]	0.95	−0.02 [−0.06,0.01]	0.20	0.01 [−0.09,0.11]	0.80
Years with undetectable HIV RNA	−0.01 [−0.03, 0.01]	0.45	−0.04 [−0.10, 0.02]	0.19	0.00 [−0.03,0.04]	0.90	−0.04 [−0.13,0.05]	0.34
Years of known HIV infected	−0.01 [−0.02, 0.01]	0.41	−0.00 [−0.04, 0.04]	0.995	0.00 [−0.01,0.02]	0.60	0.03 [−0.03,0.09]	0.34
Years of education	−0.08 [−0.17, 0.01]	0.10	0.03 [−0.16, 0.22]	0.75	−0.02 [−0.04,0.00]	0.089	0.00 [−0.04,0.05]	0.91
Number of different ART drugs received	−0.02 [−0.06, 0.02]	0.31	−0.12 [−0.21, −0.02]	**0.021**	−0.02 [−0.07,0.03]	0.53	−0.09 [−0.22,0.04]	0.17
Haemoglobin, per g/dl	0.01 [−0.05, 0.07]	0.80	0.05 [−0.06, 0.15]	0.39	0.02 [−0.05,0.09]	0.60	−0.00 [−0.15,0.14]	0.97
CPE score of regimen	−0.02 [−0.09, 0.05]	0.59	−0.00 [−0.14, 0.14]	0.99	0.03 [−0.06,0.13]	0.50	−0.05 [−0.24,0.15]	0.62

*Table 3* *legend*: CI  =  confidence interval; CPE  =  Clinical Penetration Effectiveness; NPZ-5 = mean neuropsychiatric z-score for 5 domains; *Coef* = regression coefficient. P values (*P*)<0.05 shown in bold. ^¶^Overall p-value for smoking in subjects of Black ethnicity 0.016 (univariable) and 0.022 (multivariable).

In subjects of Black ethnicity, smoking was the only factor associated with NPZ-5 scores: current and ex-smokers had 0.68 and 0.37, respectively, score points higher than subjects who never smoked (overall p = 0.022) (*Table 3*).

Other HIV disease factors such as nadir CD4+ lymphocyte count and antiretroviral factors such as CPE score were not associated with NPZ-5 score in either ethnic group.

### Neurocognitive test results utilising demographically adjusted normative data

Using the demographically adjusted normative data, the differences between ethnicities were less marked with poorer results for subjects of Caucasian ethnicity and better results for subjects of Black ethnicity compared with using the standard normative data (*Table 2*). Although a statistically significant difference in the number of subjects with NPZ-5 scores<−1 remained between ethnicities (*P* = 0.001), the difference was no longer statistically significant in numbers of subjects meeting the Frascati criteria (P = 0.20). Of note, whereas there was a significant difference in NPZ-5 scores between Caucasian and Black subjects in univariable regression analysis (p<0.001), the difference disappeared-in contrast to the analysis using the standard normative dataset-when adjusting for demographic, clinical and other factors (p = 0.31).

## Discussion

We have assessed NC function in a large group of HIV infected subjects on effective cART using a standard cognitive testing battery. Although this is not the same as a formal neurophyschological assessment, the brief neurocognitive test battery we used comprised validated components of standard formal neuropsychological tests [28] and we have made several interesting and novel observations relevant to both current clinical practice and future research in this field.

In our study, the median global NPZ-5 score for Caucasian subjects, using the standard normative control data, was −0.3 and therefore slightly lower than 0 which to be expected in a normal population. However, these data are reassuring insofar as the difference is quite small and unlikely to be functionally significant [29], suggesting that in effectively treated HIV infected subjects global cognitive function is similar to normative control data. Furthermore, the small difference may be driven by factors other than HIV infection that can influence neurocognitive function such as differences in rates of smoking that may differ from the normative dataset. We did not have an HIV-negative comparison group in our study which might have provided further clarity on this. Our study differs from other reports where poorer global NPZ-5 scores in HIV infected cohort are described [5], [6]. We postulate these differences may in part be due to differences in the populations studied. In our study, all subjects were virologically suppressed at the time of study and had been so for a mean of 4 years. Previous studies have reported improvements in cognitive function in HIV infected subjects commencing antiretroviral therapy with the dynamic of these improvements evident for at least a one year period [15], [30]. Although we did not find evidence for this in the model, it may be that improvements in cognitive function may continue for many years until global cognitive scores in a group of patients may start to match control population values.

Despite global NPZ-5 scores in Caucasian subjects being similar to the standard normative control data, high rates of abnormalities were observed when we assessed results using the Frascati criteria with 37% of subjects having an abnormal Frascati score in this group. These results differ from a recent study conducted in the UK where *Garvey et al* described only 19% of Caucasian individuals to have an abnormal Frascati score [31]. In this study, global NPZ scores were also similar to population control data. The differences may be due to the patient selection and nature of the testing performed but it is also possible that these differences could be due to the normative datasets used in our study compared to the study conducted by *Garvey* et al. In our study we undertook traditional NC testing where the normative control dataset originate from historic US cohorts, some dating from the 1960s and 1970s. *Garvey* et al undertook computerised NC testing (CogState™, Melbourne, Australia) where the control population are predominantly Caucasian male Australian subjects recruited within the last decade. With cultural changes over time, it is possible that the CogState™ control dataset is more representative of our Caucasian cohort recruited over the past few years in the UK and therefore when a NC score is calculated which encompasses results from several cognitive domains, such as the Frascati score, such differences in results become apparent.

We observed poorer results within individual NC domain scores (data not shown), global NPZ-5 scores and the categorical score using the Frascati criteria in our study for subjects of Black ethnicity compared to subjects of Caucasian ethnicity. There are several plausible explanations for these findings. All tests were undertaken using instructions in English and it is possible language difficulties could account in part for these differences. However we do not believe this to be an important factor because the differences were no more marked on the HVLT tests, which are highly language-dependent, than they were on the other tests which do not depend on linguistic fluency. Furthermore, the majority of the Black patients attending clinics in the UK are either born in the UK, or are immigrants from African countries where English is widely spoken, and patients who were not able to understand the study instructions were not included. Patients who appeared to understand the instructions but then performed the tests incorrectly would also have been excluded as the tests would have been deemed invalid. Differences in HIV disease factors or other characteristics at baseline could also explain such differences. However, baseline characteristics were similar for subjects of Caucasian and Black ethnicity in many areas, and the influence of ethnicity persisted after adjusting for baseline factors.

We hypothesise the differences in NC test results between the ethnicities observed in our study are due to differences in the characteristics of control datasets available for use for several reasons. Firstly, when utilising the demographically adjusted normative dataset, the differences between ethnicities become less marked and indeed no statistically significant difference is observed in the Frascati criteria definition between Caucasian and Black ethnicities when we use this adjusted normative data. Secondly, as described above, for Caucasian subjects, in other studies using different normative datasets [31], [32], quite different results are observed. Lastly, results of NC testing from control datasets which are recruited specifically to match HIV infected cohorts are described to differ substantially from traditional population control datasets [13]. Indeed the effects of ethnicity on cognitive function and lack of appropriate control data have previously been described in a female HIV-infected cohort [13].

A finding from our study which remains challenging is to explain the poorer NC testing results observed in Caucasian subjects when using the demographically adjusted dataset compared to the standard normative dataset. Again this may be related to differences in the Caucasian controls used within these control datasets however this area requires further investigation.

Nadir CD4+ lymphocyte count has been associated with greater risk of NC impairment in many reports [6], [31], [33], a finding we did not observe within our cohort. The longer duration of effective antiretroviral therapy and a lack of historic documented virological failure differs from other studies and is a possible explanation.

We observed an association between current nevirapine use in Caucasian subjects and poorer NC function which has not been previously described. This is an unexpected finding as nevirapine is considered to have pharmacokinetic exposure at effective concentrations in the cerebrospinal fluid [34] and no *in vitro* data to our knowledge have reported any neuronal toxicities associated with nevirapine. We believe this may be a channelling bias whereby subjects with pre-existing NC impairment or psychiatric conditions are preferentially commenced on nevirapine. In our multivariate model, ever stopping efavirenz due to CNS toxicities was not associated with global NPZ-5 scores, which may suggest the association we have observed with nevirapine is not associated with subjects switching to nevirapine from other cART regimens with overt CNS toxicities. However this association does not address the channelling bias associated with commencing a nevirapine regimen *de novo* in subjects previously naive to antiretroviral therapy.

Several findings from our study are highly relevant to this field of HIV medicine. Of important to clinicians caring for HIV infected individuals and for HIV community groups, our description of a large cohort where overall NC function in subjects on cART for many years is similar to a matched HIV seronegative control population, albeit only within the Caucasian cohort in whom we believe the matched population datasets are the most relevant, is reassuring. Although we are not able to tease out pathogenic mechanisms associated with cerebral dysfunction, we are able to provide reassuring data that in general NC function within a well cared for cohort, does not substantially differ from a control population. Another seminal finding from our work for future clinical research, is the importance of recruiting well matched control populations to the HIV infected populations being studied, in order to aid the interpretation of study findings and in order to assess if findings are related to HIV-disease itself or the cohorts being studied.

## Supporting Information

Protocol S1 (PDF)
