# Supplementary material for: Neurocognitive Function in HIV Infected Patients on Antiretroviral Therapy
Source: PLoS One. 2013 Apr 30;8(4):e61949. doi: 10.1371/journal.pone.0061949 (PMC3639991; doi:10.1371/journal.pone.0061949)
Supplement: Protocol S1 — (PDF) [file pone.0061949.s001.pdf]

PIVOT

Protease Inhibitor monotherapy Versus Ongoing Triple-therapy in the long-term management of HIV infection

Full title: A randomised controlled trial of a strategy of switching to boosted protease inhibitor monotherapy versus continuing combination antiretroviral therapy for the long-term management of HIV-1 infected patients who have achieved sustained virological suppression on highly-active antiretroviral therapy

EUDRACT: 2007-006448-23
ISRCTN04857074

Version 2.0, 18 November 2008

Authorised by:

Name:	Dr N Paton	Role:	Chief Investigator
Signature:	[signature]	Date:	19 November 2008
Name:	Dr D Dunn	Role:	Trial Statistician
Signature:	[signature]	Date:	19/11/2008

[Page 2 intentionally blank]

GENERAL INFORMATION

This document describes the PIVOT trial and provides information about procedures for entering patients into the trial. The protocol should not be used as an aide-memoire or guide for the treatment of other patients; every care was taken in its drafting, but corrections or amendments may be necessary. These will be circulated to the registered investigators in the trial, but centres entering patients for the first time are advised to contact the HIV Group, Medical Research Council Clinical Trials Unit, London, to confirm they have the most up-to-date version. Clinical problems relating to this trial should be referred to the Chief Investigator.

- **Compliance**
  The trial will be conducted in compliance with the protocol, MRC GCP, Data Protection Act (DPA number: Z5886415), NHS research governance and other regulatory requirements, as appropriate in the participating centres.

- **Sponsor**
  Medical Research Council, 20 Park Crescent Road, London W1B 1AL, UK.

- **Funder**
  NHS R&D Health Technology Assessment (HTA) Programme, Mailpoint 728, Boldrewood, University of Southampton, Southampton SO16 7PX, UK.

Main Contacts

**Emergency medical contact**	Dr Nicholas Paton<br>MRC Clinical Trials Unit<br>222 Euston Road<br>London NW1 2DA	Email:<br>Tel:<br>Fax:	nick.paton@ctu.mrc.ac.uk<br>020 7670 4808<br>020 7670 4817
**Chief Investigator**	Dr Nicholas Paton<br>MRC Clinical Trials Unit<br>222 Euston Road<br>London NW1 2DA	Email:<br>Tel:<br>Fax:	nick.paton@ctu.mrc.ac.uk<br>020 7670 4808<br>020 7670 4817
**Trial Statisticians**	Dr David Dunn<br>MRC Clinical Trials Unit<br>222 Euston Road<br>London NW1 2DA	Email:<br>Tel:<br>Fax:	david.dunn@ctu.mrc.ac.uk<br>020 7670 4739<br>020 7670 4818
	Dr Wolfgang Stöhr<br>MRC Clinical Trials Unit<br>222 Euston Road<br>London NW1 2DA	Email:<br>Tel:<br>Fax:	wolfgang.stoehr@ctu.mrc.ac.uk<br>020 7670 4802<br>020 7670 4818
**Trial Physician**	Dr Alejandro Arenas-Pinto<br>MRC Clinical Trials Unit<br>222 Euston Road<br>London NW1 2DA	Email:<br>Tel:<br>Fax:	AAP@ctu.mrc.ac.uk<br>020 760 4716<br>020 760 4817

**Trial Virologist**	Professor Deenan Pillay<br>Centre for Virology<br>UCL Windeyer Institute<br>46 Cleveland St<br>London W1T 4JF	Email:<br>Tel:<br>Fax:	d.pillay@ucl.ac.uk<br>020 7679 9482/9490 (Office)<br>020 7580 5896
**Trial Pharmacologist**	Dr Saye H Khoo<br>Dept of Pharmacology<br>University of Liverpool<br>70 Pembroke Place<br>Liverpool L69 3GF	Email:<br>Tel:<br>Fax:	S.H.Khoo@liverpool.ac.uk<br>0151 794 5560<br>0151 794 5656
**Health Economist**	Professor Mark Sculpher<br>Centre for Health Economics<br>University of York<br>Heslington<br>York YO10 5DD	Email:<br>Tel:<br>Fax:	mjs23@york.ac.uk<br>01904 321440<br>01904 321402
**External Principal Investigator**	Professor Brian Gazzard<br>St Stephens Centre<br>Chelsea and Westminster Hospital<br>369 Fulham Road<br>London SW10 9NH	Email:<br>Tel:<br>Fax:	Yvonne.Stupple@chelwest.nhs.uk<br>020 8746 8239<br>020 8746 5611
**Trial Management**	Fionna van Hooff<br>MRC Clinical Trials Unit<br>222 Euston Road<br>London NW1 2DA	Email:<br>Tel:<br>Fax:	pivot@ctu.mrc.ac.uk<br>020 7670 4843<br>020 7670 4817
	Ellen Dyer<br>MRC Clinical Trials Unit<br>222 Euston Road<br>London NW1 2DA	Email:<br>Tel:<br>Fax:	pivot@ctu.mrc.ac.uk<br>020 7670 4926<br>020 7670 4817

For the listing of participating institutions and clinicians please refer to Appendix 4.

RANDOMISATION

Tel: +44 (0)20 7670 4843 **(Mon - Fri, 09:00 – 17:00)**
Fax: +44 (0)20 7670 4817

SUSAR NOTIFICATION

Within one working day of becoming aware of an SUSAR, please fax a completed Event CRF to the MRC Clinical Trials Unit on:

Fax: +44 (0)20 7670 4817

CONTENTS

1. Summary 9
1.1 Background and Aims 9
1.2 Flow diagram 10
1.3 Trial schedule 11

2. Background 12
2.1 Introduction 12
2.2 Managing HIV as a long-term chronic disease: changes in the treatment paradigm . 12
2.3 Previous trials of PI monotherapy 13
2.4 Rationale and objectives of this trial 14
2.5 Risks and benefits of the PI monotherapy strategy used in this trial 15

3. Selection of Centres & Clinicians 18
3.1 Criteria for selection of trial sites & clinicians 18
3.2 Site responsibilities 18
3.3 Site approval 19

4. Selection of Patients 20
4.1 Patient inclusion criteria 20
4.2 Patient exclusion criteria 20
4.3 Screening procedures and pre-randomisation investigations 21

5. Randomisation & Enrolment 23
5.1 Randomisation visit (Baseline; Day 0) 23
5.2 Procedure for randomisation 23
5.3 Post-randomisation procedures and follow-up 24
5.4 Co-enrolment guidelines 24

6. Treatment of Patients 25
6.1 Introduction 25
6.2 Control arm 25
6.3 PI monotherapy arm 26
6.4 Management of drug toxicity and adverse events 28
6.5 Management of cardiovascular disease risk factors 28
6.6 Concomitant medication 29
6.7 Drug supply, dispensing and accountability 29
6.8 Adherence assessment and counselling 30

7. Assessments & Follow-up 31
7.1 Routine Follow-up 31
7.2 Procedures for assessing efficacy 35
7.3 Procedures for assessing safety 37
7.4 Procedures for assessing health economics 38
7.5 Trial closure 38

8. Loss to Follow-up & Withdrawal 39
8.1 Patient transfers 39
8.2 Loss to follow-up 39
8.3 Withdrawal of consent 39

9. Statistical Considerations 40
9.1 Method of randomisation 40
9.2 Outcome measures 40
9.3 Sample size 41
9.4 Interim monitoring and analyses 42

9.5 Analysis plan 42
10. Trial Monitoring 44
10.1 Risk assessment 44
10.2 Monitoring at MRC CTU 44
10.3 Clinical site monitoring 44
10.4 Data quality assurance 44
10.5 Confidentiality of trial documents and patient records 45
11. Safety Reporting 46
11.1 Definitions 46
11.2 Institution/Investigator responsibilities 47
11.3 MRC CTU responsibilities 48
12. Ethical Considerations & Approval 50
12.1 Ethical considerations 50
12.2 Ethical approval 50
13. Regulatory issues 52
14. Indemnity 53
14.1 Liability 53
15. Finance 54
16. Trial Committees 55
16.1 Trial Management Group 55
16.2 Trial Steering Committee 55
16.3 Independent Data Monitoring Committee 55
16.4 Endpoint Review Committee 55
17. Publication 56
18. References 57
Appendices 60

LIST OF FIGURES

Figure 1: Trial entry, randomisation and treatment 10
Figure 2: Safety Reporting Flowchart 49
Figure 3: Diagram of relationships between trial committees 55

LIST OF TABLES

Table 1: Safety Reporting Definitions 46
Table 2: Definitions of causality 47

APPENDICES

APPENDIX 1: PATIENT INFORMATION SHEET
APPENDIX 2: CONSENT FORM
APPENDIX 3: GP LETTER
APPENDIX 4: PARTICIPATING SITES
APPENDIX 5: EQ-5D QUESTIONNAIRE
APPENDIX 6: MOS-HIV QUESTIONNAIRE

APPENDIX 7: TOXICITY TABLE
APPENDIX 8: DIAGNOSTIC CRITERIA FOR SERIOUS AIDS-DEFINING ILLNESS
APPENDIX 9: DIAGNOSTIC CRITERIA FOR SERIOUS NON-AIDS-DEFINING ILLNESS
APPENDIX 10: PROTEASE INHIBITOR PRODUCT INFORMATION
APPENDIX 11: JOINT BRITISH SOCIETIES CARDIOVASCULAR RISK PREDICTION CHARTS
APPENDIX 12: LABORATORY METHODS & SPECIMEN STORAGE
APPENDIX 13: NEUROCOGNITIVE ASSESSMENT TOOLS
APPENDIX 14: PROTOCOL VERSION 2.0 LISTING OF CHANGES

ABBREVIATIONS AND GLOSSARY

**AE**	Adverse event
**ACTG**	AIDS Clinical Trials Group
**AIDS**	Acquired immune deficiency syndrome
**ALT**	Alanine transaminase
**AR**	Adverse reaction
**ART**	Antiretroviral therapy
**AST**	Alanine aminotransferase
**BHIVA**	British HIV Association
**BNF**	British National Formulary
**CCR5**	Chemokine (C-C motif) receptor 5
**CDC**	Center for Disease Control and prevention
**CDSC**	Communicable Disease Surveillance Centre
**CHM**	Commission on Human Medicine
**CF**	Consent form
**CG**	Cockcroft-Gault equation
**CI**	Chief Investigator
**CRF**	Case Report Form
**CT**	Computerised (Axial) Tomography scan
**CTA**	Clinical Trials Authorisation
**CTU**	Clinical Trials Unit
**DoH**	Department of Health
**ECG**	Electrocardiogram (also EKG)
**EDTA**	Ethylenediaminetetraacetic acid
**ELISA**	Enzyme-Linked ImmunoSorbent Assay
**EQ-5D**	Euroqol (five-domain) health status questionnaire
**ERC**	Endpoint Review Committee
**EUDRACT**	European Union Drug Regulatory Agency Clinical Trial
**GP**	General Practitioner
**HAART**	Highly Active Antiretroviral Therapy
**HDL**	High-density lipoprotein
**HE**	Health Economics
**HIV**	Human Immunodeficiency Virus
**HTA**	Health Technology Assessment
**IB**	Investigator's Brochure
**HVLT-R**	Hopkins Verbal Learning Test - revised
**ICH GCP**	International Conference on Harmonisation Good Clinical Practice
**IDMC**	Independent Data Monitoring Committee
**IMP**	Investigational Medicinal Product
**ISRCTN**	International standard randomised controlled trial number
**ITT**	Intention to treat
**KS**	Kaposi's sarcoma
**LDL**	Low-density lipoprotein
**LFT**	Liver function tests

**LREC**	Local research ethics committee
**MHRA**	Medicines and Healthcare Regulatory Authority
**MHS**	Mental health summary score
**MI**	Myocardial infarction
**MOS HIV**	Medical Outcomes Study HIV Health Survey
**MRC**	Medical Research Council
**MRI**	Magnetic resonance imaging
**NARS**	Neuropsychiatric AIDS Rating Scale
**NHS**	National Health Service
**NRTI**	Nucleoside reverse transcriptase inhibitors
**NNRTI**	Non-nucleoside reverse transcriptase inhibitors
**OT**	On-treatment
**PHS**	Physical health summary score
**PI**	Protease Inhibitor
**PIS**	Patient Information Sheet
**QALY**	Quality-adjusted life-year
**QoL**	Quality of Life
**R&D**	Research and Development
**REC**	Research Ethics Committee
**RNA**	Ribonucleic acid
**SAE**	Serious adverse event
**SAR**	Serious adverse reaction
**SD**	Standard deviation
**SOP**	Standard operating procedure
**SmPC**	Summary of product characteristics
**SUSAR**	Suspected unexpected serious adverse reaction
**TLOVR**	"Time to loss of virologic response"
**TDM**	Therapeutic drug monitoring
**TMG**	Trial Management Group
**TSC**	Trial Steering Committee
**UAR**	Unexpected adverse reaction
**UEC**	Urea, Electrolytes and Creatinine
**UK-CAB**	United Kingdom Community Advisory Board
**ULN**	Upper limit of normal
**VL**	Viral load
**WCC**	White cell count

1. SUMMARY

1.1 Background and Aims

The current standard-of-care treatment for people living with HIV is combination antiretroviral therapy (ART), usually consisting of 3 drugs: 2 nucleoside reverse transcriptase inhibitors (NRTIs) with either a non-nucleoside reverse transcriptase inhibitor (NNRTI) or a protease inhibitor (PI). Although triple ART has a relatively high long-term success rate, a proportion of patients (about 4% per year) continues to experience virological failure, and this is often associated with complete resistance to one or more drugs (especially NRTI and NNRTI classes). Furthermore, long-term drug toxicity remains a concern. Additional strategies for long-term management are needed that preserve future drug options and minimise toxicity. Short-term clinical trials suggest that stable patients who have had prolonged virological suppression on combination ART can be switched successfully to PI monotherapy, resulting in a reduction of toxicity without increasing the risk of treatment failure and drug resistance.

This trial aims to determine whether a strategy of switching to PI monotherapy is non-inferior to continuing triple-therapy, in terms of the proportion of patients who maintain all the drug treatment options that were available to them at baseline after at least 3 years of follow-up, and to compare clinical events, safety, toxicity and health economic parameters between the two strategies.

1.1.1 Trial design

This is a parallel group, open-label, multi-centre, randomised controlled strategy trial.

1.1.2 Patients to be included

400 patients will be included who are HIV-infected adults on a stable ART regimen of two NRTIs and one NNRTI or PI. Patients will have CD4+ T-cell counts greater than 100 cells/μL, and viral load (VL) less than 50 copies/ml for no less than 6 months. Patients who have had virological failure on any previous ART regimen, who have PI resistance mutations, or in whom PIs are contraindicated will not participate. For more details refer to section 4.

1.1.3 Trial interventions – research and control arm

Patients randomised to the PI monotherapy group will stop other ART drugs and start or continue only on a ritonavir-boosted PI (selection of drug at discretion of physician and patient). Those who do not maintain complete virological suppression or who are unable to tolerate the PI (substitution for toxicity is allowed), will promptly switch back to their previous triple-therapy. Patients randomised to the control group will continue their current regimen. For more details refer to section 6.

1.1.4 Duration of trial

The trial will be conducted over 5 years, including an initial recruitment period estimated to last 12 to 18 months. All patients will continue on treatment and follow-up until close of the trial. For more details refer to section 7.

1.1.5 Outcome measures

Analyses will compare the 2 groups by intention to treat (ITT).

Primary outcome measure:

Loss of future drug options defined as the occurrence of intermediate to high level resistance to any one or more of the standard antiretroviral drugs (limited to licensed drugs in contemporary use) to which the patient's virus was considered to be sensitive at trial entry.

Secondary outcome measures:

- Serious drug or disease-related complications
- Adverse events
- Virological rebound
- CD4+ count change
- Health-related Quality of Life change
- Neurocognitive function change
- Cardiovascular risk change
- Health care costs

For more details refer to section 9.

1.1.6 Data recorded directly on CRFs

Clinical and routine laboratory data will be recorded on case report forms (CRFs). A copy will be faxed or sent to the MRC CTU for data entry and the original will be kept at the local centre. For more details see section 7.

1.1.7 Organisation

Sponsor: Medical Research Council, UK.
Funder: NHS R&D Health Technology Assessment (HTA) programme, UK.
Coordinator: Medical Research Council Clinical Trials Unit, UK.

1.2 Flow diagram

Figure 1: Trial entry, randomisation and treatment

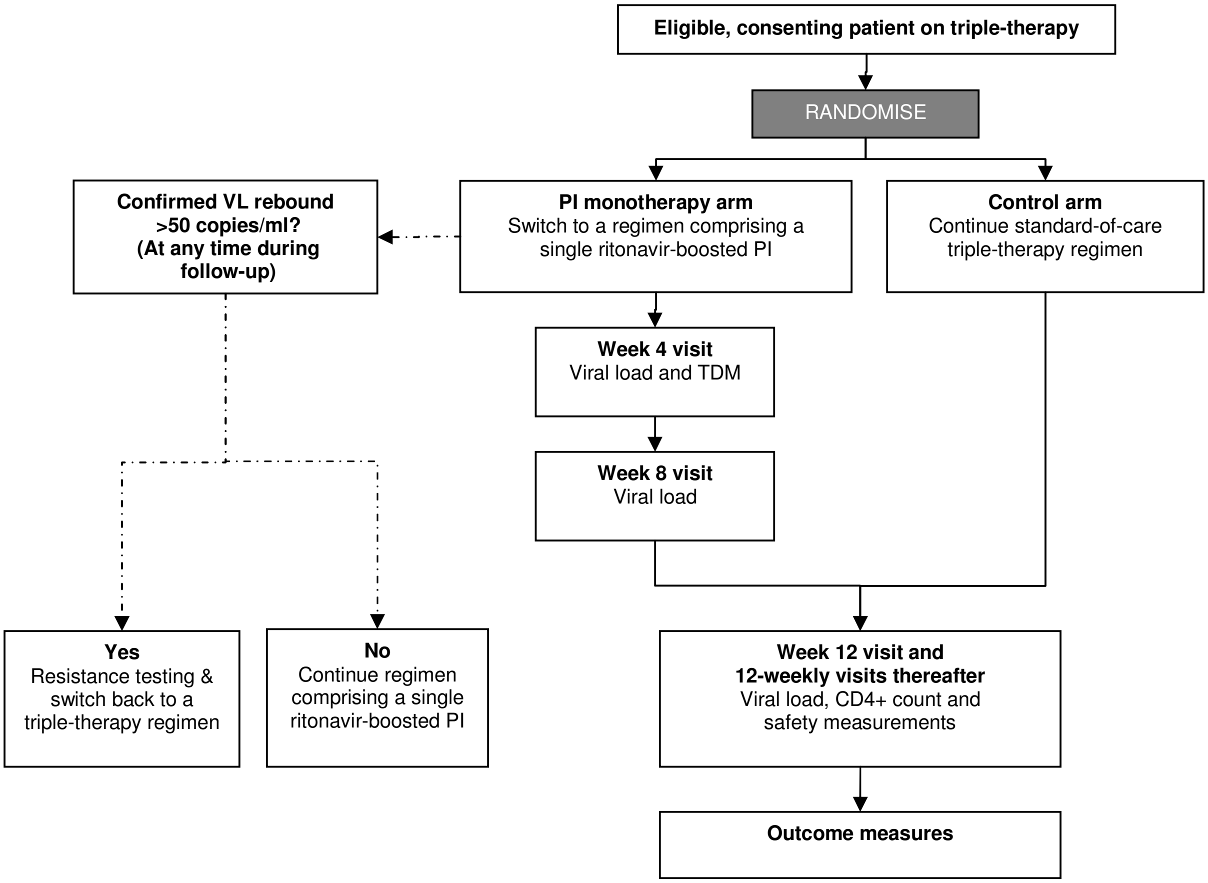

1.3 Trial schedule

**PIVOT trial schedule**	**Screening** -4wks to Day 0	**Day 0** (Baseline)	**PI monotherapy arm only**		**Week 12** +/- 2 wks	**Subsequent 12-weekly visits** Wk 24, 36 etc +/- 6 wks	**Annual visits** Wk 48, 96, 144, 192, 240 and final visit[9] +/- 6 wks
			**Week 4** +/-2 wks	**Week 8** +/- 2 wks			
Complete and sign Consent Form	X						
Medical and drug history	X						
Assessment of drug adherence		X	X	X	X	X	X
Concomitant medication		X	X	X	X	X	X
Symptom review/ physical examination	X	X	X	X	X	X	X
Assessment of clinical adverse events		X	X	X	X	X	X
Cardiovascular risk assessment	X						X
ECG (within last 6 months acceptable)	X						
Assessment of healthcare resource utilisation			X	X	X	X	X
EQ-5D health status questionnaire		X	X	X	X	X	X
Neurocognitive assessment		X			X		X
MOS-HIV questionnaire		X			X		X
HIV viral load[1,2]	X	X	X	X	X	X	X
CD4+ count	X	X			X	X	X
Full blood count, UEC and LFTs[3]	X	X	X		X	X	X
Bone profile[4]	X				X	X[4]	X
Glucose and lipids (fasting)[5]	X		X		X		X
Hepatitis B/C serology (within last 6 months acceptable)	X						X
Plasma sample for storage[6]	X	X	X		X		X
PI drug concentration[7]			X	X[8]	X[8]		
Urine protein/creatinine ratio[4]	X				X	X[4]	X
Urine albumin/creatinine ratio and phosphate	X						X
Urine sample for storage	X						X
Pregnancy test[8]	X						
Completion of CRF	X	X	X	X	X	X	X

1 HIV viral load will be repeated on the same lab sample for any value >50 copies/ml. If >50 copies/ml confirmed on lab re-test, patient will be telephoned for adherence counselling and asked to return for repeat VL test at 4 weeks (window up to 6 weeks) after the first test. Repeat plasma sample for storage will be obtained at the second visit.
2 Resistance testing will be performed on a stored sample at the local laboratory in the event of 2 consecutive VL values >50 copies/ml. Resistance testing will be performed on the higher of the two samples if possible. If VL results differ by less than 50 copies/ml the second sample will be used for resistance testing.
3 FBC will include Hb, WCC, neutrophil count, lymphocyte count, platelets; UEC will include sodium, potassium, urea, creatinine; LFTs will include bilirubin, ALT, alkaline phosphatase.
4 Bone profile (calcium and phosphate) and urine protein/creatinine ratio will be performed at screening, Week 12 and annual visits in all, but quarterly only in those patients taking tenofovir.
5 Lipids will include total cholesterol, triglycerides, LDL, HDL. If elevated at Week 12, additional fasting lipid tests can be performed between the annual visit checks as needed to inform clinical management.
6 Blood sample will be stored at screening (2X4ml EDTA), Week 12 and annual visits (1X4ml EDTA) plus any visit where a second VL sample is taken for confirmation of rebound (2X4ml EDTA).
7 PI drug concentration measurement can be repeated at Week 8 and/or Week 12 if Week 4 concentration is low (only in the PI monotherapy arm).
8 Pregnancy test in women only; can be urine or blood test, as per standard practice at site; repeated at any subsequent visits where pregnancy is suspected.
9 The final visit will be scheduled within 12 weeks before the common closing date of the trial.

2. BACKGROUND

2.1 Introduction

At the end of 2005 there were an estimated 63,500 people living with HIV in the UK, of whom approximately two-thirds have been diagnosed and seen for HIV-related care. The annual number of new HIV diagnoses made in the UK continues to rise, with 7450 new cases in 2005 (1), and as more people receive effective treatment, the number of deaths continues to fall. Thus the number of people receiving treatment and the already substantial burden on the NHS will continue for the foreseeable future.

A range of drugs are available that are active in blocking the replication of HIV. The current standard-of-care is the use of combination ART, usually consisting of 3 drugs: a backbone of 2 NRTIs with either a NNRTI or a PI. Such combination therapy has been shown to be effective in reducing viral load, preventing decline in immune function and dramatically decreases the risk of opportunistic infections and morbidity and mortality from HIV.

2.2 Managing HIV as a long-term chronic disease: changes in the treatment paradigm

The SMART study showed inferior outcomes with treatment interruption rather than continued ART, clearly demonstrating that ART needs to be continued indefinitely once it has been started (2).

Inference from the SMART data as well as consideration of cohort data has lead to a renewed debate on the possible merit of initiating ART even earlier in the course of disease than is currently the case (3). A major HIV therapeutic trial will soon commence to address the question of whether ART should be started at CD4+ counts above 500 cells/mm^3.

Consequent on the move towards extending therapy exposure (start earlier and continue indefinitely) is the realisation that patients are now facing the prospect of taking ART for decades. Considerations of maximising long-term durability, preserving a viable sequence of future drug options, and minimising long-term side effects are becoming increasingly important.

Much of the current pharmaceutical company driven research as well as investigator-initiated research is now designed to look at switching drugs or comparing regimens for toxicity or tolerability advantages. The search for cost-effective approaches to care, including ways of containing drug costs as well as looking at approaches to simplify monitoring and follow-up are also an important focus of current research. Several promising new drugs that act on different targets will soon be available (an integrase inhibitor and a CCR5 inhibitor) as well as better options of new drugs with potentially greater efficacy or reduced toxicity within existing classes. These new drugs will increase treatment options available, but will also lead to re-examination of the paradigm of care (treatment with triple-therapy, comprising 2 NRTIs and a NNRTI or PI) that has changed little in the last decade. The availability of more treatment options may also allow the flexibility to accept a small risk of treatment failure in a regimen that has fewer side effects or that effectively preserves long-term treatment options in the vast majority.

Thus research into more innovative uses of current drugs, as well as ways of combining or sequencing drugs to maximise long-term outcomes (preserving viable treatment options, minimising toxicity, minimising cost) will become increasingly important. Given that

treatment interruption is not a sensible option, (2) treatment simplification studies form an increasingly important aspect of the HIV treatment research agenda. The most promising candidates for treatment simplification are undoubtedly the PIs.

2.3 Previous trials of PI monotherapy

Four randomised controlled trials of PI monotherapy were presented as abstracts at the 16th World AIDS Conference in Toronto in August 2006, two of which have been recently published in full in a peer-reviewed journal. Three of these trials investigated switching to lopinavir monotherapy after patients had gained full virological suppression using triple-therapy. The largest of these studies, OK 04, enrolled 205 patients who were stable on triple-therapy and randomised them to switch to triple-therapy with lopinavir, or lopinavir monotherapy. Monotherapy was found to be non-inferior (defined with a non-inferiority margin of 12%) to triple-therapy at 48 weeks based on proportion with VL >500 copies/ml. The majority of patients (89%) maintained viral suppression without the need for re-induction and only two patients in this study developed primary PI mutations (4). The data for 96 weeks of follow-up were presented at the European AIDS Conference in October 2007, and showed that lopinavir monotherapy continued to be non-inferior to continuing triple-therapy, with no further virological rebounds with PI resistance. The percentage of patients without therapeutic failure was 87% in the monotherapy arm and 78% in the triple-therapy arm, and there were significantly fewer adverse events in the monotherapy arm (5). In the second study (M03-613) presented in Toronto, 155 treatment-naïve patients were randomised to induction with 2 NRTIs plus lopinavir, followed after 6 months by lopinavir monotherapy versus standard-of-care 2 NRTIs and a NNRTI. There was no significant difference in the rate of maintaining VL <50 copies/ml in the two groups through 96 weeks. Although intermittent increases in VL were seen often in the monotherapy arm, most returned to <50 copies/ml spontaneously and only 4 patients required re-introduction of NRTIs. Two patients treated in the monotherapy arm developed PI resistance mutations (6). The third study, KalMo, randomised 60 patients on stable combination therapy and with undetectable VL to continue existing ART or to receive lopinavir monotherapy. There was no difference in the proportion of patients who maintained undetectable VL in the two groups at 48 weeks (83% in standard-of-care and 86% in monotherapy), and none of the patients in the PI monotherapy arm developed resistance mutations (7). Taken together these 3 randomised controlled trials suggest that switching to lopinavir monotherapy is a viable treatment option in patients who have been already established on stable triple-therapy.

In contrast, the fourth study compared lopinavir monotherapy with triple-therapy in treatment naïve patients starting therapy for the first time and found a high proportion of patients had low level viraemia in the monotherapy arm and the on treatment analysis showed an inferior response in the monotherapy arm (8). Based on this limited data, it appears as though PI monotherapy may be more valuable when used as a switch strategy rather than as first-line therapy in treatment naïve individuals.

Although the randomised controlled trials to date have all been performed with lopinavir, other PIs may be effective in maintaining viral suppression when used as monotherapy. In a non-randomised trial, ACTG 5201, 33 patients who had achieved undetectable VL on triple-therapy with boosted atazanavir, discontinued nucleosides and remained on atazanavir monotherapy. At week 24, 3 patients had virological rebound, one of whom re-suppressed spontaneously on monotherapy. The other 2 patients had undetectable blood atazanavir levels and were presumably non-adherent to treatment. None of the patients developed resistance mutations to atazanavir (9). In a non-randomised trial conducted in Sweden, 5 of 15 patients who switched from stable triple-therapy (either an NNRTI-based or triple NRTI regimen) to boosted atazanavir monotherapy had VL rebound between weeks 12 to 16 (2 samples above 20 copies/ml) (10). The high proportion of patients who experienced rebound

may perhaps be explained by residual CYP3A4 enzyme-inducing effects of NNRTI reducing atazanavir levels in the period immediately following the switch, which might be avoided by continuing the NRTIs for the first few weeks (11). The study has also been criticised because two of the patients that failed used acid-suppressing drugs (ranitidine and lansoprazole) which are known to decrease atazanavir levels and were specifically contraindicated in the protocol (11). Of note, none of the 5 patients with VL rebound developed PI resistance, and all re-suppressed VL with re-introduction of triple-therapy (10).

There are currently two ongoing randomised controlled trials and one ongoing uncontrolled trial investigating PI monotherapy for patients who have achieved undetectable VL on existing treatment. The highly-active ART (HAART) followed by maintenance with monotherapy - Kaletra study (MAIMOKA study; ISRCTN45284754) is comparing lopinavir monotherapy to standard-of-care triple-therapy for patients who have achieved undetectable VL on treatment. The recruitment target is 240 patients with an anticipated study end date of October 2008. The primary endpoint is therapy failure (defined as VL >400 copies/ml) at 96 weeks. A phase III study of darunavir monotherapy is currently underway, and will randomise 250 patients with undetectable VL on triple-therapy to take darunavir alone, or take triple-therapy including darunavir (NCT005 13513). The primary endpoint is maintained suppression of VL <50 copies/ml at 48 weeks. The study is designed as a non-inferiority study (12% margin), and will report at end 2008/ early 2009 (12).

The Only REYataz (OREY) study (NCT00337467) is an uncontrolled study of atazanavir monotherapy in 62 patients who have achieved undetectable VL on their previous combination therapy. The primary endpoint is the proportion of patients who have virological rebound at Week 48.

2.4 Rationale and objectives of this trial

2.4.1 Why is this long-term strategy trial needed?

Although the trials of PI monotherapy described above are encouraging, this research is driven by the pharmaceutical industry with a typical short-term focus. A definitive trial focused on long-term outcomes is lacking. This trial is different in a number of respects from the previously completed or ongoing pharma-sponsored studies:

(i) Whereas other studies examine the effect of PI monotherapy *per se*, this trial will examine the effect of a *strategy* that includes prompt switch back to standard-of-care when PI monotherapy does not maintain full virological suppression of <50 copies/ml.

(ii) Whereas previous or ongoing studies are focused on specific PI drugs and specified comparator regimens, this trial allows drug selection according to patient/ physician preference and selection or switching of PIs for maximising tolerability; it is therefore more relevant to clinical practice.

(iii) Whereas other studies are of relatively short duration of one to two years, this trial has relatively long-term follow-up (up to 5 years) which is important for assessing long-term consequences of this strategy.

(iv) Whereas other studies focus on short-term VL endpoints (reflecting their commercial origin and single drug focus), this trial has an endpoint of clinical drug resistance, chosen to be most relevant to the long-term goal of maintaining effective treatment regimens.

(v) This trial is of sufficient size and, therefore, power to answer questions reliably.

(vi) This trial will collect relevant health care utilisation data for determining cost implications.

It is very unlikely that there will be a pharma-initiated study looking at long-term use of this strategy, and the trial we propose is likely to be the definitive long-term randomised controlled trial that addresses this important strategic option for long-term HIV therapy.

The trial objectives are:

1. To determine whether a strategy of switching to PI monotherapy is non-inferior to continuing triple drug therapy (the standard-of-care) in terms of the proportion of patients who maintain all their available drug treatment options after at least 3 years of follow-up.

2. To compare the safety and toxicity of PI monotherapy with standard-of-care triple-therapy over 3 to 5 years.

3. To assess the health economic benefits of use of PI monotherapy.

2.5 Risks and benefits of the PI monotherapy strategy used in this trial

2.5.1 Risk of side effects from treatment

The patients recruited into this trial will be, by definition, stable on their current standard-of care treatment regimen of 2 NRTIs and an NNRTI or PI. Patients who are taking this regimen will, in general, be free from major side effects on this regimen, as they would have otherwise changed therapy prior to enrolment. Patients who are randomised to the intervention arm will in some cases start a new PI to which they have not been exposed previously. Although the PIs that are in contemporary use are in general well tolerated, there is a risk that some patients will experience new side effects from the selected PI. Side effects of PIs include gastro-intestinal disturbances (including diarrhoea, nausea, vomiting, abdominal pain, flatulence), anorexia, hepatic dysfunction, pancreatitis; blood disorders including anaemia, neutropenia, and thrombocytopenia; sleep disturbances, fatigue, headache, dizziness, paraesthesia, myalgia, myositis, rhabdomyolysis; taste disturbances; rash, pruritus, Stevens-Johnson syndrome, hypersensitivity reactions including anaphylaxis, metabolic disturbances such as hyperglycaemia, hypertriglycerideaemia and hypercholesterolaemia and visceral fat accumulation.

A recent epidemiological study identified an association between treatment with a PI and the risk of myocardial infarction, although the effect appears modest (relative risk per year of exposure 1.16) especially when compared to the risks associated with other cardiovascular risk factors (increasing age 1.39; male sex 1.91; current smoking 2.83; history of cardiovascular disease 4.3) (13) (14). The association between PI treatment and risk of myocardial infarction was further reduced by adjusting for serum lipid levels. The overall incidence of myocardial infarction in patients exposed to PI treatment for more than 6 years was only 0.6% per year, which is small. To address this issue, this trial will include formal assessment of cardiovascular risk as part of the screening criteria, and will not enroll patients with very high level of background risk. Lipid levels will be measured periodically, and clinicians will be encouraged to manage lipid elevations fastidiously according to current BHIVA treatment guidelines. The protocol also allows switching to other protease inhibitors, some of which have minimal effects on lipid levels. Other modifiable risk factors (e.g. high blood pressure and diabetes) will also be sought actively and managed according to

guidelines, and patients who smoke will be encouraged to stop. With these measures, any increased risk of myocardial infarction in those patients who switch to a protease inhibitor for the first time is expected to be very low.

The PIs used in the trial will all be licensed drugs which have been used in many thousands of patients with HIV disease. Therefore risk of adverse events is quantifiable and known to be relatively small. Furthermore, the inclusion and exclusion criteria identify patients at risk of metabolic problems and more serious side effects and such patients will not be enrolled into the trial. Physicians and patients will be allowed to select the PI best suited to that patient, and, if necessary, to switch to an alternative PI if there are tolerability or toxicity problems. Patients will be monitored carefully during the intervention for known and unknown side effects of PIs allowing effective and appropriate management of these effects. Lastly, the protocol allows switch back to triple therapy (with or without a PI) in the event of unmanageable toxicity occurring in the PI monotherapy arm.

2.5.2 Risk of virological failure with resistance

There is a risk of virological failure with development of resistance. Patients selected for participation in this trial will be stable on their current standard-of-care regimen, with a relatively low probability of treatment failure in the long-term. Patients who are randomised to the intervention arm will have standard-of-care triple-therapy changed to PI monotherapy. Based on studies to date, it is likely that a small proportion of patients will not maintain full viral suppression on PI monotherapy, and if not addressed, there is the risk that ongoing viral replication may lead to the development of resistance mutations. In order to minimise the risk of developing resistance, patients who are known to have resistance to PIs or who have any evidence of failure on a PI-containing regimen will not be included. Most patients in the UK are now tested for resistance prior to starting therapy, but in those who have not been tested the risk of harbouring transmitted PI resistance is low (1.8% in 2005, data obtained from the UK HIV Drug Resistance Database) and so it is not a major concern if a baseline test was not done. Thus patients in the intervention arm should enjoy the full activity of the PI monotherapy. The fact that the trial is recruiting patients with stable undetectable VL (as opposed to initiating patients on PI monotherapy) means that patients will not be exposed to high levels of viral replication when they start the PI (hence the risk of developing *de novo* resistance is very low). Furthermore, patients will be monitored closely in the initial three months after treatment switch with regular VL testing, and patients who do not maintain virological suppression will be switched back promptly to triple-therapy. The protocol also includes early therapeutic drug monitoring as an additional safety measure to identify patients who have inadequate drug levels to maintain viral control. Given these conditions, it is very unlikely that patients will develop significant resistance to the PI even if they experience a short period of low level viral replication between the protocol-mandated testing points.

There is a small risk that patients who do not successfully maintain virological suppression on a regimen of PI monotherapy may fail to re-suppress even if standard-of-care triple-therapy is re-introduced. However, if re-induction is required it will likely be from a starting point of relatively low level of viral replication on PI monotherapy, and the re-introduction of standard-of-care triple-therapy in this situation is unlikely to be associated with significant risk of new mutations developing in the triple-therapy regimen. The previous PI monotherapy studies described above all report successful re-induction in patients who are not fully suppressed with PI monotherapy. The largest of these studies, OK 04, that adopted a less stringent protocol for switching back to triple-therapy than the one used in this study, found no excess resistance developing in the PI monotherapy arm (15).

In the studies of Kaletra monotherapy reported to date, virological failure has been almost entirely related to poor adherence. We anticipate that there will not be major problems with adherence in this trial. The trial will recruit HIV-infected patients who have been established

on ART for at least 6 months and will, in most cases, have been taking therapy for many years. Such patients have been repeatedly counselled about the importance of achieving and maintaining a high level of adherence to therapy, since this is known to be essential for successful HIV treatment. We will be recruiting patients who have had consistently undetectable VL measurements, and thereby selecting patients who have demonstrated an ability to sustain high levels of treatment adherence.

2.5.3 Risk of viral rebound in the genital compartment and increased HIV transmission

Whereas NRTIs are concentrated in genital secretions, and NNRTIs reach approximately the same concentration as in plasma, the concentration of PIs in genital secretions is variable and not fully known for the newer PIs (16). There is therefore a risk of sub-optimal drug levels, ongoing viral replication and development of resistance in the genital tract with PI monotherapy, even though plasma VL may be rendered undetectable. For the minority of individuals who are not practising safe sex, there will also be a theoretical risk of transmitting the resistant virus to others.

2.5.4 Risk of viral rebound in the central nervous system and neurocognitive decline

The penetration of PIs into the central nervous system (CNS) is variable and generally inferior to that of the other main drug classes. However, it appears that control of plasma viral replication is usually sufficient to control viral replication in the CNS. Furthermore, treatment with drug regimens that have limited CNS penetration does not appear to be associated with poor neurocognitive performance (17). One small study that measured VL in the cerebrospinal fluid in patients taking PI monotherapy (boosted atazanavir) found that 3 out of 20 patients had elevated VL in CSF despite VL suppression in plasma (18). This raises the theoretical concern that monotherapy (with some or all of the PIs) may lead to decline in neurocognitive function in a proportion of patients over time, although this has not been reported in any of the trials conducted to date. In this trial we will exclude patients who have evidence of significant neurocognitive impairment at screening, and neurocognitive function will be monitored throughout the study. If a patient shows evidence of decline in neurocognitive function they will be able to switch back promptly to triple therapy.

2.5.5 Benefits for trial participants and society

The benefits for trial participants who are randomised to take PI monotherapy are that they may have a reduced risk of long-term toxicity by using PI monotherapy (due to cessation of NRTIs and, in some cases, NNRTIs), a possible reduced risk of long-term failure because of the high genetic barrier to resistance (compared with NRTIs and NNRTIs), and a better profile of preserved drug options for the future. This counterbalances the small risk from new PI toxicity or development of resistance during viral load rebound as described above. All participants may benefit from the rigorous standard-of-care and increased attention to detail that are associated with participation in a clinical trial, and may appreciate some of the additional clinical measurements that are conducted as part of the trial (e.g. neurocognitive and quality of life assessments).

The benefits of the trial for society are that it may demonstrate the effectiveness of using PI monotherapy as treatment strategy, thereby increasing the number of strategies available for the long-term management of this chronic disease. This will represent an improvement in the care of HIV infected individuals. The trial may also identify a more economical approach to therapy and, in a setting of a healthcare system with finite resources; this would in turn benefit society by freeing up resources that could be deployed to other aspects of HIV care, or to other disease areas.

3. SELECTION OF CENTRES & CLINICIANS

The trial will be conducted at approximately 40 sites in the UK and 4 international sites. A list of the selected UK sites is shown in Appendix 4.

3.1 Criteria for selection of trial sites & clinicians

- The clinical trial site is involved in the treatment of HIV patients.
- The site has the potential to recruit at least 10 patients within the 12-18 month recruitment period.
- The investigator has appropriate experience of conducting trials according to Good Clinical Practice.
- Clinical trial staff are familiar with the appropriate use of investigational products, as described in the protocol.
- The clinical trial site has an adequate number of qualified staff and adequate facilities, for the foreseen duration of the trial, to conduct the trial properly and safely.

3.2 Site responsibilities

- The site must have a signed written agreement between the Trust and the MRC that will outline details of the trial governance, obligations of parties, liabilities and indemnity.
- The site must conduct the trial in accordance with the current protocol and changes will only be made when necessary to protect the safety, rights and welfare of patients.
- The site must conduct the trial in compliance with the International Conference on Harmonisation (ICH) GCP and applicable regulatory requirements.
- The site must ensure that all staff assisting with the trial are adequately informed about the protocol and the investigational products and aware of their trial related duties.
- The site must permit monitoring and audit of source documentation. Direct access to all trial related sites documents, reports and data must be available.
- The site must maintain a trial master file, which contains all essential documents for the conduct of the trial.
- The site must submit all trial data in a timely manner, and as described in the protocol.
- The site must submit promptly all serious adverse events reports and follow-up with detailed written reports as appropriate.

- The site must not disclose any trial related data without the approval of the Trial Steering Committee.

- The site must retain all trial-related documents for 15 years after the completion of the trial.

3.3 Site approval

It is expected the site will submit the trial documentation for local research and development approval promptly, and at the latest 6 weeks following ethics approval. The site should begin trial-related screening activities no later than 4 weeks following the receipt of all necessary trial approvals and documentation.

A site initiation meeting with the MRC must occur before a site can be approved to randomise patients. The following documentation should be forwarded to the MRC CTU:

- Local research ethics committee approval of the protocol, patient information sheet and informed consent form, together with translations (if required).

- CV for Principal Investigator and co-investigators.

- Delegation of Authority log.

- Copy of local pharmacy dispensing SOP.

- Approval of the institution’s local Research and Development office, if required.

For each clinical trial site, the responsibilities and contact details (phone, fax and email address) of each person working on the trial must be documented on the Delegation of Authority log. Clinical trial sites must notify the MRC CTU of any subsequent changes to trial personnel and/or their responsibilities. A current copy of the log must be stored in the trial master file at the clinical trial site and also at the MRC CTU.

4. SELECTION OF PATIENTS

Patients will be considered eligible for enrolment in the trial if they fulfil all of the inclusion criteria and none of the exclusion criteria defined below.

4.1 Patient inclusion criteria

1. Documented HIV infection on ELISA and confirmatory test.

2. Male or female patients, aged 18 years or more.

3. Receiving combination ART for at least 24 weeks with a regimen comprising 2 NRTIs and either an NNRTI or a PI (boosted or un-boosted).

4. No change in ART drugs in the 12 weeks prior to screening.

5. Plasma VL <50 copies/ml at screening and for at least 24 weeks prior to screening (must have at least 1 documented result <50 copies/ml at more than 24 weeks prior to screening, and at least 1 documented result <50 copies/ml taken within 12 weeks prior to screening). A patient who has had one VL "blip" to <200 copies/ml in the 24 weeks prior to screening may be included, provided that the 2 VL tests that immediately preceded the blip and the 2 VL tests that immediately followed the blip all gave results <50 copies/ml.

6. CD4+ count >100 cells/mm^3 at screening.
   This criterion is included because immune reconstitution is a priority in patients with very low CD4+ counts, and there is currently insufficient data to assess whether PI monotherapy will lead to equivalent rates of CD4+ recovery as standard-of-care treatment.

7. Willing to continue unchanged or to modify, antiretroviral therapy, in accordance with the randomised assignment.

8. Likely to be resident in the UK for the full duration of the trial and willing to comply with trial visit schedule throughout the follow-up period.

9. Willing to provide written informed consent.

4.2 Patient exclusion criteria

1. Known major protease resistance mutation(s) documented on prior resistance testing if performed (prior resistance testing is not mandatory for trial participation).

2. Previous change in ART drug regimen for reasons of unsatisfactory virological response (patients who have changed regimen for prevention or management of toxicity or to improve regimen convenience are permitted to enter the trial).

3. Previous allergic reaction to a PI.

4. Patient currently using or likely to require use of concomitant medication with known interaction with PI s including rifampicin, amiodarone, flecainide, bupropion, clozapine,

ergotamine, mexilitine, midazolam, pethidine, pimozide, quinidine, sertindole, sildenafil, voriconazole, zolpidem, St John's Wort.

5. Patient requiring treatment with radiotherapy, cytotoxic chemotherapy, or is anticipated to need these during the trial period.

6. Treatment for acute opportunistic infection within 3 months prior to trial screening.

7. Pregnant or trying to become pregnant at the time of trial entry.

8. History of active substance abuse or psychiatric illness that, in the opinion of the investigator, would preclude compliance with the protocol, dosing schedule or assessments.

9. History of HIV encephalopathy with current deficit >1 in any domain of the Neuropsychiatric AIDS Rating Scale (see Appendix 8).

10. Past or current history of cardiovascular disease, or 10 year absolute coronary heart disease risk of >30%, or risk of >20% if the patient has diabetes or a family history of premature ischaemic heart disease or stroke (based on the Framingham equation (19), and assessed using the Joint British Societies cardiovascular risk prediction charts, Appendix 11).

11. History of insulin-dependent diabetes mellitus.

12. Patient currently receiving interferon therapy for Hepatitis C virus infection or planning to start treatment for Hepatitis C at the time of trial entry.

13. Co-infection with hepatitis B, defined as Hepatitis BsAg positive at screening or at any time since HIV diagnosis, unless the patient has had a documented Hepatitis B DNA measurement of less than 1000 copies/ml taken whilst off Hepatitis B active drugs. *This criterion is included to avoid the risk of a flare of Hepatitis B with NRTI withdrawal.*

14. Any other active clinically significant condition, or findings during screening medical history or examination, or abnormality on screening laboratory blood tests that would, in the opinion of the investigator, compromise the patient's safety or outcome in the trial.

15. Fasting plasma glucose >7.0mmol/L at trial screening.

4.3 Screening procedures and pre-randomisation investigations

4.3.1 Pre-screening check

Prior to the screening visit, a check of medical and drug history should be performed to ensure that the patient meets the basic medical eligibility criteria. Patients will be given adequate information about the trial together with a Patient Information Sheet (see Appendix 1) and given an opportunity to ask questions about the trial.

4.3.2 Screening visit

Potentially eligible individuals can be screened between 1 and 4 weeks before trial entry (i.e. Week -4 to Day 0) but results from the screening must be available prior to randomisation. The screening visit will be scheduled to occur in the morning due to the need for fasting blood tests. Patients should be instructed to not eat or drink anything (except plain water) from midnight before the visit, although they may take any necessary routine medication. In the event that the patient has not fasted at the screening visit, the glucose and cholesterol

measurement will be omitted and they will be asked to return within 1 week in the fasted state for these tests to be performed.

At the screening visit patients will be asked to verify that they have read the Patient Information Sheet and will be given a further opportunity to ask questions about the trial. If the patient is willing to proceed, the patient and Investigator must both sign 3 copies of the main trial Consent Form (see Appendix 2) (one copy to be given to the patient, one copy for the patient's clinic notes and one copy for the trial file) before any trial-specific screening procedures are carried out. All individuals screened must have their name, date of birth and clinic number recorded in the Trial Register against the next available trial number. This will then become their allocated trial number. The Trial Register must be stored by the investigator in a secure place only accessible to appropriate clinic staff. If an individual is not subsequently randomised the reason should be recorded in the register.

Screening assessments will be performed as listed below and as summarised in the trial schedule (see section 1.3):

- Review of medical history (including previous and current clinically important diseases and medications) and recording demographic information
- Review of symptoms
- Physical examination (including measurement of blood pressure)
- Assessment of cardiovascular risk factors and estimation of 10 year absolute coronary heart disease risk using the Joint British Societies cardiovascular risk prediction charts (see Appendix 11) (19).
- Resting 12-lead electrocardiogram (ECG). The results of an ECG performed during the previous 6 months will be acceptable, provided that the printout is filed and available for review in the patients' case record. The ECG may be performed on or after the day of screening, but results must be available at the randomisation visit which needs to occur within 4 weeks following the screening visit.
- Laboratory tests:
  - HIV viral load
  - CD4+ count
  - Full blood count (haemoglobin, white cells, neutrophils, lymphocytes and platelets)
  - Biochemistry (sodium, potassium, urea, creatinine, bilirubin, ALT, alkaline phosphatase)
  - Bone profile (calcium, phosphate)
  - Fasting glucose
  - Fasting lipids (total cholesterol, triglycerides, LDL, HDL)
  - Hepatitis B surface antigen test and hepatitis C antibody test. The results of tests performed during the previous 6 months will be acceptable
  - Plasma storage (2 X 4ml EDTA tube)
  - Urine protein / creatinine ratio
  - Urine albumin / creatinine ratio and phosphate
  - Urine storage
  - Urine (or serum) pregnancy test for women of childbearing potential

5. RANDOMISATION & ENROLMENT

5.1 Randomisation visit (Baseline; Day 0)

The randomisation visit should be scheduled for 1-4 weeks following the screening visit, but the results of all the screening assessments (including ECG) must be available prior to randomisation. There is no requirement for fasting at this trial visit.

For trial entry, all eligibility criteria must be fulfilled. These include the results of the evaluations carried out at screening which must be reviewed prior to the randomisation to make sure that the patient meets the criteria for trial entry. The research team should confirm that the patient continues to consent to enter the trial. The patient will be advised to inform their general practitioner of their trial participation, but this will not be a pre-requisite to enrolment. If the patient consents for their GP to be informed about participation (see Appendix 2) a letter should be sent to the GP (see Appendix 3).

Baseline assessments will be performed as listed below and as summarised in the Trial Schedule (see section 1.3).

- Review of medical and drug history since the screening visit
- Review of symptoms
- Review of concomitant medications
- Adherence to ART (patient will be asked about the number of missed doses in the previous 2 weeks, and in the previous 3 months)
- Targeted physical examination (as needed to evaluate symptoms; at this visit will include measurement of blood pressure, body weight, waist and hip-circumference, assessment of facial lipoatrophy and peripheral neuropathy)
- Neurocognitive assessment
- MOS-HIV quality of life questionnaire (self-completed by patient)
- EQ-5D health status questionnaire (self-completed by patient)
- Laboratory tests:
    - HIV viral load
    - CD4+ count
    - Full blood count (haemoglobin, white cells, neutrophils, lymphocytes and platelets)
    - Biochemistry (sodium, potassium, urea, creatinine, bilirubin, ALT, alkaline phosphatase)
    - Plasma storage (1 X 4ml EDTA tube)

5.2 Procedure for randomisation

To randomise a patient the completed randomisation pages of the CRF must be faxed to the MRC CTU while the patient is present in the clinic.

RANDOMISATION

Tel: +44(0)20 7670 4843 (Mon - Fri, 09:00 – 17:00)
Fax: +44(0)20 7670 4817

Eligibility will be checked and if there are queries regarding the CRF the MRC CTU will contact the site by phone. Randomisation will be performed using a computer-generated randomisation list and the details of the patient's treatment allocation will be notified to the trial team at the site by fax and by phone within one hour of the receipt of the randomisation form. The patient's trial number, treatment allocation and the date of randomisation will be entered into the Trial Register at the MRC CTU, and these details should also be entered into the Trial Register at the site.

5.3 Post-randomisation procedures and follow-up

The clinician will complete a prescription with the patient's details and trial medication as allocated. The prescription will be for 3 months, or until the next protocol-mandated trial visit (see section 6.4).

Trial visit schedules will be sent by email or fax to the site at the time of randomisation. Patients should be followed on the same schedule even if their trial medication is discontinued. The target dates for trial visits are determined by the date of randomisation and are not affected by subsequent events. The schedule defines visit dates (with windows) necessary for data collection, but the patient may be seen more frequently for clinical care as needed.

Patients will also be given a card with the contact details for the trial research team.

Patients in the PI monotherapy arm will be asked to return in 4 weeks (visit window ±2 weeks). The Week 4 visit should be scheduled for the morning, and patients should be instructed not eat or drink anything (except plain water) from midnight before the visit. They should also be instructed to omit any morning dose of PI medication, and bring the medication with them to the clinic. They may take any other necessary dose of routine medication as usual.

Patients randomised to continue triple-therapy will be asked to return in 12 weeks. The Week 12 visit should be scheduled for the morning, and they should be instructed not eat or drink anything (except plain water) from midnight before the visit. They may take all their medication as usual.

5.4 Co-enrolment guidelines

Patients may not participate in any other clinical intervention trial while on this trial. Participation in other trials that do not involve an intervention may be permitted, but this should be discussed first with the Chief Investigator of this trial.

6. TREATMENT OF PATIENTS

6.1 Introduction

Patients will be randomised to a strategy of switching to a regimen comprising a single ritonavir-boosted PI or continuing triple drug therapy.

6.2 Control arm

6.2.1 Choice of triple therapy drugs

Patients randomised to the control arm will continue to take their standard-of-care triple-therapy regimen. Changes of therapy can be made for virological failure or drug-related toxicity as clinically indicated, but patients will be expected to remain on the strategy of receiving standard-of-care triple-therapy for the duration of the trial. The choice of drugs for use in the triple-therapy strategy is left to the discretion of the physician and patient. In the event that the patient develops drug resistance to all three standard classes of drugs, the clinician may select any combination of drugs that is considered clinically appropriate and this need not be limited to standard-of-care triple therapy.

Any change in therapy should be followed by a viral load at 4 weeks to verify that undetectable viral load is maintained.

6.2.2 Virolological monitoring and switching strategy

Patients in the triple therapy arm will have VL testing performed every 12 weeks during the study (see trial schedule, section 1.3). In the event of VL rebound greater than 50 copies/ml (confirmed on a second laboratory run, as described in section 7.2.1), the patient will be contacted by telephone, counselled on medication adherence, and asked to return for a repeat blood test within 4-6 weeks from the date of the first test. At this next visit, blood will be drawn for a repeat VL test, for resistance testing (saved and performed only if VL rebound confirmed) and for PIVOT blood storage (2 X 4ml plasma samples). The assessments performed at this visit are described in section 7.1.5.

Patients who develop confirmed virological rebound (defined as 2 consecutive VL values above 50 copies/ml taken 4-6 weeks apart, with the first being confirmed by a repeat assay on the first sample) whilst taking triple therapy will ordinarily be expected to change therapy to a new optimised regimen, taking into account the results any resistance tests performed. However the clinician and /or patient may elect to continue the current regimen without change if considered appropriate, and the reasons for not changing therapy will be documented on the case report form. A further VL must be obtained within 4 weeks (together with an additional 2 X 4ml sample for plasma storage) and if this remains detectable patients should change therapy.

If the clinician and/or patient choose to continue the triple therapy regimen, the reasons for not changing therapy will again be documented on the case report form. VL testing will be repeated at least as often as every 12 weeks (more often if clinically warranted). A blood sample for resistance testing and PIVOT plasma sample storage (1 X 4ml) will also be repeated every 12 weeks until VL suppression occurs spontaneously or treatment is changed. At the time of changing therapy a repeat sample will be sent for resistance testing and a PIVOT plasma sample will be taken (1 X 4ml) if these have not been performed in the 4-weeks prior to switching therapy.

6.3 PI monotherapy arm

6.3.1 Commencing PI monotherapy

Patients who are receiving an NNRTI-based regimen at baseline will discontinue the NNRTI and start a single ritonavir-boosted PI immediately following the randomisation visit. The two NRTIs will continue for a further 2 weeks after randomisation and then stop, so that from that point the patient will take only PI monotherapy. Consideration should be given to prescribing anti-diarrhoeal medication (e.g. loperamide) for the initial period of following PI initiation, if considered clinically appropriate to do so.

Patients who are receiving a PI-based regimen at baseline will discontinue the two NRTIs immediately following the randomisation visit. They will continue on a single ritonavir-boosted PI only, which may be the same or a different PI from the one they were taking at baseline.

Patients will be reminded of the importance of maintaining excellent adherence when they switch to PI monotherapy. They will be instructed that, in the event of experiencing new side effects or for other reasons, they should not interrupt or change medication themselves but rather should seek medical advice from the study team so that changes or interruption in medication can be managed appropriately

6.3.2 Choice of protease inhibitor

This is a strategic trial and hence the choice of protease inhibitor will be left to physician and patient discretion. Any licensed, ritonavir-boosted PI will be permitted. Switches to alternative PIs will be permitted during the trial to avoid or minimise drug-related toxicity, to minimise the risk of interactions with any necessary concomitant medication, to create a more acceptable treatment schedule, or to take account of changes in current opinion of the relative merits of protease inhibitors in this therapeutic setting.

The trial team at the MRC CTU will endeavour to provide investigators with relevant information, updates and any data from other trials of PI monotherapy that enters the public domain during the course of this trial so that investigators may use these to guide their choice of protease inhibitor monotherapy.

Any change in PI should be followed by a viral load at 4 weeks to verify that undetectable viral load is maintained. The PI drug concentration will also be measured to ensure that therapeutic drug levels are attained with the new PI.

6.3.3 Dose adjustment using therapeutic drug monitoring

A blood sample will be taken at Week 4 for measurement of the concentration (as close as possible to a trough level) of the PI (see section 7.3.1), and the results will be made available to the treating clinician prior to the Week 8 trial visit. The drug levels will be compared with the concentration-based cut-off values for efficacy (C_{trough}) given in international guidelines (20):

- Atazanavir 150 ng/mL
- Fosamprenavir 400 ng/mL
- Indinavir 100 ng/mL
- Lopinavir 1000 ng/mL
- Nelfinavir 800 ng/mL
- Saquinavir 100 ng/mL
- Tipranavir 20500 ng/mL

The cut-off value for darunavir will be taken as 550 ng/mL based on protein binding corrected EC50 in-vitro (21).

If the concentration of the PI is below the specified threshold, adjustments to the dose of the PI, the boosting dose of ritonavir, or the dose frequency may be made if thought to be clinically appropriate by the treating physician. The possibility of drug interaction with any concomitant medication the patient may be taking should also be considered prior to dose adjustment (see section 6.6). Expert advice will be available to the treating physician from the trial research team that includes an experienced clinical pharmacologist. All requests for such advice will be directed through the MRC CTU. If the PI concentration is below the stated threshold at week 4, up to 2 further therapeutic drug monitoring assays may be performed up to week 12, either to re-check a borderline low concentration, or to re-check the concentration after dose adjustment.

6.3.4 Virological monitoring and switching strategy

Patients in the PI monotherapy strategy arm will have VL testing performed at Weeks 4, 8 and 12, and every 12 weeks thereafter (see trial schedule, section 1.3). In the event of VL rebound greater than 50 copies/ml (confirmed on a second laboratory run, as described in section 7.2.1), the patient will be contacted by telephone, counselled on medication adherence, and asked to return for a repeat blood test within 4-6 weeks from the date of the first test. At this next visit, blood will be drawn for a repeat VL test, for resistance testing (saved and performed only if VL rebound confirmed) and for PIVOT blood storage (2 X 4ml plasma samples). The assessments performed at this visit are described in section 7.1.5.

Patients who develop confirmed virological rebound after switching to PI monotherapy (defined as 2 consecutive VL values above 50 copies/ml taken 4-6 weeks apart, with the first being confirmed by a repeat assay on the same sample) should switch promptly back to a triple-therapy regimen. Exceptionally, upon receiving the second detectable VL result investigators may choose to switch to an alternative PI drug or dosing schedule if they consider that there is a strong chance that VL control could be re-established rapidly (e.g. by switching from once daily to twice daily PI). The reasons will be documented on the case report form. A further VL must be obtained within 4 weeks (together with an additional 2 X 4ml plasma sample for storage). If the VL remains detectable, patients should switch back to triple-therapy immediately.

If the clinician and /or patient choose to continue the PI monotherapy regimen, the reasons for not restarting triple therapy will again be documented on the case report form. This should also be discussed with the chief investigator at MRC CTU. If the PI monotherapy is maintained, VL testing will be repeated at least as often as every 12 weeks (more often if clinically warranted). A blood sample for resistance testing and PIVOT plasma sample storage (1 X 4ml) will be repeated every 12 weeks until VL suppression occurs spontaneously or treatment is changed. At the time of changing therapy a repeat sample will be sent for resistance testing and a PIVOT plasma sample will be taken (1 X 4ml) if these have not been performed in the 4-weeks prior to switching therapy.

When a patient resumes triple therapy they will be managed exactly as for patients in the control arm, as described in section 6.2.

6.3.5 Strategy for stopping or interrupting treatment with PI monotherapy

In addition to the virological rebound criteria given above, patients may be withdrawn (temporarily or permanently) from treatment with PI monotherapy for any of the following reasons at the discretion of the investigator:

- Disease progression while on therapy
- Unacceptable toxicity (e.g. insulin dependent diabetes developing *de novo*)
- Need for concomitant medication that has known interactions with the PI (and that cannot be avoided by switching to an alternative PI)

- Serious intercurrent illness or any change in the patient's condition which justifies the discontinuation or interruption of treatment in the clinician's opinion
- Pregnancy
- Patient withdraws consent

PI monotherapy should be resumed as soon as possible, if considered clinically appropriate to do so (e.g. toxicity resolves, patient is no longer pregnant, or requirement for contraindicated concomitant medication ends).

6.3.6 Strategy for switching back to triple-therapy

In the event that triple-therapy needs to be resumed, this should be the regimen that the patient was taking at trial entry. However, alternatives will be allowed if there is a strong preference for choosing alternative drugs (e.g. patients may have experienced relief from efavirenz-related neuropsychiatric side effects and may wish to continue on the PI), if there is a good clinical reason why change is indicated, or if the results of any resistance tests performed suggest that a particular combination would be preferable to that originally taken at baseline.

After returning to triple-therapy, changes of drugs can be made for subsequent virological failure or drug related toxicity as clinically indicated. Patients will be expected to remain on the strategy of receiving triple-therapy for the remaining duration of the trial, unless the interruption was temporary for one of the reasons listed in section 6.3.5. Resumption of PI monotherapy after a temporary suspension should be followed by VL testing after 4 weeks.

6.4 Management of drug toxicity and adverse events

Toxicity will be managed in both arms according to standard clinical practice. Wherever possible, any side effects will initially be managed by symptomatic measures and administration of appropriate (non-contraindicated) medication. In particular, Grade I-II gastrointestinal side effects such as nausea (with or without vomiting), and diarrhoea will be managed by anti-emetics and or antidiarrhoeal agents in the first instance. See Appendix 7 for details of toxicity grading. Interruption of or changes in ART will be avoided except in the event of Grade III or IV toxicity that is considered at least possibly related to one or more of the ART drugs. Wherever possible, alternative ART drugs will be selected that maintain the patient's randomised treatment strategy.

6.5 Management of cardiovascular disease risk factors

Cardiovascular risk factors (smoking status, blood pressure, fasting serum lipids and glucose) will be evaluated at baseline and annually during the trial.

At each of these annual assessments patients who report that they continue to smoke will be advised to give up smoking. This should be particularly emphasised in patients who have other factors that elevate cardiovascular risk. Patients who indicate that they are willing to attempt to stop smoking will be provided with contacts for smoking cessation programmes, or other counselling and assistance as appropriate.

Elevated blood pressure will be managed according to standard treatment guidelines.

Elevated lipid levels will be evaluated according to standard thresholds for treatment and managed according to current treatment guidelines. Management decisions should be based on fasted results. If elevated triglyceride or cholesterol results are obtained from a non-fasting blood sample, the test should be repeated after an overnight (minimum 8-hour) fast

before management changes are instigated. Management will initially involve counselling to make appropriate changes in diet and exercise routine. Subsequently, if indicated, anti-hyperlipidaemia medication may be used. If treatment with a statin is indicated, a drug that is not metabolised by the CYP3A4 pathway should be prescribed e.g. pravastatin (but consider possible interaction with darunavir), atorvastatin, or rosuvastatin (but consider possible interaction with lopinavir or darunavir). Up-to-date information on drug interactions should be sought (see reference sources in section 6.6, below). If these measures fail to provide adequate control of lipids, changes in ART may be considered (e.g. switching to an alternative PI or NNRTI that is considered to be less prone to cause hyperlipidaemia). Every attempt should be made to maintain the patient within the allocated treatment strategy and consideration should be given to discontinuing a PI only if lipid levels cannot be adequately controlled with these measures.

Diabetes will be diagnosed by standard approaches, and managed according to current best clinical practice. Management decisions should be based on fasted results. If elevated glucose levels are obtained from a non-fasting blood sample, the test should be repeated after an overnight (10-hour) fast before management changes are instigated. Management may involve initial referral for counselling and initiation of a diabetic diet, and prescription of oral hypoglycaemic agents. Switching to an alternative PI (or NNRTI) that is considered to be less prone to cause diabetes may be considered. Every attempt should be made to maintain the patient within the allocated treatment strategy and consideration should be given to discontinuing a PI only if the blood sugar cannot be adequately controlled with these measures. However, for patients who develop insulin dependent diabetes *de novo* following switching to a PI, it would be appropriate to consider early switching to an alternative ART regimen if available.

6.6 Concomitant medication

A large number of drugs have interactions with ART, and before prescribing any concomitant medication in this trial investigators should check with the drug interaction information listed in the current BNF (http://www.bnf.org) or current summary of product characteristics in the electronic medicines compendium (http://emc.medicines.org.uk), or with the detailed information on drug interactions provided in the University of Liverpool HIV drug interactions website (http://www.hiv-druginteractions.org).

In this trial, particular attention needs to be paid to avoid prescribing concomitant medications that are known to reduce PI drug levels, such as rifampicin, anticonvulsants, St John's Wort and acid-reducing agents. These interactions may differ between the individual PIs and the particular medications with drug classes, and details should be checked on the University of Liverpool HIV drug interactions website (http://www.hiv-druginteractions.org). A number of other medications may have serious interactions with one or more PIs, including warfarin, calcium channel blockers, ergot derivatives, benzodiazepines, and antifungals.

The latest summary checklist of PI drug interactions from the University of Liverpool HIV drug interactions website (http://www.hiv-druginteractions.org) will be sent to investigators at the start of the trial.

6.7 Drug supply, dispensing and accountability

Patients will be given a one to three month drug supply at each visit, corresponding to the protocol mandated frequency of follow-up. Prescriptions will be written on standard hospital forms, and drugs will be supplied from the hospital pharmacy. The local pharmacy

dispensing SOP will be followed, including a record of the batch numbers of antiretroviral drugs dispensed.

6.8 Adherence assessment and counselling

At each of the trial visits, patients will be asked several simple questions to assess their adherence (number of missed doses since the last trial visit, number of missed doses in previous 2 weeks). The answers will be captured on the visit CRF, and patients will be given counselling to reinforce the critical importance of adherence if their responses indicate that adherence is not optimal.

7. ASSESSMENTS & FOLLOW-UP

7.1 Routine Follow-up

The trial will run for 5 years, with recruitment being completed within the first 12-18 months. All patients will continue follow-up to a common closing date.

The assessments are listed in the trial schedule (see section 1.3). Trial visits and assessments will correspond closely to routine clinical care, with 12-weekly visits throughout the trial. Patients randomised to the PI monotherapy arm will have additional visits at Week 4 and Week 8. Additional visits for VL testing will be required at 4-6 weeks following any confirmed VL rebound (see section 7.1.6). Data will be entered on the CRF at each visit.

7.1.1 Week 4

This visit is only required for patients randomised to the ***PI monotherapy arm.***

The visit window is ±2 weeks from the target visit date. The visit will be scheduled to occur in the morning due to the requirement for fasting blood tests. Patients should be instructed to not eat or drink anything (except plain water) from midnight before the visit. They should be instructed not to take any morning dose of PI medication (other medication can be taken) until after the blood has been drawn. The following assessments will be performed:

- Review of symptoms
- Review of concomitant medications
- Adherence to ART (no. of missed doses in previous 2 weeks and since last visit)
- Targeted physical examination (as needed to evaluate reported symptoms)
- Review of healthcare resource utilisation since last visit (patient self-report and review of case sheet)
- EQ-5D health status questionnaire
- Laboratory tests:
  - HIV viral load
  - Full blood count (haemoglobin, white cells, neutrophils, lymphocytes and platelets)
  - Biochemistry (sodium, potassium, urea, creatinine, bilirubin, ALT, alkaline phosphatase)
  - Fasting glucose
  - Fasting lipids (total cholesterol, triglycerides, LDL, HDL)
  - PI drug concentration (trough level preferred, or at least 4 hours from last dose)
  - Plasma storage (1 X 4ml EDTA sample)

7.1.2 Week 8

This visit is only required for patients randomised to the ***PI monotherapy arm.***

The visit window is ±2 weeks from the target visit date. The patient should be instructed not to take any morning dose of PI medication (other medication can be taken) until after they have been assessed in the clinic, in case a repeat PI drug level concentration may be required. If the trough level result at Week 4 is known to be above the threshold, then the patient can be instructed prior to the visit that the morning PI dose can be taken as scheduled (because a repeat drug concentration level will not be needed at Week 8). Adjustment to PI dose, ritonavir dose or drug schedule may be made at this visit depending on the results of PI levels measured at Week 4 (these will be available at this visit).

The required assessments at this visit are:

- Review of symptoms
- Review of concomitant medications
- Adherence to ART
- Review of healthcare resource utilisation since last visit (patient self-report and review of case sheet)
- EQ-5D health status questionnaire
- HIV viral load

Optional:

- PI drug concentration (trough level preferred, or at least 4 hours from last dose). This is only indicated if the Week 4 drug concentration was below the threshold and the clinician thinks it appropriate to recheck the level.

7.1.3 Week 12

This visit is required for ***all patients.***

The visit window is ±2 weeks from the target visit date. The visit will be scheduled to occur in the morning due to the requirement for fasting blood tests. Patients should be instructed to not eat or drink anything (except plain water) from midnight before the visit.

Patients on PI monotherapy arm who had a low PI concentration at Week 4 and for whom the clinician has decided that a further check on drug levels is warranted (for example if a dose adjustment has been performed at week 8) should be instructed not to take any morning dose of PI medication (other meds can be taken) until after the blood has been drawn. All other patients can take their morning medication as scheduled. The following assessments will be performed:

- Review of symptoms
- Review of concomitant medications
- Adherence to ART (no. of missed doses in previous 2 weeks and since last visit)
- Targeted physical examination (as needed to evaluate reported symptoms)
- Neurocognitive assessment
- Review of healthcare resource utilisation since last visit (patient self-report and review of case sheet)
- EQ-5D health status questionnaire (self-completed by patient)
- MOS-HIV quality of life questionnaire (self-completed by patient)
- Laboratory tests:
    - HIV viral load
    - CD4+ count
    - Full blood count (haemoglobin, white cells, neutrophils, lymphocytes and platelets)
    - Biochemistry (sodium, potassium, urea, creatinine, bilirubin, ALT, alkaline phosphatase)
    - Bone profile (calcium and phosphate)
    - Fasting glucose
    - Fasting lipids (total cholesterol, triglycerides, LDL, HDL)
    - Urine protein / creatinine ratio
    - Plasma storage (1 X 4ml EDTA sample)

Optional:

- PI drug concentration (trough level preferred, or at least 4 hours from last dose). This is only indicated for patients in the PI monotherapy arm in whom the Week 4 drug concentration was below the threshold and for whom the clinician thinks it appropriate to recheck the level.

7.1.4 Subsequent 12-weekly visits (excluding "annual" visits and final trial visit)

These visits are required for ***all patients.***

The visit windows are ±6 weeks from the target visit date. There is no requirement for fasting or drug scheduling for these visits. The following assessments will be performed:

- Review of symptoms
- Review of concomitant medications
- Adherence to ART (no. of missed doses in previous 2 weeks and since last visit)
- Targeted physical examination (as needed to evaluate reported symptoms)
- Review of healthcare resource utilisation since last visit (patient self-report and review of case sheet)
- EQ-5D health status questionnaire (self-completed by patient)
- Laboratory tests:
  - HIV viral load
  - CD4+ count
  - Full blood count (haemoglobin, white cells, neutrophils, lymphocytes and platelets)
  - Biochemistry (sodium, potassium, urea, creatinine, bilirubin, ALT, alkaline phosphatase)
  - Bone profile (calcium and phosphate; only required for patients taking tenofovir)
  - Urine protein / creatinine ratio (only required for patients taking tenofovir)

7.1.5 "Annual" visits (week 48, 96, 144, 192, 240) and final trial visit

These visits are required for ***all patients.***

The visit windows are ±6 weeks from the target visit date. Patients should be instructed to not eat or drink anything (except plain water) from midnight before the visit. The following assessments will be performed:

- Review of symptoms (including specific questions about neuropathy and body fat changes)
- Review of concomitant medications
- Adherence to ART (no. of missed doses in previous 2 weeks and 3 months)
- Targeted physical examination (as needed to evaluate reported symptoms; at this visit will include measurement of blood pressure, body weight, waist and hip-circumference and assessment of facial lipoatrophy; the final trial visit will also include an assessment of peripheral neuropathy)
- 10-year cardiovascular risk assessment
- Neurocognitive assessment
- Review of healthcare resource utilisation since last visit (patient self-report and review of case sheet)
- EQ-5D health status questionnaire (self-completed by patient)
- MOS-HIV quality of life questionnaire (self-completed by patient)
- Laboratory tests:
  - HIV viral load
  - CD4+ count
  - Full blood count (haemoglobin, white cells, neutrophils, lymphocytes and platelets)
  - Biochemistry (sodium, potassium, urea, creatinine, bilirubin, ALT, alkaline phosphatase)
  - Bone profile (calcium and phosphate; required for all patients at this visit)
  - Fasting glucose
  - Fasting lipids (total cholesterol, triglycerides, LDL, HDL)

- Hepatitis B and C serology (if clinically indicated)
- Plasma storage (1x 4ml EDTA at annual visits; 2x 4ml EDTA at final trial visit)
- Urine protein / creatinine ratio (required for all patients at this visit)
- Urine albumin / creatinine ratio and phosphate
- Urine storage

7.1.6 Additional visits for virological rebound

These visits are required for ***all patients.***

In the event that the patient has a VL rebound >50 copies/ml, and this result is confirmed (by repeat laboratory testing on the same sample), an additional visit will be required and this should be scheduled to occur at 4-6 weeks following the date of the visit at which the sample with VL rebound was obtained.

Second VL rebound visit

For this repeat visit, patients in the PI monotherapy arm should be instructed not to take their morning dose of PI medication (other medications can be taken) until after the blood has been drawn. All other patients can take their morning medication as scheduled. The following assessments will be performed at this visit:

- Review of symptoms
- Review of concomitant medications
- Adherence to ART (no. of missed doses in previous 2 weeks and 3 months)
- Targeted physical examination (as needed to evaluate reported symptoms)
- Review of healthcare resource utilisation since last visit (patient self-report and review of case sheet)
- EQ-5D health status questionnaire (self-completed by patient)
- Laboratory tests:
  - HIV viral load
  - Resistance testing (store until VL test result available and only proceed if >50 copies/ml on the second sample)
  - Plasma storage (2x 4ml EDTA sample for later batched tests which may include PI drug levels)
  - Additional tests as per trial schedule or if clinical indication

If the second VL test shows an undetectable VL, follow-up will resume according to the next routine visit in the trial schedule. If the second VL test shows a VL >50 copies/ml, patients will be managed as described in section 6.2.2 and 6.3.4. Ordinarily, therapy will be changed and patients will subsequently be managed as described in section 7.1.7 below. If therapy is continued unchanged, then a third VL rebound visit will be arranged to occur within 4 weeks.

Third VL rebound visit

The same assessments listed above (for the 2nd rebound visit) will be performed.

If the third test shows undetectable VL, follow-up will resume according to the next routine visit in the trial schedule. If the third VL test shows a VL >50 copies/ml, patients will be managed as described in section 6.2.2 and 6.3.4. Ordinarily, therapy will be changed and patients will subsequently be managed as described in section 7.1.7 below. If therapy is continued unchanged, then subsequent visits will be arranged at no less often than every 12 weeks until the patient changes treatment.

Subsequent VL rebound visits

At these subsequent visits the following assessments will be performed:

- Review of symptoms
- Review of concomitant medications

- Adherence to ART (no. of missed doses in previous 2 weeks and 3 months)
- Targeted physical examination (as needed to evaluate reported symptoms)
- Review of healthcare resource utilisation since last visit (patient self-report and review of case sheet)
- EQ-5D health status questionnaire (self-completed by patient)
- Laboratory tests:
  - HIV viral load
  - Resistance testing (needed if more than 70 days have elapsed since the date of the last resistance test sample; store blood and only proceed if VL > 50 copies/ml on this sample)
  - Plasma storage (1 x 4ml EDTA sample for later batched tests which may include PI drug levels)
  - Additional tests as per trial schedule or if clinical indication

When the patient eventually changes treatment, an additional visit will be arranged as described in section 7.1.7 below.

7.1.7 Additional visits following ART change

This visit is required for ***all patients.***

For patients in either treatment arm who change therapy as a result of VL rebound, an additional visit should be scheduled at 4-6 weeks following the date of therapy change. Review of symptoms, concomitant medication and treatment adherence will be performed. The only required laboratory assessment at this visit is VL testing, but additional laboratory tests may be performed as clinically indicated. Follow-up should then resume according to trial schedule, but further unscheduled visits may be arranged if considered appropriate for clinical management.

7.2 Procedures for assessing efficacy

7.2.1 Viral load

VL testing will be performed at the local site laboratory with the standard assay for detecting low levels of VL that is routinely used at the site. The site laboratory will be required to demonstrate satisfactory participation in a recognised quality control program for VL testing.

Any VL result >50 copies/ml will be initially confirmed by re-testing the same sample on a separate laboratory run. If the result is confirmed as >50 copies/ml, the patient will be recalled for a repeat sample. This second assay need be run only once.

In order to be able to extrapolate the findings beyond the 5 year period of the trial, it will be important to document that the patients on PI monotherapy who remain virologically suppressed during the trial have full virological suppression (to the same extent as patients on triple-therapy). Therefore, additional testing using a very low copy assay (<5 copies/ml) will be performed at the central virology laboratory on the stored plasma sample from the final visit in all patients who have VL <50 copies/ml on conventional testing at the final visit.

7.2.2 CD4+ count

CD4+ counts will be measured at the local site laboratory using the standard flow cytometry method used by the site. The site laboratory will be required to demonstrate satisfactory participation in a recognised quality control programme for CD4+ count testing.

7.2.3 Resistance testing

Drug resistance is the key component of the primary endpoint, and hence the detection of new resistance mutations needs to be pursued fastidiously in this trial.

All patients who have virological rebound more than 50 copies/ml on 2 consecutive readings (taken at least 4 weeks apart) will have a sample sent for genotypic resistance testing (from the sample that gave the highest VL result, or if there is less than 50 copies/ml difference in the VL results, the later of the two samples will be chosen in preference). Resistance testing will be performed by the local laboratory that is normally used by the site. A designated referral laboratory, normally used by the site for performing resistance testing at low VL levels, may be used also. In the event that sequencing is unsuccessful, the sample will also be tested by the study central resistance laboratory at UCL/UCLH. All the sequences will be analysed using the Stanford algorithm, and the level of resistance to individual drugs will be classified as none (susceptible), potential low level, low level, intermediate level, high level.

Comparison will be made with the drug resistance profile obtained from any other previous sequences available for that patient (pre-treatment resistance testing is commonly performed at most centres in the UK, and prior resistance testing is mandated in the trial entry criteria for patients who have failed previous ART). If a patient develops virological rebound with resistance during the course of the trial but does not have a prior resistance test result for comparison, attempts will be made to identify a stored (pre-treatment) sample and if available this sample will be tested to determine whether transmitted resistance was present prior to antiretroviral therapy.

As a further measure to make the best possible comparison of the two treatment strategies in terms of drug resistance development, stored samples from patients who have rebounded (VL >50 copies/ml) will be tested using a minority species resistance assay at the central virology laboratory at the end of the trial.

7.2.4 Quality of Life (QoL) assessment

This will be assessed using the Medical Outcomes Study HIV Health Survey (MOS-HIV), a 30-item QoL questionnaire based on the SF36 which has been validated for use in patients with HIV infection (22) (see Appendix 6). The questionnaire takes about 5 minutes for patients to self-complete, and this will be done at baseline, at 12 weeks, and annually during the trial.

7.2.5 Neurocognitive assessment

Neurocognitive function will be assessed at Baseline, Week 12, annual visits and the final trial visit using a series of simple neuropsychological tests that can be administered by a clinician or research nurse without specific neuropsychological training, and that will take about 20 minutes to perform. The most common neurocognitive impairments seen in HIV-infected individuals are those that affect frontal sub-cortical functions (23-25). Therefore, the tests have been chosen to detect changes in these functions such as psychomotor speed, memory, executive functions (e.g. processing instructions) and fine motor speed. The following simple, well-established tests will be performed (details provided in Appendix 13):

- Hopkins Verbal Learning Test - Revised. This assesses verbal learning and memory. The examiner reads 12 words and the participant is asked to recall them immediately. The exercise is performed a further two times in exactly the same way. The free recall trials are followed by a recognition test where the examiner reads aloud a list of 24 words and the participant must answer yes or no for each of them depending if the word was or was not included in the original list of 12 words. Finally, after performing the remaining tests listed below and completing the MOS-HIV quality of life questionnaire (i.e. after about 15-20 minutes), the initial free recall test is repeated (without the examiner repeating the list again) to assess delayed memory. A final score based on the number of words recalled or recognised in each part of the test will be calculated (a separate score for immediate and for delayed memory will be produced).

- Colour trails tests (two parts). These assess psychomotor speed and cognitive flexibility within the executive functioning domain (26). In Part 1, participants will be asked to connect a series of encircled numbers in numerical order. In Part 2 they will be also asked to connect 25 encircled numbers alternating between two colours. The score is the time to completion of each of the tasks.

- Grooved Pegboard test. This assesses psychomotor speed and fine motor function. It is a manipulative dexterity test consisting of 25 holes with randomly positioned slots. Pegs have a key along one side and they must be rotated to match the hole before they can be inserted. Participants are asked to insert the pegs into the slots as rapidly as possible, and the test will be performed with both the dominant and the non-dominant hand. The score is the time to completion of the task (each hand scored separately).

The participant will be given their raw test scores if so desired. The investigator will be provided with a table of normal ranges for each of the tests based on standard population data so that some interpretation of the participant's score can be made, if the participant wishes results to be expressed in that way.

The final evaluation of each participant's neurocognitive function will be done centrally to calculate a neurocognitive function summary score, as described in section 9.5.2.

7.3 Procedures for assessing safety

A symptom evaluation and targeted physical examination will be performed at each visit.

Body composition changes will be evaluated at baseline, annual visits and the final study visit as follows:

- Waist circumference will be measured between the lower border of the ribs and the iliac crest, in a horizontal plane.

- Hip circumference will be measured at the widest point over the buttocks.

- Patients will be asked about any change in fat in the face, arms, legs or abdomen. Taking into account the patient's opinion, the research nurse or doctor will grade facial lipoatrophy as none, moderate (evident on inspection), or severe (immediately obvious).

Patients will be asked about symptoms of peripheral neuropathy at baseline, annual visits and the final study visit. A brief neuropathy examination will be performed at baseline and the final study visit in all patients, and at intervening annual study visits if the patient reports neuropathy symptoms

Cardiovascular risk factors will be assessed at annual trial visits, and risk will be quantified using the Joint British Societies cardiovascular risk prediction charts (see Appendix 11).

Blood will be drawn at trial visits to assess laboratory safety parameters according to standard-of-care as indicated in the schedule of trial assessments (see section 1.3). Additional safety blood tests or investigations will be performed to investigate symptoms or monitor emergent laboratory test abnormalities as clinically indicated.

All adverse events will be reported on the case report form. Adverse events (clinical and laboratory) will be graded using the 1992 Division of AIDS toxicity grading scale (see

Appendix 7). Serious adverse events will be defined according to the EU Directive 2001/20/EC Article 2 based on ICH GCP, and will be reported to the MRC CTU according to standard timelines (see section 11).

Diagnostic criteria for selected serious AIDS and non-AIDS events are defined in the protocol (see section 9.2.2 and Appendices 8 and 9), and sites will be encouraged to investigate patients in a way that allows determination of whether patients meet the specified diagnostic criteria. An independent endpoint review committee will review these endpoints to ensure that they satisfy the criteria.

7.3.1 Procedures for Therapeutic Drug Monitoring

Patients in the PI monotherapy arm will have a blood sample taken at Week 4 for measurement of PI concentration. The sample should be a trough sample (i.e. taken at the time when the next dose of medication would ordinarily be due) if at all possible, or obtained at the very least 4 hours following the last dose. The time of the last dose of PI taken will be recorded. If the PI concentration is low at week 4, then the test may be repeated at week 8 and/or week 12, at the clinician's discretion. A blood sample for PI concentration will also be taken at 4 weeks after any change in PI drug in patients on PI monotherapy to ensure that adequate concentrations are attained with the new drug. If the levels are low, this may also be repeated at 8 weeks or 12 weeks following the introduction of the new PI (at the clinician's discretion).

Samples will be sent from sites using the normal procedures for dispatching samples for therapeutic drug monitoring which is performed in the UK by Delphic Diagnostics. Results will be made available to the sites within approximately 2 weeks of sample collection. When a trough sample was not available, population pharmacokinetic data will be used to predict the trough level. Advice on whether dose modification of the PI may be indicated will be provided with the result.

PI concentration will also be measured in patients who have a confirmed VL rebound, to assist with the interpretation of the causes of rebound. These samples may be stored for later testing in batches. The results will not be essential for clinical care because the patient will have switched back to triple-therapy. The results will be reported to the treating clinician but this may not be in real time.

7.4 Procedures for assessing health economics

The trial will measure all the costs (from an NHS perspective) of participants in the trial regardless of why costs were incurred throughout the trial. Data on service receipt (resource utilisation) will be collected at each trial visit by asking patients about visits to HIV clinics, outpatient (non-HIV) clinics, A&E departments or GPs, days off work due to illness, and any periods of hospitalisation since the previous documented trial visit. The duration of hospitalisation and main diagnosis will be recorded (verified if possible by reference to a hospital discharge summary). Patients will complete the EQ-5D questionnaire (see Appendix 5) at each trial visit to permit cost-utility analysis (27).

7.5 Trial closure

All patients will be followed according to the trial schedule until a common trial closing date. The trial will be closed after all data queries have been resolved.

8. LOSS TO FOLLOW-UP & WITHDRAWAL

8.1 Patient transfers

For patients moving from the clinical site, every effort should be made for the patient to be followed-up at another participating trial centre and for this trial centre to take over responsibility for the patient. MRC CTU should be informed and a copy of the patient CRFs will need to be provided to the new site. The patient will have to sign a consent form at the new site, and until this occurs, the patient remains the responsibility of the original centre.

8.2 Loss to follow-up

A patient will be regarded as 'lost to follow-up' if they have not been seen in clinic for more than 8 months. After this time, a check will be performed through disease databases or death registers (e.g. Office for National Statistics, CDSC Colindale). Consent will be obtained for this when the patient enters the trial (see Appendix 2). Subsequently, if the patient attends clinic and a CRF is received by the MRC CTU, the 'lost to follow-up' status will be reversed.

8.3 Withdrawal of consent

In consenting to the trial, patients are consenting to treatment according to the allocated treatment strategy as well as to trial follow-up visits and data collection. If a patient wishes to withdraw from the trial treatment strategy, the doctor/nurse will explain the importance of remaining on trial follow-up or, failing this, of allowing routine clinic follow-up data to be used for trial purposes.

If the patient explicitly states their wish not to contribute further data to the trial, the trial doctor should inform the MRC CTU in writing (i.e. a withdrawal CRF should be completed). Data up to the point of withdrawal can be included in the analysis. Further data i.e. vital status can only be obtained through NHS databases and registers (as above).

9. STATISTICAL CONSIDERATIONS

9.1 Method of randomisation

Patients will be randomised using a computer-generated algorithm based on random permuted blocks.

9.2 Outcome measures

Analyses will compare the PI monotherapy and standard treatment arms on the following outcome measures:

9.2.1 Primary outcome measure

- **Loss of future drug options**
  Defined as the first occurrence of intermediate to high level resistance to any one or more of the standard antiretroviral drugs (limited to licensed drugs in contemporary use) to which the patient’s virus was considered to be sensitive at trial entry (i.e. excluding drug resistance that was known to be present on previous resistance testing).

9.2.2 Secondary outcome measures

- **Serious drug or disease-related complication**
  Defined as the first occurrence of one of the following in any individual patient:

  - **Death from any cause**

  - **Serious AIDS-defining illness** (see diagnostic criteria, Appendix 8)

  - **Serious non-AIDS defining illness** (see diagnostic criteria, Appendix 9)
    - Acute myocardial infarction
    - Coronary artery disease requiring invasive procedures
    - Cirrhosis
    - Acute liver failure
    - End-stage renal disease
    - Stroke
    - Clinical acute pancreatitis
    - Severe lactic acidaemia
    - Severe facial lipoatrophy
    - Severe peripheral neuropathy
    - Non-AIDS malignancy

- **Adverse events**
  Defined as the total number of Grade III and IV adverse events.

- **Virological rebound**
  Defined as two consecutive tests, taken at least 4 weeks apart, with VL more than 50 copies/ml (the first test must also be confirmed by re-testing the same blood sample). There are a number of alternative definitions for virological rebound, each of which has advantages and limitations (28). The alternative definitions to be used in this trial will be described in the full statistical analysis plan.

- **CD4+ count change**
  Defined as change from baseline in absolute CD4+ count.

- **Health-related Quality of Life change**
  Defined as change from baseline in the mental and physical health summary scores.

- **Neurocognitive function change**
  Defined as change from baseline in the neurocognitive function summary score.

- **Cardiovascular risk change**
  Defined as change from baseline in the risk of cardiovascular disease calculated from the Framingham equation (19).

- **Health care costs**
  Defined as the total cost of health care resources utilised per patient year.

9.2.3 Health economic analysis

A full health economic analysis will be conducted to determine the relative cost-effectiveness of the alternative management approaches.

9.3 Sample size

As the strategy of PI monotherapy is likely to offer a major advantage in terms of cost (approximately 50% cost reduction from standard-of-care) and possible advantages in terms of long-term tolerability compared to standard-of-care, the trial aims to demonstrate that PI monotherapy is non-inferior to the standard-of-care by a pre-defined amount in terms of the primary endpoint (non-inferiority trial).

The estimation of the sample size is based on the following assumptions using a time-to-event analysis (29):

1. 90% of patients on the standard-of-care arm will maintain all future drug options (i.e. remain free of new resistance mutations) over three years. This figure is based on an analysis of resistance development during subsequent follow-up for patients who have taken an established NNRTI-based regimen for at least 12 months in the UK CHIC study, following the methodology used by Phillips et al (30).

2. The PI monotherapy arm will be considered "non-inferior" if the upper limit of the 95% confidence interval (2-sided) for the difference in the proportion of patients who maintain all future drug options over three years (standard-of-care – PI monotherapy) is less than 10%. This is consistent with the majority of HIV non-inferiority trials that use a cut-off of 10-15% and with FDA guidelines which recommend a cut-off of 10-12% (28, 31).

3. 85% power to detect non-inferiority according to criterion 2.

4. Recruitment occurs at a uniform rate over 18-24 months.

5. Total trial duration of 60 months.

6. Cumulative loss to follow-up is approximately 10% by 5 years.

Under these assumptions a total of 388 patients (194 per arm) are required. We therefore plan to recruit a total of 400 patients to yield approximately 40 events.

9.4 Interim monitoring and analyses

An Independent Data Monitoring Committee (IDMC) will be established to monitor the trial. A charter for the IDMC will be developed prior to starting the trial. The IDMC will meet once before the start of the trial, once within one year of start of recruitment, and at yearly intervals thereafter. In addition to the scheduled meetings, an IDMC meeting may be requested by IDMC members, the Chief Investigator or the Trial Steering Committee at any time. The IDMC will also review safety data at each meeting, and may make recommendations about the conduct of the trial should any safety concerns be identified.

9.5 Analysis plan

A full statistical analysis plan will be developed before the trial is analysed. It will be based on the following summary:

9.5.1 Primary analysis

The primary analysis will compare the two groups as allocated (intention to treat, ITT) in terms of loss of future drug options (see precise definition of primary outcome measure, above). Time-to-event methods (Kaplan-Meier plots and Cox proportional hazard regression) will be used for this comparison. Although non-inferiority trials often place especial emphasis on a per-protocol analysis, this is less relevant for a strategy trial (such as this) than for an explanatory trial comparing the therapeutic efficacy of two drugs. However, we will conduct a sensitivity analysis excluding patients who switch from their randomised allocation within 3 months where this is due to personal preference rather than any clinical indication. In both ITT and per-protocol analyses patients who have died or who are lost to follow-up will be censored at their last viral load measurement. If non-inferiority is demonstrated, an analysis for superiority of the PI monotherapy arm will be performed.

9.5.2 Secondary analyses

The primary analysis will be extended to tabulate individual ART drug options and ART class options which are lost due to the development of resistance. Time-to-event methods will be used to analyse the rate of serious drug- or disease-related complications and for viral load rebound. Also, the frequency of grade III and IV adverse events will be tabulated by body systems and randomised group and the groups will be compared using the X^2 test. Repeated measures analysis will be used to compare change in CD4+ cell count from baseline in the allocated strategies.

Patient responses on the MOS-HIV questionnaire will be converted to scores on 11 subscales, ranging from 0 to 100, with higher scores indicating better health (32). The scores are then synthesised into a physical health summary score (PHS) and a mental health summary score (MHS). A general linear mixed model will be used to compare the two groups in terms of changes from baseline in the summary PHS and MHS quality of life indexes over the follow-up period.

The scores on each of the neurocognitive tests will be standardised using demographic-adjusted normative means. This procedure will adjust the data for gender, age, level of education and ethnic background. Briefly, standard scores will be calculated by subtracting the appropriate normative mean from the raw score and then dividing by the appropriate normative standard deviation to give a z score (33). The patient's individual z scores on each test will be averaged to give the patient's neurocognitive function summary score at each

visit. A general linear mixed model will be used to compare the two groups in terms of changes from baseline in the neurocognitive function summary score.

Changes from baseline in the 10-year cardiovascular risk score will also be compared between the two groups using a general linear mixed model.

Economic analysis

An economic evaluation will be conducted from the health services perspective. Costs will cover the use of medication and laboratory tests as well as hospital, primary care and community health services. Unit costs will be attached to resource use, using the best available estimates of long run marginal opportunity cost, to obtain a cost per patient over the period of follow-up. Routinely available national unit costs will be used where possible (e.g. NHS Reference Costs, UK DoH 2005), with local estimations where necessary.

For the within-trial analysis, the differential cost of the treatment interventions will be related to their differential outcomes in terms of the primary outcome. The relative cost-effectiveness of the alternative forms of management will then be assessed using standard decision rules (34) and a full stochastic analysis will be undertaken. A cost-utility analysis will also be conducted based on EQ-5D health states. For each state, a utility is assigned as an adjustment factor for quality of life. Utility weights range from 0 to 1 where 0 represents death and 1 signifies perfect health. The total utility of a particular state is made up of the length of time spent in a state multiplied by the utility of that state. This will offer a simpler decision rule and allow explorations of cost per quality-adjusted life-year gained (QALY) (35). A cost consequence analysis will estimate, by randomised group, mean cost per patient and changes in EQ-5D 'utility'. Regression modelling will be used to explore variation in costs and utilities according to patient characteristics and by location of treatment (36) (37).

The within-trial analysis will be augmented by extrapolation beyond the trial follow-up using decision-analytic modelling (38). The aim of this analysis will be to predict the implications of any difference in clinical endpoints (in terms of drug therapy options, VL and viral sequencing for resistance mutations) in the trial for subsequent quality-adjusted survival duration and long-term resource costs. This will inform the question of whether any short-term savings in drug costs within the trial period are offset by additional costs or health decrements in the long-term. The model will probably take the form of a state transition model and is likely to be based on a model currently in development as part of the OPTIMA (www.optimatrial.org/uk) trial. The ultimate outputs of the economic evaluation will be estimates, by treatment group, of long-term quality-adjusted survival duration and costs including the presentation of incremental cost per quality adjusted life year as necessary. In addition, probabilistic methods will be used to present the probability that the two forms of management are cost-effective in the long-term. Scenario analysis will be used to explore the range of structural assumptions used in the analysis.

10. TRIAL MONITORING

10.1 Risk assessment

The trial will use only licensed drugs, and the risks and management of drug-related toxicities are known. The trial is classified as low risk and the monitoring plan has been designed accordingly.

10.2 Monitoring at MRC CTU

Data stored at MRC CTU will be checked for missing or unusual values (range checks) and checked for consistency within participants over time. If any such problems are identified, the site will be contacted and asked to verify or correct the entry. Any data which are changed should be crossed through with a single line and initialled. Changes should be made on the original CRF and a copy of the amended page(s) will be sent to the MRC CTU by fax or post. MRC CTU will send reminders for any overdue and/or missing data.

10.3 Clinical site monitoring

On-site monitoring will be conducted at all sites at a frequency of at least one visit per year. The first monitoring visit for each site will be performed after 4-6 patients have been enrolled or at 6 months following the date the site received approval to enrol patients, whichever occurs first. The CRFs of all patients enrolled will be reviewed at the first monitoring visit. For high-recruiting sites, a sample of no less than 5 patients will be selected by the MRC CTU for review at subsequent visits. Priority will be given to selecting patients for whom the CRFs have not been previously monitored.

MRC monitors will:

- verify completeness of Trial Master File
- confirm adherence to protocol
- review eligibility verification and consent procedures
- look for missed event reporting
- verify completeness, consistency and accuracy of data being entered on CRFs
- provide additional training as needed

The monitors will require access to all patient medical records including, but not limited to, laboratory test results and prescriptions. The investigator (or delegated deputy) should work with the monitor to ensure that any problems detected are resolved.

10.4 Data quality assurance

Data will be entered into the trial database at the MRC CTU from a copy of the CRF faxed or sent from the site. The site will retain the original CRF. Where possible de-identified laboratory reports (labelled with patient trial identification number) will be faxed to the MRC

CTU. All data recorded in each CRF will be entered on to the trial database, and then printed reports directly obtained from the database will be checked by different data-entry personnel.

10.5 Confidentiality of trial documents and patient records

The investigator must assure that patients' anonymity will be maintained and that their identities are protected from unauthorised parties. Patients will be assigned a trial identification number and this will be used on CRFs, patients will not be identified by their name. The investigator will keep securely a patient trial register showing identification numbers, names and date of birth.

11. SAFETY REPORTING

ICH GCP requires that both investigators and sponsors follow specific procedures when notifying and reporting adverse events/reactions in clinical trials. These procedures are described in this section of the protocol. Section 11.1 lists definitions, section 11.2 gives details of the institution/investigator responsibilities and section 11.3 provides information on MRC CTU responsibilities.

11.1 Definitions

The definitions of the EU Directive 2001/20/EC Article 2 based on ICH GCP apply in this trial protocol. These definitions are given in Table 1.

Table 1: Safety Reporting Definitions

Term	Definition
**Adverse Event (AE)**	Any untoward medical occurrence in a patient or clinical trial subject to whom a medicinal product has been administered including occurrences which are not necessarily caused by or related to that product.
**Adverse Reaction (AR)**	Any untoward and unintended response to an investigational medicinal product related to any dose administered.
**Unexpected Adverse Reaction (UAR)**	An adverse reaction, the nature or severity of which is not consistent with the information about the medicinal product in question set out in the Summary of Product Characteristics (or Investigator Brochure) for that product.
**Serious Adverse Event (SAE) or Serious Adverse Reaction (SAR) or Suspected Unexpected Serious Adverse Reaction (SUSAR)**	Respectively any adverse event, adverse reaction or unexpected adverse reaction that:<br>• results in death<br>• is life-threatening*<br>• requires hospitalisation or prolongation of existing hospitalisation**<br>• results in persistent or significant disability or incapacity<br>• consists of a congenital anomaly or birth defect

11.1.1 Clarifications and Exceptions

*The term 'life-threatening' in the definition of 'serious' refers to an event in which the patient was at risk of death at the time of the event; it does not refer to an event which hypothetically might have caused death if it were more severe.

**Hospitalisation is defined as an inpatient admission, regardless of length of stay, even if the hospitalisation is a precautionary measure for continued observation. Hospitalisations for a pre-existing condition (including elective procedures that have not worsened) do not constitute an SAE.

Medical judgement should be exercised in deciding whether an AE/AR is serious in other situations. Important AE/ARs that are not immediately life-threatening or do not result in death or hospitalisation but may jeopardise the subject or may require intervention to prevent one of the other outcomes listed in the definition above, should also be considered serious.

11.2 Institution/Investigator responsibilities

11.2.1 Investigator Assessment

(a) Seriousness
When an AE/AR occurs, the investigator responsible for the care of the patient must first assess whether the event is serious using the definition given in Table 1.

(b) Causality
The Investigator must assess the causality of all serious events/reactions in relation to the trial therapy using the definitions in Table 2. There are 5 categories: unrelated, unlikely, possible, probable and definitely related. If the causality assessment is unrelated or unlikely to be related the event is classified as a SAE. If the causality is assessed as either possible, probable or definitely related then the event is classified as a SAR.

Table 2: Definitions of causality

Relationship	Description	Event Type
**Unrelated**	There is no evidence of any causal relationship	SAE
**Unlikely**	There is little evidence to suggest there is a causal relationship (e.g. the event did not occur within a reasonable time after administration of the trial medication). There is another reasonable explanation for the event (e.g. the patient's clinical condition, other concomitant treatment).	SAE
**Possible**	There is some evidence to suggest a causal relationship (e.g. because the event occurs within a reasonable time after administration of the trial medication). However, the influence of other factors may have contributed to the event (e.g. the patient's clinical condition, other concomitant treatments).	SAR
**Probable**	There is evidence to suggest a causal relationship and the influence of other factors is unlikely.	SAR
**Definitely**	There is clear evidence to suggest a causal relationship and other possible contributing factors can be ruled out.	SAR

(c) Expectedness
If the event is a SAR the Investigator must assess the expectedness of the event. The definition of an unexpected adverse reaction (UAR) is given in Table 1 and is based on the information listed in the package insert or summary of product characteristics (available online at http://www.medicines.org.uk). If a SAR is assessed as being unexpected it becomes a SUSAR.

(d) Notification
Please also refer to the safety reporting flowchart on the following page
All AEs/ARs, whether expected or not, should be graded using the toxicity table in Appendix 7 and should be then recorded in the toxicity (symptoms) section of the appropriate visit CRF. This should be sent to the MRC CTU within one month of the form being due.

All non-treatment-related SAEs and all expected treatment-related SAEs (SARs) are exempted from expedited reporting i.e. only SUSARs require expedited reporting. (Adverse drug reactions to licensed PIs will continue to be reported by the investigator to the Yellow Card Scheme run by the MHRA and Commission on Human Medicines).

All SAEs/SARs, whether expected or not, should be graded using the toxicity table in Appendix 7 and should be then recorded on an Event CRF. Investigators should record all SAEs occurring from the time of randomisation until 30 days after the last protocol treatment administration.

A Safety Reporting Flowchart (Figure 2) is given at the end of this section to help explain the classification and reporting of events. Any questions concerning this process should be directed to the MRC CTU in the first instance. The notification procedure is as follows:

1. The Event CRF must be completed by the investigator (clinician named on the Delegation of Authority log who is responsible for the patient's care), with due care being paid to the grading, causality and expectedness of the event as outlined above. In the absence of the responsible investigator, the form should be completed and signed by a member of the site trial team. The responsible investigator should subsequently check the Event CRF, make changes as appropriate, sign and then re-fax to the MRC CTU as soon as possible. The initial report shall be followed by detailed, written reports as appropriate.

2. Send the Event CRF by fax to the MRC CTU, fax number: + 44 (0)20 7670 4817.

3. Follow-up: Patients must be followed-up until clinical recovery is complete and laboratory results have returned to normal or baseline, or until the event has stabilised. Follow-up should continue after completion of protocol treatment if necessary. Follow-up information should be noted on a further Event CRF by ticking the box marked 'follow-up' and faxing to the MRC CTU as information becomes available. Extra, annotated information and/or copies of test results may be provided separately. The patient must be identified by trial number, date of birth and initials only. The patient's name should not be used on any correspondence.

4. Staff at the institution must notify their local research ethics committee (LREC) of a SUSAR event (as per the institution's standard local procedure).

11.3 MRC CTU responsibilities

Medically qualified staff at the MRC CTU will review all event reports received. The causality assessment given by the local investigator at the hospital cannot be overruled and in the case of disagreement, both opinions will be provided in any subsequent reports.

The MRC CTU is undertaking the duties of trial sponsor and is responsible for the reporting of SUSARs and other SARs to the regulatory authority (MHRA) and the research ethics committees according to standard procedures and within standard timelines.

The MRC CTU will keep all investigators informed of any safety issues that arise during the course of the trial. Every 6 months the CTU will write to the investigators listing all SUSARs, providing information on the event, date of occurrence and body system. In addition, upon request of the TSC, the MRC CTU will inform investigators of any safety issues identified by the IDMC in their report(s) to the TSC.

SUSAR NOTIFICATION

Within one working day of becoming aware of an SUSAR, please fax a completed Event CRF to the MRC Clinical Trials Unit on:

Fax: +44 (0)20 7670 4817

Figure 2: Safety Reporting Flowchart

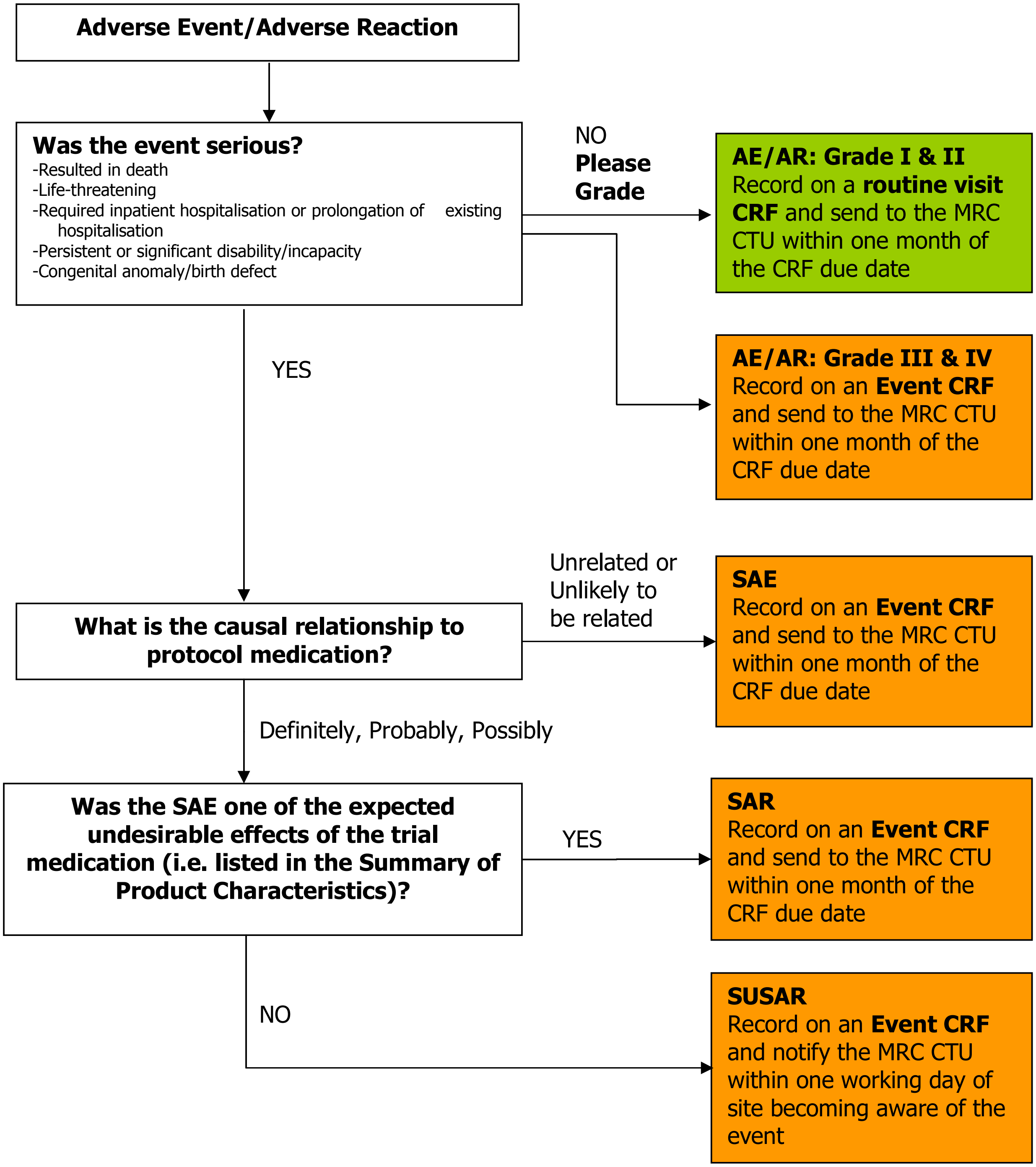

CRF: Case Report Form
SAE: Serious Adverse Event
SmPC: Summary of Product Characteristics
IB: Investigator's Brochure
SAR: Serious Adverse Reaction
SUSAR: Suspected Unexpected Serious Adverse Reaction

12. ETHICAL CONSIDERATIONS & APPROVAL

12.1 Ethical considerations

12.1.1 Risks and benefits to trial participants

See section 2.3 for detailed discussion of risks and benefits of the PI monotherapy strategy used in this trial. The risks include the risk of side effects arising from the change in medication, which are known and quantifiable since the trial will only use licensed drugs.

There is a very small increased risk of cardiovascular disease that may be partially abrogated by medication to treat hypercholesterolaemia, a potential increased risk of virological failure with the development of drug resistance, and the potential risk of virological rebound in the genital compartment with the theoretical increased risk of HIV transmission to others. These risks are all very small.

The benefits may be a potential reduced risk of long-term toxicity resulting from withdrawal of other medications, and the potential reduced risk of virological failure and drug resistance thereby leading to better preservation of future drug options. The trial may benefit society by finding an alternative long-term HIV disease management option.

12.1.2 Burden of investigations

The trial will use only licensed drugs, and the risk and management of drug-related toxicities are known. There is no placebo used. The protocol allows for switches within strategy. Apart from 2 extra monitoring blood tests in the first 6 months after enrolment in the PI monotherapy arm, the remainder of the trial visits coincide with the usual frequency of visits for routine clinical care, and the amount of extra blood taken is modest. Hence there are no ethical issues with the burden of investigations.

12.1.3 Informing potential trial participants of possible benefits and known risks

Participants will be informed fully of known risks and possible benefits by means of a patient information sheet and this will be reinforced by discussions with the trial research teams at the individual sites prior to enrollment.

12.1.4 Confidentiality

Patients' confidentiality will be maintained throughout the trial. Data submitted to MRC CTU and samples sent to central testing facilities will be identified only by the trial number and patient initials.

12.2 Ethical approval

The protocol will be submitted for ethical approval to an independent research ethics committee (REC) in the UK and for Site Specific Assessment at each of the participating sites. A copy of local R&D approval and the Patient Information Sheet and Consent Form on local-headed paper should be forwarded to the MRC CTU before the first patient is enrolled at site.

Each patient's consent to participate in the trial will be obtained after a full explanation has been given of the treatment options. The right of the patient to refuse to participate in the trial without giving reasons must be respected.

After the patient has entered the trial, the clinician must remain free to give alternative treatment to that specified in the protocol, at any stage, if he/she feels it to be in the best interest of the patient. However, the reason for doing so should be recorded and the patient

will remain within the trial for the purpose of follow-up and data analysis according to the treatment option to which they have been allocated. Similarly, the patient must remain free to withdraw from the trial medicine or trial follow-up, at any time, without giving reasons and without prejudicing his/her further treatment.

13. REGULATORY ISSUES

This trial will be reviewed by an independent research ethics committee (REC) in the UK. The trial will be registered with the Competent Authority, the Medicines and Healthcare products Regulatory Agency (MHRA) UK.

This trial has been granted a UK Clinical Trial Authorisation (CTA), reference 2007-006448-23.

Investigators may not enrol patients into this trial until:

- The necessary notification or approval of the protocol and any amendments has been given by the MHRA.
- The approval of the protocol and any amendments has been given by the REC.
- The approval of the institution's local ethics committee and R&D has been obtained.

During the course of the trial the MHRA or REC may request review of trial and data on patients involved in the trial.

14. INDEMNITY

The MRC is the sponsor of the trial. The MRC and NHS are both publicly funded bodies and are not allowed to purchase advance insurance to cover indemnity because they are backed by the resources of the Treasury.

14.1 Liability

14.1.1 Circumstances where MRC will accept liability

The MRC is willing to accept liability in cases where:

(i) it sponsors the research: namely where it takes responsibility for initiating, managing and having day-to-day oversight of the research in question (including any research carried out by its Units); and

(ii) the MRC, or any of its employees, or any person formally acting with Council's authority, have been negligent or have failed to adhere to the relevant guidelines/guidance, legislation or procedure on good practice in relation to medical research; and

(iii) that negligence or failure to adhere to legislation, etc has caused or has materially contributed to the harm suffered by the individual making the claim.

Except in cases where a no-fault compensation scheme has been established with respect to a specified clinical trial, where there has been no negligence and no lapse in procedures or adherence to relevant legislation or accepted best practice on the part of the MRC, the MRC will not accept legal liability for any injury or harm suffered by a participant of a research project (in other words the MRC will not accept that the MRC is legally required to make any payment in respect of any injury or death arising from a research project). In such circumstances, the MRC will, on a voluntary basis, be prepared to consider making an *ex gratia* payment to any individual who suffers harm as a result of being involved in the research, but only if:

(iv) the MRC is the sponsor of the research (as in 14.1.1 (i) above); and

(v) where the MRC is a joint sponsor of the research, the harm has not occurred due to the negligence or other fault of the other sponsor(s) - in such cases the MRC would expect that the other sponsor would provide indemnity.

Requests for compensation or *ex gratia* payments in such circumstances will be considered on a case-by-case basis in relation to the MRC policy on such payments.

14.1.2 Circumstances where MRC will not accept liability

Where medical research is carried out in a care organisation, whether the NHS or another organisation, that care organisation will continue to owe the same duty of care to patients who are also participants in research that it owes to any other patients. This duty is not affected by patients agreeing to participate in such research or the fact that the MRC may be the sponsor of such research. Care organisations (and not the MRC) continue to be responsible for any breaches of that duty of care with respect to participants of research. Similarly, responsibility for the quality of investigational products (e.g. investigational medicinal products, investigational devices) lies with the manufacturer of the product.

If the patient is harmed as a result of negligence of NHS staff while participating in this trial, then they may be able to seek compensation. In this situation indemnity is provided by NHS indemnity schemes and professional indemnity schemes.

15. FINANCE

The trial is funded by the NIHR Health Technology Assessment Programme. The HTA programme is part of the National Institute for Health Research. It produces independent research information about the effectiveness, costs and broader impact of healthcare treatments and tests for those who plan, provide or receive care in the NHS.

The trial will be coordinated by the MRC CTU. A written agreement with the site Principal Investigator and/or the Investigator's institution and the MRC CTU will outline the funding arrangements to sites.

16. TRIAL COMMITTEES

16.1 Trial Management Group

A trial management group (TMG) that will include the MRC CTU trial physician and/or chief investigator, the trial statistician, the MRC trial manager and data manager will be formed to conduct the day-to-day management of the trial. A charter will be developed to describe the functioning of the TMG.

16.2 Trial Steering Committee

A Trial Steering Committee (TSC) will be formed to provide overall supervision for the trial. This will include an independent Chairman, two other independent clinicians, a community representative, the Chief Investigator, external clinical principal investigator and the trial statistician. A charter will be developed to describe the functioning of the TSC.

16.3 Independent Data Monitoring Committee

An Independent Data Monitoring Committee (IDMC) will be formed comprising two clinicians, a community representative, and a statistician, none of whom have direct involvement with the trial. The IDMC will report to the TSC. A charter will be developed to describe the functioning of the IDMC.

16.4 Endpoint Review Committee

An Endpoint Review Committee (ERC) will be formed to review the documentation relating to reported serious drug or disease-related clinical events, in order to ascertain whether these meet the criteria for this secondary endpoint. The ERC will consist of two clinicians, one of whom will not be directly involved with the trial.

Figure 3: Diagram of relationships between trial committees

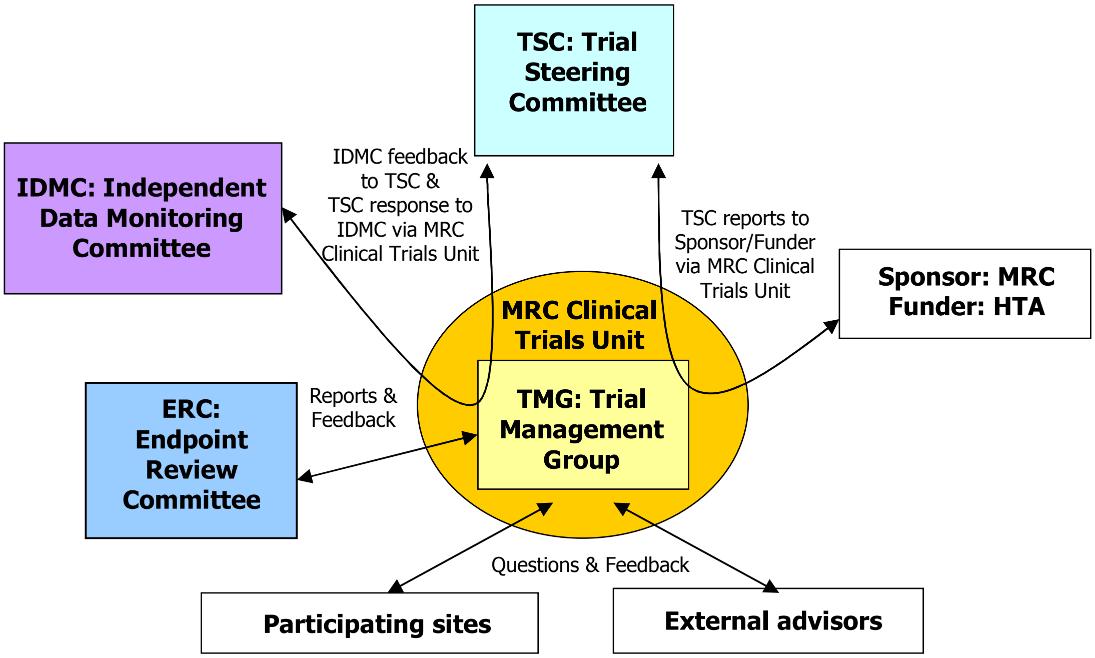

17. PUBLICATION

It is anticipated that a number of opportunities will arise for publication during the course of, and following completion of this trial. In order to avoid disputes regarding authorship, it is important to establish a consensus approach that will provide a framework for all publications derived in full or in part from this clinical trial. The following approach is derived from the Lancet and from the publication policies used in OPTIMA and ESPRIT studies:

- All publications are to be approved by the TMG and TSC before submission for publication. The TMG and TSC will resolve problems of authorship and maintain the quality of publications.

- Trial findings will be submitted to journal(s) that support open access publication within the time frame specified by the MRC policy. All publications will acknowledge the HTA and any other appropriate funding sources.

- For all publications, the TSC will either nominate a chairperson or approve an individual's request to chair a manuscript writing committee. The chair will usually be the primary or senior author. The chairperson is responsible for identifying fellow authors and for determining with that group the order of authorship that will appear on the manuscript. The proposed writing committee will be submitted to the TSC for ratification prior to the first draft of the manuscript.

- The chairperson of any writing committee will also provide the TSC with a list of investigators to be presented in an appendix at the end of the paper. This list will include investigators who contributed to the investigation being reported but who are not members of the writing committee. In principle, substudy reports should include all investigators for the main study, although in some instances where a smaller number of investigators have made any form of contribution, it may be appropriate to abbreviate the listing. All headline authors in any publication arising from the main study or sub-studies must have a made a significant academic/project management contribution to the work that is being presented. "Significant" must be defined by a written declaration of exactly what the contribution of any individual is believed to have been. In addition to fulfilling the criteria based on contribution, additional features that will be considered in selecting an authorship group will include the recruitment of patients who contributed data to any set of analyses contained in the manuscript, and /or the conduct of analyses (laboratory and statistical), leadership and coordination of the project in the absence of a clear academic contribution.

- The data derived from this clinical trial are considered the property of the MRC. The presentation or publication of any data collected by the participating investigators on patients entered into this trial is under the direct control of the TMG and TSC. This is true whether the publication or presentation is concerned directly with the results of the trial or is associated with the trial in some other way. However, although individual participating investigators will not have any inherent right to perform analyses or interpretations or to make public presentations or seek publication of any of the data other than under the auspices of and with the approval of the TMG and TSC, they will be encouraged to develop sub-studies or propose analyses subject to the approval by the TMG and TSC.

- Outcome data by treatment group will not be revealed to the participating investigators until the data collection phase of the trial has been completed. This policy safeguards against possible bias affecting the data collection. The IDMC will be monitoring the outcome results and may recommend that the trial be stopped for safety reasons or if a definitive answer is reached earlier than the scheduled end of the trial.

18. REFERENCES

1. Agency HP. A Complex Picture: HIV and other Sexually Transmitted Infections in the United Kingdom: 2006. Annual Report; 2006.
2. Strategies for Management of Antiretroviral Therapy Study Group. CD4+ guided interruption of antiretroviral treatment. New England Journal of Medicine. 2007;355:2283-96.
3. Phillips AN, Gazzard BG, Clumeck N, Losso MH, Lundgren JD. When should antiretroviral therapy for HIV be started? BMJ. 2007;334(7584):76-8.
4. Pulido F, Arribas JR, Delgado R, Cabrero E, Gonzalez-Garcia J, Perez-Elias MJ, et al. Lopinavir-ritonavir monotherapy versus lopinavir-ritonavir and two nucleosides for maintenance therapy of HIV. AIDS (London, England). 2008 Jan 11;22(2):F1-9.
5. Arribas JR, Pulido F, Delgado R, González-García J, Pérez-Elías MJ AA, Portilla J, Pasquau J, Iribarren JA, Rubio R, Ocampo A, Miralles P, Knobel H, Gaya F, Muñoz RM, Clotet B, Podzamcer D, OK04 Study Group. Lopinavir-ritonavir Monotherapy vs Lopinavir-ritonavir and Two Nucleosides for Maintenance Therapy of HIV. Ninety-six Week Results of a Randomized, Controlled, Open Label, Clinical Trial (OK04 Study). 11th European AIDS Conference; Madrid, Spain; 2007.
6. Cameron W, da Silva B, Arribas J, et al. A two-year randomised controlled clinical trial in antiretroviral-naive subjects using lopinavir/ritonavir (LPV/r) monotherapy after initial induction treatment compared to an efavirenz (EFV) 3-drug regimen (Study M03-613). 2006.
7. Nunes EP, Oliveira MS, Almeida MMTB, et al. 48-week efficacy and safety results of simplification to single agent lopinavir/ritonavir (LPV/r) regimen in patients suppressed below 80 copies/mL on HAART - the KalMo study. XVI International AIDS Conference; Toronto, Canada; 2006.
8. Delfraissy JF, Flandre P, Delaugerre C, Ghosn J, Horban A, Girard PM, et al. Lopinavir/ritonavir monotherapy or plus zidovudine and lamivudine in antiretroviral-naive HIV-infected patients. AIDS (London, England). 2008 Jan 30;22(3):385-93.
9. Swindells S, Wilkin T, DiRenzo G, et al. A prospective, open-label, pilot trial of regimen simplification to atazanavir/ritonavir alone as maintenance antiretroviral therapy after sustained virologic suppression (ACTG 5201). 13th Conference on Retroviruses and Opportunistic Infections; Denver, USA; 2006.
10. Karlstrom O, Josephson F, Sonnerborg A. Early virologic rebound in a pilot trial of ritonavir-boosted atazanavir as maintenance monotherapy. Journal of acquired immune deficiency syndromes (1999). 2007 Apr 1;44(4):417-22.
11. Arribas JR, Pulido F. Early virologic rebound in a pilot trial of ritonavir-boosted atanazavir as maintenance monotherapy. Journal of acquired immune deficiency syndromes (1999). 2007 Sep 1;46(1):118; author reply -9.
12. Mohammed P. Tibotec. Personal Communication. 2007.
13. Friis-Moller N, Reiss P, Sabin CA, Weber R, Monforte A, El-Sadr W, et al. Class of antiretroviral drugs and the risk of myocardial infarction. The New England journal of medicine. 2007 Apr 26;356(17):1723-35.
14. Stein JH. Cardiovascular risks of antiretroviral therapy. The New England journal of medicine. 2007 Apr 26;356(17):1773-5.
15. Arribas JR, Pulido, F., Delgado, R., et al. Lopinavir/ritonavir as single-drug therapy for maintenance of HIV-1 viral suppression: 48-week results of a randomized, controlled,

open-label, clinical trial (OK Study). XVI International AIDS Conference; Toronto, Canada; 2006.

16. Solas C, Lafeuillade A, Halfon P, Chadapaud S, Hittinger G, Lacarelle B. Discrepancies between protease inhibitor concentrations and viral load in reservoirs and sanctuary sites in human immunodeficiency virus-infected patients. AntimicrobAgents Chemother. 2003;47(1):238-43.
17. Giancola ML, Lorenzini P, Balestra P, Larussa D, Baldini F, Corpolongo A, et al. Neuroactive antiretroviral drugs do not influence neurocognitive performance in less advanced HIV-infected patients responding to highly active antiretroviral therapy. Journal of acquired immune deficiency syndromes (1999). 2006 Mar;41(3):332-7.
18. Vernazza P, Daneel S, Schiffer V, Decosterd L, Fierz W, Klimkait T, et al. The role of compartment penetration in PI-monotherapy: the Atazanavir-Ritonavir Monomaintenance (ATARITMO) Trial. AIDS (London, England). 2007 Jun 19;21(10):1309-15.
19. Wilson PW, D'Agostino RB, Levy D, Belanger AM, Silbershatz H, Kannel WB. Prediction of coronary heart disease using risk factor categories. Circulation. 1998 May 12;97(18):1837-47.
20. La Porte C, Back D, Blaschke T. Updated guidelines to perform therapeutic drug monitoring for anitretroviral agents. Reviews in Antiviral Therapy. 2006;3:4-14.
21. Sekar VJ, Lefebvre E, Marien K, De Pauw M, Vangeneugden T, Hoetelmans RM. Pharmacokinetic interaction between darunavir and saquinavir in HIV-negative volunteers. Therapeutic drug monitoring. 2007 Dec;29(6):795-801.
22. Wu AW, Rubin HR, Mathews WC, Ware JE, Jr., Brysk LT, Hardy WD, et al. A health status questionnaire using 30 items from the Medical Outcomes Study. Preliminary validation in persons with early HIV infection. MedCare. 1991;29(8):786-98.
23. Manji H, Miller R. The neurology of HIV infection. Journal of neurology, neurosurgery, and psychiatry. 2004 Mar;75 Suppl 1:i29-35.
24. Ances BM, Ellis RJ. Dementia and neurocognitive disorders due to HIV-1 infection. Seminars in neurology. 2007 Feb;27(1):86-92.
25. Carey C, Woods S, Ripeth J, Gonzalez R, Moore D, Marcotte T, et al. Initial Validation of a Screening Battery for the detection of HIV-associated Cognitive Impairment. Clinical Neuropsychology. 2004;18(2):234-48.
26. Bowie CR, Harvey PD. Administration and interpretation of the Trail Making Test. Nature protocols. 2006;1(5):2277-81.
27. Brooks R. EuroQol: the current state of play. Health Policy. 1996;37(1):53-72.
28. Center for Drug Evaluation and Research, US Department of Health and Human Services, Food and Drug Administration. Guidance for Industry. Antiretroviral Drugs Using Plasma HIV RNA Measurements - Clinical Considerations for Accelerated and Traditional Approval; 2002.
29. Barthel FM, Babiker A, Royston P, Parmar MK. Evaluation of sample size and power for multi-arm survival trials allowing for non-uniform accrual, non-proportional hazards, loss to follow-up and cross-over. Statistics in medicine. 2006 Aug 15;25(15):2521-42.
30. Phillips AN, Dunn D, Sabin C, Pozniak A, Matthias R, Geretti AM, et al. Long term probability of detection of HIV-1 drug resistance after starting antiretroviral therapy in routine clinical practice. AIDS (London, England). 2005;19(5):487-94.
31. Parienti JJ, Verdon R, Massari V. Methodological standards in non-inferiority AIDS trials: moving from adherence to compliance. BMC medical research methodology. 2006;6:46.

32. Ware JE, Jr., Sherbourne CD. The MOS 36-item short-form health survey (SF-36). I. Conceptual framework and item selection. MedCare. 1992;30(6):473-83.

33. Robertson KR, Smurzynski M, Parsons TD, Wu K, Bosch RJ, Wu J, et al. The prevalence and incidence of neurocognitive impairment in the HAART era. AIDS (London, England). 2007 Sep 12;21(14):1915-21.

34. Drummond MF, Sculpher MJ, Torrance GW, O'Brien BJ, Stoddart GL. Methods for the Ecomonic Evaluation of Health Care Programmes: Oxford University Press 2005.

35. Klarman H, Francis J, Rosenthal G. Cost-effectiveness analysis applied to the treatment of chronic renal disease. MedCare. 1968;6:48-54.

36. Hoch JS, Briggs AH, Willan AR. Something old, something new, something borrowed, something blue: a framework for the marriage of health econometrics and cost-effectiveness analysis. Health Econ. 2002;11(5):415-30.

37. Manca A, Rice N, Sculpher MJ, Briggs AH. Assessing generalisability by location in trial-based cost-effectiveness analysis: the use of multilevel models. Health Econ. 2005;14(5):471-85.

38. Briggs A, Claxton K, Sculpher M. Decision Modelling for Health Economic Evaluation. First ed: Oxford University Press; 2006.

APPENDICES

APPENDIX 1: PATIENT INFORMATION SHEET
APPENDIX 2: CONSENT FORM
APPENDIX 3: GP LETTER
APPENDIX 4: PARTICIPATING SITES
APPENDIX 5: EQ-5D QUESTIONNAIRE
APPENDIX 6: MOS-HIV QUESTIONNAIRE
APPENDIX 7: TOXICITY TABLE
APPENDIX 8: DIAGNOSTIC CRITERIA FOR SERIOUS AIDS-DEFINING ILLNESS
APPENDIX 9: DIAGNOSTIC CRITERIA FOR SERIOUS NON-AIDS-DEFINING ILLNESS
APPENDIX 10: PROTEASE INHIBITOR PRODUCT INFORMATION
APPENDIX 11: JOINT BRITISH SOCIETIES CARDIOVASCULAR RISK PREDICTION CHARTS
APPENDIX 12: LABORATORY METHODS & SPECIMEN STORAGE
APPENDIX 13: NEUROCOGNITIVE ASSESSMENT TOOLS
APPENDIX 14: PROTOCOL VERSION 2.0 LISTING OF CHANGES

APPENDIX 1: PATIENT INFORMATION SHEET

[To be presented on local-headed paper]

Patient Information Sheet – Part 1

Version 2.0, 18 November 2008

1. Study title

PIVOT: Protease Inhibitor monotherapy Versus Ongoing Triple-therapy in the long-term management of HIV disease

EUDRACT: 2007-006448-23
ISRCTN04857074

2. Invitation

You are being invited to take part in a research study. Before you decide whether to take part it is important for you to understand why the research is being done and what it will involve. Please take time to read the following information carefully and discuss it with others if you wish.

- Part 1 tells you the purpose of this study and what will happen to you if you take part.
- Part 2 gives you more detailed information about how the study will be carried out.

Ask us if there is anything that is not clear or if you would like more information. Take time to decide whether or not you wish to take part. Thank you for reading this.

3. What is the purpose of the study?

Currently, we use a combination of three drugs to treat HIV. These drugs are called anti-retroviral treatment or ART. The drugs that usually make up ART are:

- two nucleoside reverse transcriptase inhibitors (NRTIs)
- one non-nucleoside reverse transcriptase inhibitor (NNRTI) or a protease inhibitor (PI)

These drugs seem to be very effective and do not cause many side effects in the short-term, but they do need to be taken life-long and may be associated with side effects after many years. It is possible that there are alternatives to triple therapy that are just as effective and have less long-term side effects. It is also possible that there are alternatives that, in the long run, will be less susceptible to develop drug resistance and so will preserve more treatment choices for the future.

One possible long-term treatment option is to simplify treatment to just one drug, a PI, instead of the standard triple therapy. There is some evidence from previous clinical trials that patients who are stable on triple-therapy can simplify their treatment to PI monotherapy (one drug) and successfully maintain undetectable viral load as well as experience less side effects when they stop their NRTIs.

The purpose of this study is to find out whether a strategy of trying PI monotherapy, with the plan of switching back to triple therapy if viral load control is not adequate, is just as good as continuing standard triple-therapy in long-term treatment of HIV disease.

4. Why have I been chosen?

You have been chosen for this study because you are taking standard combination ART, described above, and you have had an undetectable viral load for at least the last 6 months. Overall 400 patients like you will take part in the study.

5. Do I have to take part?

It is up to you to decide whether or not to take part. If you do decide to take part you will be given this information sheet to keep and be asked to sign a consent form. If you decide to take part you are still free to withdraw at any time and without giving a reason. A decision to withdraw at any time, or a decision not to take part, will not affect the standard-of-care you receive.

6. What will happen to me if I take part?

Study screening and entry

Before you join the study, we will need to review your medical history and do some tests to make sure you are suitable. You will need to come to the clinic in the morning with an empty stomach (nothing to eat or drink from midnight before the visit, other than water and your medication) so that we can measure your cholesterol and glucose as well as some other routine blood tests. We will also need to perform an ECG (an electrical recording of your heart). Some of these tests may not need to be repeated if you have had them done in the previous 6 months. If these tests do not show any major abnormalities and you meet all the criteria for the study, then will be able to participate in the study.

Study treatment

The study is a randomised controlled clinical trial. This means that once you have agreed to enter the trial, you will be allocated by chance to either the standard triple-therapy group or the PI monotherapy group. Everyone has an equal chance of being in either group. Allocating treatment this way means that the groups of people getting each treatment should be similar, so that if there are any differences between how the groups do we know it will be due to the treatment allocation.

If you are allocated to the standard treatment group, you will carry on with your current treatment and continue to have regular monitoring as described in the next paragraph. You will be able to change your therapy in the future if there is a good clinical reason to do so (for example, if you develop viral load rebound or side-effects from the drugs), but if you change therapy it should be according to current guidelines (i.e. should consist of at least 3 drugs which are effective against HIV).

If you are allocated to the PI monotherapy group, you will stop all your current ART drugs that are not PIs, and will start or continue on just a boosted PI (boosted means it is given with a small dose of ritonavir, another PI, to increase the level of the main PI and to allow less frequent dosing). There are several PIs that may be considered for use as monotherapy, and the choice of which PI to take will be left up to you and your clinic doctor to decide. Your clinic doctor will be able to advise you on the relative merits of the different PIs, as they may differ in terms of the dose frequency, potential side effects, and possibly in effectiveness. You will be able to change to an alternative PI as monotherapy during the study if you develop any side-effects or if there are other reasons why you, or your doctor, believe that an alternative PI might be better for you. You will be expected to continue treatment with PI monotherapy until the end of the study, but you will be able to return to standard triple therapy if there is a good clinical reason to do so (for example you develop viral load rebound, or you develop side effects from the PI that you are on that cannot be controlled satisfactorily with additional medication or by switching to an alternative PI). If you do need to return to taking standard triple-therapy that will usually be the same combination that you were taking when you entered the study. During the study you will continue to have regular monitoring as described in the next section.

Study assessments and follow-up

You will be involved in the study for about 3 to 5 years (depends on how long the study has already been running when you join), during which time you will need to visit the clinic on a regular basis to check how you are doing. However, the clinic visits are at approximately the same frequency that you would be coming to the clinic anyway for routine care, even if you were not taking part in the study. The visits will be at Day 0 (the day you enter the study and that you change to PI monotherapy, if allocated to that option) and then approximately every 12 weeks up to the end of the study.

At each of these routine visits the research nurse and/or your doctor will ask you a few questions about your medication and whether you have any symptoms, and may perform a brief examination. They will ask you to complete a checklist of your health status (takes less than 1 minute to do) and will draw some blood for routine blood tests (viral load, CD4 count, full blood count, kidney and liver function tests). These routine visits (which account for most of the visits in the study) are very similar to what you would have done anyway as part of your normal care.

At the week 12 visit, the annual study visits (every 48 weeks) and the last study visit, you will need to come in the morning with an empty stomach (nothing to eat or drink from midnight before the visit, other than water and your medication) so that we can also measure the level of cholesterol and glucose in your blood. We will also take an extra tube of blood at this visit to store in case future tests are needed. In addition to the assessments done at the routine visits, we will also do some neurocognitive (brain function) tests that involve testing your memory and reaction times, and ask you to fill in a quality of life questionnaire. These simple tests will take about 15 minutes in total.

If you are allocated to the PI monotherapy group, you will also need to come for two extra visits, at 4 weeks and 8 weeks after you start on your new treatment. For the week 4 visit, you will need to come in the morning with an empty stomach (nothing to eat or drink from midnight before the visit, other than water and your non-ART medication) and postpone your morning dose of PI medication until after the blood has been drawn. At this visit the research nurse or doctor will review how you are getting on with the new treatment, and will draw some blood for routine tests (including viral load, full blood count, kidney and liver function, cholesterol and glucose) and to measure the concentration of the PI in your blood. We will also take 1 extra tube of blood for storage in case of the need for future tests. At the week 8 visit, you will just have a blood sample taken for measurement of viral load (and possibly to check the PI concentration again, if the levels were low on the first test).

The total amount of blood needed for these routine tests will be about 40ml at screening, (8 teaspoons), 25ml (5 teaspoons) at day 0 and for most of the routine 12 weekly visits, and 35ml (7 teaspoons) at the week 12, annual and final study visits. For the week 4 and week 8 visits for those in the PI monotherapy group only, the amount of blood needed will be about 35ml (7 teaspoons) and 10ml (2 teaspoons) respectively. Almost all these blood samples would need to be drawn anyway for your routine care and the total amount of additional blood taken for specific research-related tests will average no more than 15ml (3 teaspoons) per year during the study. You will also need to provide a urine sample at screening and at annual study visits for routine tests, and a small amount of this (approximately 5ml, 1 teaspoon) will be stored in case of the need for future tests.

In addition to these routine visits, if you have a rebound of viral load while you are in the study you will be recalled for a repeat viral load test at between 4 to 6 weeks from the date of the previous test. Two additional tubes of blood will also be collected for storage (8ml or 2 teaspoons) in case of the need for future tests. If the viral load test confirms a rebound of viral load your clinic doctor will discuss with you the need to change your treatment. If you are in the PI monotherapy arm, this will mean restarting triple-therapy. If you have viral load

rebound, the sample will also be sent for a resistance test, the results of which may guide you and your doctor as to the best choice of medication for you. At the end of the study all samples with viral load rebound will be tested again using a very sensitive resistance test at a central laboratory to give the best information possible on resistance. All these results will be provided to your doctor.

You will not be paid to take part in the study, but your clinic may be able to reimburse reasonable travel expenses.

7. What do I have to do?

There are no particular lifestyle or dietary restrictions required for participating in this study, other than those associated with taking your medication each day. You will be expected to adhere to your study medication according to your allocated treatment group for the duration of the study, and to come for the clinic visits according to the study schedule. You will need to tell your doctor before you take any other medication or herbal treatments as some of these may interact with the PI or your other HIV medications.

8. What are the alternatives for treatment?

The alternative for treatment, if you don't participate in this trial, is to simply carry on with your usual combination HIV therapy, or to switch to other alternative treatments based on combinations of currently-licensed ART drugs. If you are experiencing unpleasant side effects from your current therapy then you should discuss with your doctor about switching to an alternative triple-therapy for at least 3 months before entering this trial. You should not enter the study if the side effects of your current therapy are so bad that they mean you would not be prepared to continue the treatment you are currently on for the duration of the study (which will be at least 3 years) if you were allocated to the combination therapy group.

9. What are the side-effects of any treatment received when taking part?

Both standard therapy and PI monotherapy may cause side effects during the course of this study. The side effects of PIs vary according to the particular drug taken, but the PIs that are most often prescribed now are generally well tolerated. The commonest side effects are gastrointestinal intolerance (such as diarrhoea or nausea), metabolic disturbances (such as high cholesterol and high blood sugar) and increased bilirubin level (which is harmless, but produces a yellow discoloration in the eyes). These side effects are mainly associated with particular drug(s), and can usually be managed by taking additional medication (e.g. to lower cholesterol) or by switching to an alternative PI. Your doctor will discuss with you the possible side effects of the PI that you choose to use, and will advise you on the appropriate management of any side-effects.

If you do develop any symptoms you should report them to your doctor. If you experience any serious side effects you should contact a member of the study team according to the contact details given on the emergency contact card.

10. What are the possible disadvantages and risks of taking part?

Possible risks and discomforts include the development of local bruising and discomfort where needles are inserted into a vein to collect blood, although you would be having blood taken as part of your routine care with approximately the same frequency even if you were not participating in this study.

If you are not taking a boosted PI-containing combination when you enter the study, there is a risk that you will develop new side effects if you are allocated to the PI monotherapy group and need to start a boosted PI. However, these side effects (outlined in question 9) can usually be controlled with changes in dose, switch to an alternative PI medication, or use of additional medication (e.g. to reduce cholesterol).

We expect that a small proportion of patients will experience low level viral load rebound when they switch to PI monotherapy (likely no more than 5-10%). Those who do rebound will need to switch promptly back to triple-therapy. It is almost certain, based on scientific knowledge and results from other studies, that reintroduction of combination therapy will be successful in suppressing viral load to undetectable levels again. The risk of developing clinical resistance to the PI during this short time with a low level of viral load is very small. Overall, the risk of developing drug resistance appears to be no greater with PI monotherapy treatment than it is with triple-therapy. If you do develop resistance during the study, there are likely to be several other options available to you for treatment that have a high chance of success, and your clinic doctor will discuss these with you.

If you are taking PI monotherapy there is a theoretical risk of developing viral rebound and drug resistance in parts of the body where PIs do not achieve high levels (such as the brain and genital tract), even though the treatment is working well in suppressing viral replication in your blood. There is no evidence to date that this is associated with any adverse clinical consequences for you. If you have detectable viral load in genital secretions, there is a theoretically increased risk that you might transmit HIV to someone else if you do not practise safe sex. You should consider this risk to others carefully and we suggest that you discuss this with any regular sexual partner(s) before deciding whether or not to participate in this study.

If you are a woman who is pregnant, you should not enter this study. This is because current guidelines recommend that pregnant women should take combination therapy (PI monotherapy has not been tested) to prevent transmission to their unborn child. Although pregnant women have taken PIs in the past without harming the baby, it is possible that taking a PI during pregnancy may harm the unborn child. If you find that you have become pregnant while taking part in the study, you should immediately tell your study doctor. Any necessary changes to your ART combination during the pregnancy are permitted in this study.

If you have private medical insurance you should check with the company before agreeing to take part in the trial to ensure that your participation will not affect your medical insurance. In this situation you also need to consider whether disclosure of your HIV status may also affect your insurance.

11. What are the possible benefits of taking part?

If you take part and are allocated to the PI monotherapy group, you may have a reduced risk of long-term side effects by stopping the drugs you were taking as part of your triple combination therapy. The long-term side effects that you may be spared depend on the particular drugs that you stopped. They may include things like kidney damage, liver damage, nerve damage, loss of fat in the face, arms and legs and other long-term unwanted side-effects, both known and unknown, associated with NRTIs; metabolic disturbances (e.g. high cholesterol) and chronic neuropsychiatric problems (e.g. sleep disturbance, memory loss, poor concentration) associated with NNRTIs (especially efavirenz).

There is also a possibility that you may have less drug resistance and maintain more future long-term treatment options at the end of the study if you take PI monotherapy (because PI drugs are generally less prone to develop resistance).

You may also appreciate or benefit from some of the extra testing that is done as part of this study, for example the neurocognitive tests, the PI drug level testing (in the PI monotherapy group), the more detailed drug resistance testing as well as the extra attention to details of your care that is associated with participation in a clinical trial.

If you take part in this study, you'll be helping us to learn more about the best ways to treat people with HIV. Having people like you join a randomised controlled trial is the only way that we can find out for sure whether PI monotherapy is as good as (or perhaps better than) conventional combination treatment for the long-term management of HIV disease. If the study does show that PI monotherapy is at least as good as conventional treatment then this will be an important finding. It will increase the number of treatment options available for long-term management of HIV disease and this may be of benefit to you and other people like you in the future. PI monotherapy is more economical than triple-therapy, so if it works as well as triple-therapy, we'll be able to free up healthcare resources that could be used to improve other aspects of HIV care.

12. What happens when the research study stops?
When the study ends, you will still be able to continue to access medication and continue to be monitored as part of routine clinical care. If you are doing well on PI monotherapy you could continue this if you and your doctor wish to do so, or switch back to combination triple-therapy while you wait to hear about the results from the trial. If you are on combination therapy, it would be sensible to continue this while you wait for the results of the trial. When the results of the trial are available your doctor will talk with you about which option would be the best for you in the long-term.

13. What if there is a problem?
Any complaint about the way you have been dealt with during the study or any possible harm you might suffer will be addressed individually. Detailed information on this is given in Part 2 of the information sheet.

14. Will my taking part in this study be kept confidential?
Yes, all the information about your participation in this study will be kept confidential. The details are included in Part 2.

15. Contact for Further Information
If you have any further questions about this study please discuss them with your doctor. You may also find it helpful to contact the i-Base Treatment Information phone line: 0808 800 6013 (open Mon-Wed 12-4pm), or website: www.i-base.org.uk

If you would like further information on this study please ask:

…………………………………………………… who can be contacted at ……………………………………….

or ………………………………………………...at …………………………………. who is the local principal investigator.

This completes Part 1 of the Information Sheet.

If the information in Part 1 has interested you and you are considering participation, please continue to read the additional information in Part 2 before making any decision.

[To be presented on local-headed paper]

Patient Information Sheet – Part 2

Version 2.0, 18 November 2008

1. Study title

PIVOT: Protease Inhibitor monotherapy Versus Ongoing Triple-therapy in the long-term management of HIV disease

EUDRACT: 2007-006448-23
ISRCTN04857074

2. What if relevant new information becomes available?

Sometimes, during the course of a research project, vital new information becomes available about the treatment that is being studied. If this happens, your study doctor will tell you about it and discuss with you whether you want to, or whether you should, continue with the study. If you decide not to carry on, your study doctor will make arrangements for your care to continue. If you decide to continue in the study you will be asked to sign an updated consent form.

Also, on receiving new information your study doctor might consider it to be in your best interests to withdraw you from the study treatment. Your study doctor will explain the reasons and arrange for your care to continue. If the study is stopped for any other reason, you will be told why and your continuing care will be arranged.

3. What will happen if I don't want to carry on with the study?

You can decide that you no longer wish to receive study treatment at any time. We would however like to keep in contact with your doctor so that we can continue to receive information about your progress. If you do not wish us to have access to further information about your progress please inform your doctor. If you stop study treatment your doctor will continue to provide you with the best available care.

4. What if there is a problem?

In the event that something goes wrong and you are harmed during this study there are no special compensation arrangements. However, The Medical Research Council, UK (MRC) as the legal sponsor of this study, will give sympathetic consideration to claims for non-negligent harm suffered by a person as a result of a study, or other work supported by MRC. The hospital continues to have a duty of care to you, whether or not you are participating in an MRC supported study. The MRC does not accept liability for any breach in the hospital's duty of care, or any negligence on the part of employees of hospitals. Negligence of NHS staff will be indemnified by the NHS or professional indemnity schemes. If you are harmed due to someone's negligence, then you may have grounds for a legal action but you may have to pay for your legal costs.

If you wish to complain, or have any concerns about any aspect of the way you have been approached or treated during the course of this study, the normal National Health Service complaints mechanisms should be available to you. Participation in this study does not affect your normal rights to complain about any aspect of your treatment and care (contact number details can be obtained from your hospital).

If you have private medical insurance you should consult with your insurer before agreeing to take part.

5. Will my taking part in this study be kept confidential?

All information which is collected about you during the course of the research will be kept strictly confidential. Any information about you which leaves the hospital/surgery will have your name and address removed so that you cannot be recognised from it.

If you agree to join the study, some parts of your medical records and the data collected for the study will be looked at by authorised persons from the MRC. They may also be looked at by representatives from the regulatory authorities to check that the study is being carried out correctly.

We will, if you give permission, contact your GP to notify them of your participation in the trial. We may also, if you give permission, contact other medical practitioners not involved in the research who are otherwise involved with your treatment to notify them of your participation in the trial.

We will ask if we can flag your records with the Office of National Statistics or trace them via the NHS Central Register so that if you move away or decide not to continue with the trial we will still be able to find out how you are doing. You will be asked a question about this on the consent form that you will have to sign before you are entered into the study.

6. What will happen to any samples I give?

Blood and urine samples will be taken at clinic visits to monitor your progress and to check for any side effects as well as for the special tests required in the study protocol. At some visits we will specifically take an extra blood sample to keep in reserve in case future tests are needed. Any leftover blood and urine and these extra blood samples will be stored securely and confidentially using a study number that will be assigned to you, rather than your name or other information that could identify you. As part of the consent we are asking for your permission to store these specimens for a period of 3 years after the study has finished so that we are able to use them for possible other tests in the future. These samples would be gifted by you to the research team. Until the study has been completed we will not know for sure what tests might usefully be done on these specimens. However, future tests would not involve any tests on your genes (DNA). Any future research on these samples taken will require further ethical review.

The researchers do not plan to contact you or your doctor with any results from future studies done on your stored specimens - this is because test are often experimental and should not be used to make decisions on treating your disease. Thus, while you will not benefit directly from any future research done, any results could be used to improve the treatment of HIV and its complications.

If you decide to provide blood for future research but change your mind later, you should inform your clinic that you do not want your samples used in future research. Your samples will then no longer be used.

7. What will happen to the results of the research study?

The results of the study will be published in a medical journal and on the website of the MRC Clinical Trials Unit – www.ctu.mrc.ac.uk. Interim results may be presented at clinical conferences. You will not be identified in any study report or publication.

A summary of the overall study results for patients who have participated will be produced once the study has been completed and analysed. A copy of the final publication will also be available to you through your study doctor.

8. Who is organising and funding the research?

The research is funded by the NHS Health Technology Assessment (HTA) programme, and is sponsored and conducted by the Medical Research Council.

The clinic you attend will be reimbursed for the additional costs incurred to them by your involvement in the study, such as the costs of blood tests and research nurse time. There will be no personal payments made to any member of staff for including you in this study.

9. Who has reviewed the study?

The study has been reviewed by the NHS HTA and the MRC. The study has also been given a favourable ethical opinion for conduct in the NHS by the Cambridgeshire 4 Research Ethics Committee.

10. Contact for Further Information

If you have any further questions concerning the study or if any problems arise during the study, please contact:

……………………………………………………on telephone number…………………………………………..

and 24-hour telephone contact number …………………………………………………………………………….

You may also find it helpful to contact i-Base, an independent information agency. Treatment Information phone line 0808 800 6013 (open Mon-Wed 12-4pm), or website www.i-base.org.uk

Once again, we would like to thank you for taking the time to read this information and for considering taking part in this study.

APPENDIX 2: CONSENT FORM

(To be presented on local-headed paper)
Version 2.0, 18 November 2008

PIVOT: Protease Inhibitor monotherapy Versus Ongoing Triple-therapy in the long-term management of HIV disease

EUDRACT: 2007-006448-23
ISRCTN04857074

Please initial box to agree

1. I confirm that I have read and understand the information sheet dated 18 November 2008 (Version 2.0) for the above study and have had the opportunity to ask questions and discuss it with my doctor. ☐
2. I understand that my participation is voluntary and that I am free to withdraw at any time, without giving any reason, without my medical care or legal rights being affected. ☐
3. I understand that sections of any of my medical notes may be looked at by responsible individuals involved in the running of the study or from regulatory authorities where it is relevant to my taking part in research. I give permission for these individuals to have access to my records. ☐
4. I give permission to be followed up through usual NHS mechanisms (e.g. Office for National Statistics). ☐
5. I agree to take part in PIVOT, the PI monotherapy study. ☐
6. **(Optional)** I agree that any left over blood or urine stored during the study can be used by the research team in future tests. I understand that this will not involve any tests on my genes (DNA). These samples are gifted to the research team. ☐
7. **(Optional)** I agree to my GP being informed of my participation in this study. ☐

Name of Participant	Date	Signature
Name of Person taking consent (if different from researcher)	Date	Signature
Researcher	Date	Signature
Witness (if applicable)	Date	Signature

3 copies: 1 for patient, 1 for researcher, 1 to be kept with hospital notes

APPENDIX 3: GP LETTER

(To be presented on local-headed paper)

[Date]

PIVOT: Protease Inhibitor monotherapy Versus Ongoing Triple-therapy in the long-term management of HIV disease

EUDRACT: 2007-006448-23
ISRCTN04857074

Dear Dr ____________

Your patient, ________________ , has consented to participate in the trial named above and given permission to notify you of their participation in the trial. On _____________ they were randomised to the _________________ arm of the trial.

This is a randomised controlled clinical trial to compare a strategy of switching to boosted protease inhibitor (PI) monotherapy to continuing combination antiretroviral therapy (ART) for long-term management of HIV-infected patients.

This trial aims to determine whether a strategy of switching to PI monotherapy is non-inferior to continuing triple drug therapy in terms of the proportion of patients who maintain all their available drug treatment options after at least 3 years of follow-up.

Please find enclosed a copy of the patient information sheet for this trial and contraindicated medication.

You will be kept up-to-date with your patient's progress but if you have any concerns or questions regarding this study please contact the responsible doctor:

Dr ______________________________ at ___________________________(Hospital)

Tel: ______________________________

Kind regards,

[Name]
[Position]

APPENDIX 4: PARTICIPATING SITES

UK Investigators

Dr Sharmin Obeyesekera<br>Barking Hospital<br>Sydenham Centre<br>Upney Lane<br>Barking		Dr Chloe Orkin<br>Barts & The London NHS Trust<br>Infection & Immunity Clinical Group<br>King George V Block - Andrewes Unit<br>St Bartholomews Hospital<br>West Smithfield	
London	IG11 9LX	London	EC1 7BE
Dr Steve Taylor<br>Birmingham Heartlands Hospital<br>Directorate of Sexual Medicine and HIV<br>Boardsley Green East		Dr Martin Fisher<br>BSUH NHS Trust<br>HIV/GUM Research<br>The Elton John Centre<br>Sussex House<br>1 Abbey Road	
Birmingham	B9 5SS	Brighton	BN2 1ES
Dr Humphrey Birley<br>Cardiff Royal Infirmary<br>Dept. of Genito-Urinary Medicine<br>Newport Road		Dr Hitendra Thaker<br>Castle Hill Hospital<br>Infectious Diseases<br>Castle Hill Road<br>Cottingham	
Cardiff	CF24 0SZ	Hull	HU16 5JQ
Dr Gary Brook<br>Central Middlesex Hospital<br>Patrick Clements Clinic<br>Acton Lane		Prof Brian Gazzard<br>Chelsea and Westminster Hospital<br>4th Floor St Stephen's Centre<br>369 Fulham Road	
London	NW10 7NS	London	SW10 9NH
Dr Nigel O'Farrell<br>Ealing Hospital<br>Infection & Immunity Unit<br>Pasteur Suite<br>Uxbridge Road<br>Southall		Dr Sunda Uthayakumar<br>East and North Hertfordshire NHS Trust<br>Genito-Urinary Medicine<br>Woodlands Clinc<br>Coreys Mill Lane	
Middlesex	UB1 3HW	Stevenage	SG1 4AB
Dr Sinnappah Jebakumar<br>Edith Cavell Hospital<br>Dept. of Sexual Health<br>Clinic E<br>Bretton Gate		Dr Shamela de Silva<br>Farnham Road Hospital<br>Farnham Road<br>Guildford	
Peterborough	PE3 9GZ	Surrey	GU2 7LX
Dr Ray Fox<br>Gartnavel General Hospital<br>The Brownlee Centre<br>Great Western Road		Dr Andrew DeBurgh-Thomas<br>Gloucester Royal Hospital<br>Hope House<br>Dept. of GU & HIV Medicine<br>Great Western Road	
Glasgow	G12 0YN	Gloucester	GL1 3NN

Dr David Chadwick<br>James Cook University Hospital<br>Dept. of Infection & Travel Medicine<br>Marton Road		Dr Frank Post<br>King's College Hospital<br>Dept. of HIV/GUMedicine<br>Weston Education Centre<br>Cutcoombe Road<br>Denmark Hill	
Middlesbrough	TS4 3BW	London	SE5 9RS
Dr Adrian Palfreeman<br>Leicester Royal Infirmary<br>Genito-Urinary Medicine<br>Infirmary Square		Dr Thambiah Balachandran<br>Luton & Dunstable Hospital<br>Dept. of Genito-Urinary Medicine<br>Lewsey Road	
Leicester	LE1 5WW	Luton	LU4 0DZ
Dr Vincent Lee<br>Manchester Royal Infirmary<br>Manchester Centre for Sexual Health<br>Oxford Road		Dr Edmund Ong<br>Newcastle General Hospital<br>Infectious Diseases<br>Westgate Road	
Manchester	M13 9WL	Newcastle upon Tyne	NE4 6BE
Dr Ade Fakoya<br>Newham University Hospital<br>Dept. of Genito-Urinary Medicine<br>Glen Road		Dr Edmund Wilkins<br>North Manchester General Hospital<br>Infectious Diseases<br>Delaunays Road<br>Crumpsall	
London	E13 8SL	Manchester	M8 5RB
Dr Jonathan Ainsworth<br>North Middlesex University Hospital<br>T1 Coleridge Unit<br>Sterling Way<br>Edmonton		Dr Moses Kapembwa<br>Northwick Park & St. Mark's Hospitals<br>Dept. of GU/HIV Medicine<br>Watford Road<br>Harrow	
London	N18 1QX	Middlesex	HA1 3UJ
Dr Brian Angus<br>Oxford Radcliffe Hospitals NHS Trust<br>Nuffield Department of Clinical Medicine<br>Room 5801a, Level 5<br>Headley Way<br>Headington		Dr Fabian Chen<br>Royal Berkshire Hospital<br>Genito-Urinary Medicine<br>London Road<br>Reading	
Oxford	OX3 9DU	Berkshire	RG1 5AN
Dr Elbushra Herieka<br>Royal Bournemouth Hospital<br>Dept. of Genito-Urinary Medicine<br>Castle Lane East		Prof Margaret Johnson<br>Royal Free Hospital<br>Dept. of Infection & Immunity<br>Ian Charleson Centre<br>Ground Floor<br>Pond Street	
Bournemouth	BH7 7DW	London	NW3 2QG
Dr David Dockrell<br>Royal Hallamshire Hospital<br>Infection, Inflammation & Immunity<br>University of Sheffield School of Medicine & Biomedical Sciences<br>L-Floor<br>Glossop Road		Dr Nick Beeching<br>Royal Liverpool University Hospital<br>Tropical & Infectious Disease Unit<br>Prescot Street	
Sheffield	S10 2JF	Liverpool	L7 8XP

Dr Say Pheng Quah
Royal Victoria Hospital
Dept. of Genito-Urinary Medicine
Level 3b Outpatients Centre
Grosvenor Road
Belfast
Northern Ireland BT12 6BA

Dr John Day
Southend Hospital
Infectious Diseases & General Medicine
Prittlewell Chase
Westcliff on Sea
Essex SS0 0RY

Dr Mark Gompels
Southmead Hospital
Dept. of Immunology
Westbury-on-Trym
Bristol BS10 5NB

Dr Phillip Hay
St George's Hospital
Clinical Infection Unit
Jenner Wing
Blackshaw Road
Tooting
London SW17 0QT

Dr Alan Winston
St Mary's Hospital, London
Clinical Trials Centre
Winston Churchill Wing
Praed Street
London W2 1NY

Dr Veerakathy Harindra
St Mary's Hospital, Portsmouth
Dept. of Genti-Urinary Medicine
Milton Road
Portsmouth PO3 6AD

Dr Ian Williams
The Mortimer Market Centre, UCH
Genito-Urinary Medicine
University College Hospital
The Mortimer Market Centre
Off Capper Street
London WC1E 6AU

Dr Sris Allan
University Hospital of Coventry & Warwickshire
Dept. of Genito-Urinary Medicine
Clifford Bridge Road
Walsgrave
Coventry CV2 2DX

Dr Joseph Arumainayagam
Walsall Manor Hospital
Dept. of Genito-Urinary Medicine
Moat Road
Walsall WS2 9PS

Professor Charles Lacey
Hull York Medical School
University of York
York YO10 5DD

Dr Iain Reeves
Homerton University Hospital
Homerton Row
London E9 6SR

Dr Jane Minton
St James's University Hospital
Beckett Street
Leeds LS9 7TF

Ireland Investigators

Dr Patrick Mallon
Mater Misericordiae University Hospital
UCD School of Medicine & Medical Sciences
Catherine McAuley Centre
Nelson Street
Dublin 7

Prof Fiona Mulchay
St. James' Hospital
Guide Clinic
Hospital 5
Dublin 8

APPENDIX 5: EQ-5D QUESTIONNAIRE

Describing your own health today

By placing a tick in one box in each group below, please indicate which statements best describe your own health state today.

Mobility

- I have no problems in walking about ❑
- I have some problems in walking about ❑
- I am confined to bed ❑

Self-Care

- I have no problems with self-care ❑
- I have some problems washing or dressing myself ❑
- I am unable to wash or dress myself ❑

Usual Activities (e.g. work, study, housework, family or leisure activities)

- I have no problems with performing my usual activities ❑
- I have some problems with performing my usual activities ❑
- I am unable to perform my usual activities ❑

Pain/Discomfort

- I have no pain or discomfort ❑
- I have moderate pain or discomfort ❑
- I have extreme pain or discomfort ❑

Anxiety/Depression

- I am not anxious or depressed ❑
- I am moderately anxious or depressed ❑
- I am extremely anxious or depressed ❑

APPENDIX 6: MOS-HIV QUESTIONNAIRE

MOS – HIV QUALITY OF LIFE QUESTIONNAIRE

Date :	Clinic no:	Initials:	Date of birth:	Trial no:

INSTRUCTIONS

1. Please answer every question even though some questions may seem very similar to others.
2. Answer by placing a tick in the appropriate box. If you feel that the answer lies in between one possible response and another, please mark whichever box comes closest to the way you feel.
3. If you don't understand what a question means, please ask the Trial Nurse to explain or clarify it.

THANK YOU FOR COMPLETING THIS QUESTIONNAIRE YOUR COOPERATION WILL BENEFIT YOURSELF, OTHER PEOPLE AND RESEARCH INTO HIV DISEASE.

1. In general, would you say your health is: (Please tick ONE box)

☐ Excellent ☐ Very Good ☐ Good ☐ Fair ☐ Poor

2. How much **bodily** pain have you generally had during **the past 4 weeks?** (Please tick ONE box)

☐ None ☐ Very Mild ☐ Mild ☐ Moderate ☐ Severe ☐ Very Severe

3. During **the past 4 weeks**, how much did **pain** interfere with your normal work (including both work outside the home and housework)? (Please tick ONE box)

☐ Not at all ☐ A little bit ☐ Moderately ☐ Quite a bit ☐ Extremely

4. The following questions are about activities you might do during a typical day. Does your health now limit you in these activities? If so, how much? (Please tick ONE box on each line)

	**YES, Limited A Lot**	**YES, Limited A Little**	**NO, Not Limited At All**
a. The kinds or amounts of **vigorous** activities you can do, like lifting heavy objects, running or participating in strenuous sports.	☐	☐	☐
b. The kinds or amounts of **moderate** activities you can do, like moving a table, carrying groceries or bowling.	☐	☐	☐
c. Walking uphill or climbing a few flights of stairs.	☐	☐	☐
d. Bending, lifting or stooping.	☐	☐	☐
e. Walking one hundred yards.	☐	☐	☐
f. Easting, dressing, bathing, or using the toilet.	☐	☐	☐

5. Does your health **keep** you from working at a job, doing work around the house or going to school? (Please tick ONE box)

☐ Yes ☐ No

6. Have you been unable to do **certain kinds or amounts** of work, housework, or schoolwork because of your health? (Please tick ONE box)

☐ Yes ☐ No

For each of the following questions, please tick the box for the one answer that comes closest to the way you have been feeling during the past 4 weeks. (Please tick ONE box on each line)

	All of the Time	Most of the Time	A Good Bit of the Time	Some of the Time	A Little of the Time	None of the Time
7. How much of the time, during the past 4 weeks, has your **health limited your social activities** (like visiting with friends or close relatives)?	☐	☐	☐	☐	☐	☐
8. How much of the time, during the past 4 weeks:	☐	☐	☐	☐	☐	☐
a. Have you been a very nervous person?	☐	☐	☐	☐	☐	☐
b. Have you felt calm and peaceful?	☐	☐	☐	☐	☐	☐
c. Have you felt downhearted and low?	☐	☐	☐	☐	☐	☐
d. Have you been a happy person?	☐	☐	☐	☐	☐	☐
e. Have you felt so down in the dumps that nothing could cheer you up?	☐	☐	☐	☐	☐	☐
**9. How often during the past four weeks:**						
a. Did you feel full of life?	☐	☐	☐	☐	☐	☐
b. Did you feel worn out?	☐	☐	☐	☐	☐	☐
c. Did you feel tired?	☐	☐	☐	☐	☐	☐
d. Did you have enough energy to do the things you wanted to do?	☐	☐	☐	☐	☐	☐
e. Did you feel weighed down by your health problems?	☐	☐	☐	☐	☐	☐
f. Were you discouraged by your health problems?	☐	☐	☐	☐	☐	☐
g. Did you feel despair over your health problems?	☐	☐	☐	☐	☐	☐
h. Were you afraid because of your health?	☐	☐	☐	☐	☐	☐
10. How much of the time, during the **past 4 weeks:**						
a. Did you have difficulty reasoning and solving problems, for example, making plans, making decisions, learning new things?	☐	☐	☐	☐	☐	☐
b. Did you forget things that happened recently, for example, where you put things and when you had appointments?	☐	☐	☐	☐	☐	☐
c. Did you have trouble keeping your attention on any activity for long?	☐	☐	☐	☐	☐	☐
d. Did you have difficulty doing activities involving concentration and thinking?	☐	☐	☐	☐	☐	☐

(Please tick ONE box on each line)

	Definitely True	Mostly True	Don't Know	Mostly False	Definitely False
11. Please tick the box that best describes whether each of the following statements is true or false for you.	☐	☐	☐	☐	☐
a. I am somewhat ill.	☐	☐	☐	☐	☐
b. I am as healthy as anybody I know.	☐	☐	☐	☐	☐
c. My health is excellent.	☐	☐	☐	☐	☐
d. I have been feeling bad lately.	☐	☐	☐	☐	☐

12. How has the quality of your life been during the **past 4 weeks**? That is, how have things been going for you?
(Please tick ONE box)

- Very well; could hardly be better ☐
- Pretty good ☐
- Good and bad parts about equal ☐
- Pretty bad ☐
- Very bad; could hardly be worse ☐

13. How would you rate your physical health and emotional condition now compared to **4 weeks ago**?
(Please tick ONE box)

- Much better ☐
- A little better ☐
- About the same ☐
- A little worse ☐
- Much worse ☐

APPENDIX 7: TOXICITY TABLE

Division of AIDS table for Grading the Severity of Adult and Pediatric adverse events. Publish date: December 2004.

General Instructions:
If the need arises to grade a clinical adverse event (AE) that is not identified in the DAIDS AE grading table, us the category "Estimating Severity Grade" located at the top of the table.

If the severity of an AE could fall under either one of two grades (e.g. the severity of an AE could be either Grade 2 or Grade 3) select the higher of the two grades for the AE.

Definitions:

Basic Self-care Functions — Adult: Activities such as bathing, dressing, toileting, transfer/movement, continence, and feeding.

Usual Social & Functional Activities — Adult: Adaptive tasks and desirable activities, such as going to work, shopping, cooking, use of transportation, pursuing a hobby etc.

CLINICAL				
**PARAMETER**	**GRADE 1 MILD**	**GRADE 2 MODERATE**	**GRADE 3 SEVERE**	**GRADE 4 POTENTIALLY LIFE-THREATENING**
**ESTIMATING SEVERITY**				
Clinical adverse event NOT identified elsewhere in this DAIDS AE grading table	Symptoms causing no or minimal interference with usual social & functional activities	Symptoms causing greater than minimal interference with usual social & functional activities	Symptoms causing inability to perform usual social & functional activities	Symptoms causing inability to perform basic self-care functions OR medical or operative intervention indicated to prevent permanent impairment, persistent disability, or death
**SYSTEMIC**				
Acute systemic allergic reaction	Localized urticaria (wheals) with no medical intervention indicated	Localized urticaria with medical intervention indicated OR mild angioedema with no medical intervention indicated	Generalized urticaria OR angioedema with medical intervention indicated OR symptomatic mild bronchospasm	Acute anaphylaxis OR Life-threatening bronchospasm OR laryngeal edema
Chills	Symptoms causing no or minimal interference with usual social & functional activities	Symptoms causing greater than minimal interference with usual social & functional activities	Symptoms causing inability to perform usual social & functional activities	NA
Fatigue<br><br>Malaise	Symptoms causing no or minimal interference with usual social & functional activities	Symptoms causing greater than minimal interference with usual social & functional activities	Symptoms causing inability to perform usual social & functional activities	Incapacitating fatigue/ malaise symptoms causing inability to perform basic self-care functions
Fever (nonaxillary)	37.7 – 38.6°C	38.7 – 39.3°C	39.4 – 40.5°C	> 40.5°C
Pain (indicate body site)<br><br>DO NOT use for pain due to injection (See Injection Site Reactions: Injection site pain) See also Headache, Arthralgia, and Myalgia	Pain causing no or minimal interference with usual social & functional activities	Pain causing greater than minimal interference with usual social & functional activities	Pain causing inability to perform usual social & functional activities	Disabling pain causing inability to perform basic self-care functions OR hospitalization (other than emergency room visit) indicated
Unintentional weight loss	NA	5 – 9% loss in body weight from baseline	10 – 19% loss in body weight from baseline	≥ 20% loss in body weight from baseline OR aggressive intervention indicated [e.g., tube feeding or total parenteral nutrition (TPN)]

CLINICAL				
**PARAMETER**	**GRADE 1<br>MILD**	**GRADE 2<br>MODERATE**	**GRADE 3<br>SEVERE**	**GRADE 4<br>POTENTIALLY<br>LIFE-THREATENING**
**INFECTION**				
Infection (any other than HIV infection)	Localized, no systemic antimicrobial treatment indicated AND Symptoms causing no or minimal interference with usual social & functional activities	Systemic antimicrobial treatment indicated OR Symptoms causing greater than minimal interference with usual social & functional activities	Systemic anti-microbial treatment indicated AND Symptoms causing inability to perform usual social & functional activities OR Operative intervention (other than simple incision and drainage) indicated	Life-threatening consequences (e.g., septic shock)
**INJECTION SITE REACTIONS**				
Injection site pain (pain without touching)<br><br>Or<br><br>Tenderness (pain when area is touched)	Pain/tenderness causing no or minimal limitation of use of limb	Pain/tenderness limiting use of limb OR Pain/tenderness causing greater than minimal interference with usual social & functional activities	Pain/tenderness causing inability to perform usual social & functional activities	Pain/tenderness causing inability to perform basic self-care function OR Hospitalization (other than emergency room visit) indicated for management of pain/tenderness
Injection site reaction (localized)				
**Adult<br>> 15 years**	Erythema OR Induration of 5x5 cm – 9x9 cm (or 25 cm2 – 81cm2)	Erythema OR Induration OR Edema > 9 cm any diameter (or > 81 cm2)	Ulceration OR Secondary infection OR Phlebitis OR Sterile abscess OR Drainage	Necrosis (involving dermis and deeper tissue)
**Pediatric<br>≤ 15 years**	Erythema OR Induration OR Edema present but ≤ 2.5 cm diameter	Erythema OR Induration OR Edema > 2.5 cm diameter but < 50% surface area of the extremity segment (e.g., upper arm/thigh)	Erythema OR Induration OR Edema involving ≥ 50% surface area of the extremity segment (e.g. upper arm/thigh) OR Ulceration OR Secondary infection OR Phlebitis OR Sterile abscess OR Drainage	Necrosis (involving dermis and deeper tissue)
Pruritis associated with injection<br><br>See also Skin: Pruritis (itching - no skin lesions)	Itching localized to injection site AND Relieved spontaneously or with < 48 hours treatment	Itching beyond the injection site but not generalized OR Itching localized to injection site requiring ≥ 48 hours treatment	Generalized itching causing inability to perform usual social & functional activities	NA
**SKIN – DERMATOLOGICAL**				
Alopecia	Thinning detectable by study participant (or by caregiver for young children and disabled adults)	Thinning or patchy hair loss detectable by health care provider	Complete hair loss	NA
Cutaneous reaction – rash	Localized macular rash	Diffuse macular, maculopapular, or morbilliform rash OR Target lesions	Diffuse macular, maculopapular, or morbilliform rash with vesicles or limited number of bullae OR Superficial ulcerations of mucous membrane limited to one site	Extensive or generalized bullous lesions OR Stevens-Johnson syndrome OR Ulceration of mucous membrane involving two or more distinct mucosal sites OR Toxic epidermal necrolysis (TEN)
Hyperpigmentation	Slight or localized	Marked or generalized	NA	NA
Hypopigmentation	Slight or localized	Marked or generalized	NA	NA
Pruritis (itching – no skin lesions)<br><br>(See also Injection Site Reactions: Pruritis associated with injection)	Itching causing no or minimal interference with usual social & functional activities	Itching causing greater than minimal interference with usual social & functional activities	Itching causing inability to perform usual social & functional activities	NA

CLINICAL				
**PARAMETER**	**GRADE 1<br>MILD**	**GRADE 2<br>MODERATE**	**GRADE 3<br>SEVERE**	**GRADE 4<br>POTENTIALLY<br>LIFE-THREATENING**
**CARDIOVASCULAR**				
Cardiac arrhythmia (general)<br>(By ECG or physical exam)	Asymptomatic AND No intervention indicated	Asymptomatic AND Non-urgent medical intervention indicated	Symptomatic, non-lifethreatening AND Non-urgent Medical intervention indicated	Life-threatening arrhythmia OR Urgent intervention indicated
Cardiacischemia/ infarction	NA	NA	Symptomatic ischemia (stable angina) OR Testing consistent with ischemia	Unstable angina OR Acute myocardial infarction
Hemorrhage<br>(significant acute blood loss)	NA	Symptomatic AND No transfusion indicated	Symptomatic AND Transfusion of ≤ 2 units packed RBCs (for children ≤ 10 cc/kg) indicated	Life-threatening hypotension OR Transfusion of > 2 units packed RBCs (for children > 10 cc/kg) indicated
Hypertension				
**Adult**<br>**> 17 years**<br>(with repeat testing at same visit)	> 140 – 159 mmHg systolic<br>OR<br>> 90 – 99 mmHg diastolic	> 160 – 179 mmHg systolic<br>OR<br>> 100 – 109 mmHg diastolic	> 180 mmHg systolic<br>OR<br>> 110 mmHg diastolic	Life-threatening consequences (e.g., malignant hypertension) OR Hospitalization indicated (other than emergency room visit)
**Pediatric**<br>**≤ 17 years**<br>(with repeat testing at same visit)	NA	91st – 94th percentile adjusted for age, height, and gender (systolic and/or diastolic)	≥ 95th percentile adjusted for age, height, and gender (systolic and/or diastolic)	Life-threatening consequences (e.g., malignant hypertension) OR Hospitalization indicated (other than emergency room visit)
Hypotension	NA	Symptomatic, corrected with oral fluid replacement	Symptomatic, IV fluids indicated	Shock requiring use of vasopressors or mechanical assistance to maintain blood pressure
Pericardial effusion	Asymptomatic, small effusion requiring no intervention	Asymptomatic, moderate or larger effusion requiring no intervention	Effusion with non-life threatening physiologic consequences OR Effusion with non-urgent intervention indicated	Life-threatening consequences (e.g., tamponade) OR Urgent intervention indicated
Prolonged PR interval				
**Adult**<br>**> 16 years**	PR interval<br>0.21 – 0.25 sec	PR interval<br>> 0.25 sec	Type II 2nd degree AV block OR Ventricular pause > 3.0 sec	Complete AV block
**Pediatric**<br>**≤ 16 years**	1st degree AV block (PR > normal for age and rate)	Type I 2nd degree AV block	Type II 2nd degree AV block	Complete AV block
Prolonged QTc				
**Adult**<br>**> 16 years**	Asymptomatic, QTc interval 0.45 – 0.47 sec OR Increase interval < 0.03 sec above baseline	Asymptomatic, QTc interval 0.48 – 0.49 sec OR Increase in interval 0.03 – 0.05 sec above baseline	Asymptomatic, QTc interval ≥ 0.50 sec OR Increase in interval ≥ 0.06 sec above baseline	Life-threatening consequences, e.g. Torsade de pointes or other associated serious ventricular dysrhythmia
**Pediatric**<br>**≤ 16 years**	Asymptomatic, QTc interval 0.450 – 0.464 sec	Asymptomatic, QTc interval 0.465 – 0.479 sec	Asymptomatic, QTc interval ≥ 0.480 sec	Life-threatening consequences, e.g. Torsade de pointes or other associated serious ventricular dysrhythmia
Thrombosis/embolism	NA	Deep vein thrombosis AND No intervention indicated (e.g., anticoagulation, lysis filter, invasive procedure)	Deep vein thrombosis AND intervention indicated (e.g., anticoagulation, lysis filter, invasive procedure)	Embolic event (e.g., pulmonary embolism, life-threatening thrombus)
Vasovagal episode (associated with a procedure of any kind)	Present without loss of consciousness	Present with transient loss of consciousness	NA	NA
Ventricular dysfunction (congestive heart failure)	NA	Asymptomatic diagnostic finding AND intervention indicated	New onset with symptoms OR Worsening symptomatic congestive heart failure	Life-threatening congestive heart failure

CLINICAL				
**PARAMETER**	**GRADE 1 MILD**	**GRADE 2 MODERATE**	**GRADE 3 SEVERE**	**GRADE 4 POTENTIALLY LIFE-THREATENING**
**GASTROINTESTINAL**				
Anorexia	Loss of appetite without decreased oral intake	Loss of appetite associated with decreased oral intake without significant weight loss	Loss of appetite associated with significant weight loss	Life-threatening consequences OR Aggressive intervention indicated [e.g., tube feeding or total parenteral nutrition TPN)]
Ascites	Asymptomatic	Symptomatic AND Intervention indicated (e.g., diuretics or Therapeutic paracentesis)	Symptomatic despite intervention	Life-threatening consequences
Cholecystitis	NA	Symptomatic AND Medical intervention indicated	Radiologic, endoscopic or operative intervention indicated	Life-threatening consequences (e.g., sepsis or perforation)
Constipation	NA	Persistent constipation requiring regular use of dietary modifications, laxatives, or enemas	Obstipation with manual evacuation indicated	Life-threatening consequences (e.g., obstruction)
Diarrhea				
**Adult and Pediatric ≥ 1 year**	Transient or intermittent episodes of unformed stools OR Increase of ≤ 3 stools over baseline per 24-hour period	Persistent episodes of unformed to watery stools OR Increase of 4 – 6 stools over baseline per 24-hour period	Bloody diarrhea OR Increase of ≥ 7 stools per 24-hour period OR IV fluid replacement indicated	Life-threatening consequences (e.g., hypotensive shock)
**Pediatric < 1 year**	Liquid stools (more unformed than usual) but usual number of stools	Liquid stools with increased number of stools OR Mild dehydration	Liquid stools with moderate dehydration	Liquid stools resulting in severe dehydration with aggressive rehydration indicated OR Hypotensive shock
Dysphagia-Odynophagia	Symptomatic but able to eat usual diet	Symptoms causing altered dietary intake without medical intervention indicated	Symptoms causing severely altered dietary intake with medical intervention indicated	Life-threatening reduction in oral intake
Mucositis/stomatitis (clinical exam)<br><br>Indicate site (e.g., larynx, oral)<br>See Genitourinary for Vulvovaginitis<br>See also Dysphagia-Odynophagia and Proctitis	Erythema of the mucosa	Patchy pseudomembranes or ulcerations	Confluent pseudomembranes or ulcerations OR Mucosal bleeding with minor trauma	Tissue necrosis OR Diffuse spontaneous mucosal bleeding OR Life-threatening consequences (e.g., aspiration, choking)
Nausea	Transient (< 24 hours) or intermittent nausea with no or minimal interference with oral intake	Persistent nausea resulting in decreased oral intake for 24 – 48 hours	Persistent nausea resulting in minimal oral intake for > 48 hours OR Aggressive rehydration indicated (e.g., IV fluids)	Life-threatening consequences (e.g., hypotensive shock)
Pancreatitis	NA	Symptomatic AND Hospitalization not indicated (other than emergency room visit)	Symptomatic AND Hospitalization indicated (other than emergency room visit)	Life-threatening consequences (e.g., circulatory failure, hemorrhage, sepsis)
Proctitis (functionalsymptomatic)<br><br>Also see Mucositis/stomatitis for clinical exam	Rectal discomfort AND No intervention indicated	Symptoms causing greater than minimal interference with usual social & functional activities OR Medical intervention indicated	Symptoms causing inability to perform usual social & functional activities OR Operative intervention indicated	Life-threatening consequences (e.g., perforation)
Vomiting	Transient or intermittent vomiting with no or minimal interference with oral intake	Frequent episodes of vomiting with no or mild dehydration	Persistent vomiting resulting in orthostatic hypotension OR Aggressive rehydration indicated (e.g., IV fluids)	Life-threatening consequences (e.g., hypotensive shock)

CLINICAL				
**PARAMETER**	**GRADE 1<br>MILD**	**GRADE 2<br>MODERATE**	**GRADE 3<br>SEVERE**	**GRADE 4<br>POTENTIALLY LIFE-THREATENING**
**NEUROLOGIC**				
Alteration in personality-behavior or in mood (e.g., agitation, anxiety, depression, mania, psychosis)	Alteration causing no or minimal interference with usual social & functional activities	Alteration causing greater than minimal interference with usual social & functional activities	Alteration causing inability to perform usual social & functional activities	Behavior potentially harmful to self or others (e.g., suicidal and homicidal ideation or attempt, acute psychosis) OR Causing inability to perform basic self-care functions
Altered Mental Status<br><br>For Dementia, see Cognitive and behavioral/attentional disturbance (including dementia and attention deficit disorder)	Changes causing no or minimal interference with usual social & functional activities	Mild lethargy or somnolence causing greater than minimal interference with usual social & functional activities	Confusion, memory impairment, lethargy, or somnolence causing inability to perform usual social & functional activities	Delirium OR obtundation, OR coma
Ataxia	Asymptomatic ataxia detectable on exam OR Minimal ataxia causing no or minimal interference with usual social & functional activities	Symptomatic ataxia causing greater than minimal interference with usual social & functional activities	Symptomatic ataxia causing inability to perform usual social & functional activities	Disabling ataxia causing inability to perform basic self-care functions
Cognitive and behavioral/attentional disturbance (including dementia and attention deficit disorder)	Disability causing no or minimal interference with usual social & functional activities OR Specialized resources not indicated	Disability causing greater than minimal interference with usual social & functional activities OR Specialized resources on part-time basis indicated	Disability causing inability to perform usual social & functional activities OR Specialized resources on a full-time basis indicated	Disability causing inability to perform basic self-care functions OR Institutionalization indicated
CNS ischemia (acute)	NA	NA	Transient ischemic attack	Cerebral vascular accident (CVA, stroke) with neurological deficit
Developmental delay – **Pediatric ≤ 16 years**	Mild developmental delay, either motor or cognitive, as determined by comparison with a developmental screening tool appropriate for the setting	Moderate developmental delay, either motor or cognitive, as determined by comparison with a developmental screening tool appropriate for the setting	Severe developmental delay, either motor or cognitive, as determined by comparison with a developmental screening tool appropriate for the setting	Developmental regression, either motor or cognitive, as determined by comparison with a developmental screening tool appropriate for the setting
Headache	Symptoms causing no or minimal interference with usual social & functional activities	Symptoms causing greater than minimal interference with usual social & functional activities	Symptoms causing inability to perform usual social & functional activities	Symptoms causing inability to perform basic self-care functions OR Hospitalization indicated (other than emergency room visit) OR Headache with significant impairment of alertness or other neurologic function
Insomnia	NA	Difficulty sleeping causing greater than minimal interference with usual social & functional activities	Difficulty sleeping causing inability to perform usual social & functional activities	Disabling insomnia causing inability to perform basic self-care functions
Neuromuscular weakness (including myopathy & neuropathy)	Asymptomatic with decreased strength on exam OR Minimal muscle weakness causing no or minimal interference with usual social & functional activities	Muscle weakness causing greater than minimal interference with usual social & functional activities	Muscle weakness causing inability to perform usual social & functional activities	Disabling muscle weakness causing inability to perform basic self-care functions OR Respiratory muscle weakness impairing ventilation

CLINICAL				
**PARAMETER**	**GRADE 1<br>MILD**	**GRADE 2<br>MODERATE**	**GRADE 3<br>SEVERE**	**GRADE 4<br>POTENTIALLY<br>LIFE-THREATENING**
Neurosensory alteration (including paresthesia and painful neuropathy)	Asymptomatic with sensory alteration on exam or minimal paresthesia causing no or minimal interference with usual social & functional activities	Sensory alteration or paresthesia causing greater than minimal interference with usual social & functional activities	Sensory alteration or paresthesia causing inability to perform usual social & functional activities	Disabling sensory alteration or paresthesia causing inability to perform basic self-care functions
Seizure: (new onset)<br>**– Adult ≥ 18 years**<br><br>See also Seizure: (known pre-existing seizure disorder)	NA	1 seizure	2 – 4 seizures	Seizures of any kind which are prolonged, repetitive (e.g., status epilepticus), or difficult to control (e.g., refractory epilepsy)
Seizure: (known pre-existing seizure disorder)<br>**– Adult ≥ 18 years**<br><br>For worsening of existing epilepsy the grades should be based on an increase from previous level of control to any of these levels.	NA	Increased frequency of pre-existing seizures (non-repetitive) without change in seizure character OR Infrequent breakthrough seizures while on stable medication in a previously controlled seizure disorder	Change in seizure character from baseline either in duration or quality (e.g., severity or focality)	Seizures of any kind which are prolonged, repetitive (e.g., status epilepticus), or difficult to control (e.g., refractory epilepsy)
Seizure<br>**– Pediatric < 18 years**	Seizure, generalized onset with or without secondary generalization, lasting < 5 minutes with < 24 hours post ictal state	Seizure, generalized onset with or without secondary generalization, lasting 5 – 20 minutes with < 24 hours post ictal state	Seizure, generalized onset with or without secondary generalization, lasting > 20 minutes	Seizure, generalized onset with or without secondary generalization, requiring intubation and sedation
Syncope (not associated with a procedure)	NA	Present	NA	NA
Vertigo	Vertigo causing no or minimal interference with usual social & functional activities	Vertigo causing greater than minimal interference with usual social & functional activities	Vertigo causing inability to perform usual social & functional activities	Disabling vertigo causing inability to perform basic self-care functions
**RESPIRATORY**				
Bronchospasm (acute)	FEV1 or peak flow reduced to 70 – 80%	FEV1 or peak flow 50 – 69%	FEV1 or peak flow 25 – 49%	Cyanosis OR FEV1 or peak flow < 25% OR Intubation
Dyspnea or respiratory distress				
**Adult ≥ 14 years**	Dyspnea on exertion with no or minimal interference with usual social & functional activities	Dyspnea on exertion causing greater than minimal interference with usual social & functional activities	Dyspnea at rest causing inability to perform usual social & functional activities	Respiratory failure with ventilatory support indicated
**Pediatric < 14 years**	Wheezing OR minimal increase in respiratory rate for age	Nasal flaring OR Intercostal retractions OR Pulse oximetry 90 – 95%	Dyspnea at rest causing inability to perform usual social & functional activities OR Pulse oximetry < 90%	Respiratory failure with ventilatory support indicated
**MUSCULOSKELETAL**				
Arthralgia<br><br>See also Arthritis	Joint pain causing no or minimal interference with usual social & functional activities	Joint pain causing greater than minimal interference with usual social & functional activities	Joint pain causing inability to perform usual social & functional activities	Disabling joint pain causing inability to perform basic self-care functions
Arthritis<br><br>See also Arthralgia	Stiffness or joint swelling causing no or minimal interference with usual social & functional activities	Stiffness or joint swelling causing greater than minimal interference with usual social & functional activities	Stiffness or joint swelling causing inability to perform usual social & functional activities	Disabling joint stiffness or swelling causing inability to perform basic self-care functions

CLINICAL				
**PARAMETER**	**GRADE 1 MILD**	**GRADE 2 MODERATE**	**GRADE 3 SEVERE**	**GRADE 4 POTENTIALLY LIFE-THREATENING**
Bone Mineral Loss				
**Adult ≥ 21 years**	BMD t-score -2.5 to -1.0	BMD t-score < -2.5	Pathological fracture (including loss of vertebral height)	Pathologic fracture causing life-threatening consequences
**Pediatric < 21 years**	BMD z-score -2.5 to -1.0	BMD z-score < -2.5	Pathological fracture (including loss of vertebral height)	Pathologic fracture causing life-threatening consequences
Myalgia (non-injection site)	Muscle pain causing no or minimal interference with usual social & functional activities	Muscle pain causing greater than minimal interference with usual social & functional activities	Muscle pain causing inability to perform usual social & functional activities	Disabling muscle pain causing inability to perform basic self-care functions
Osteonecrosis	NA	Asymptomatic with radiographic findings AND No operative intervention indicated	Symptomatic bone pain with radiographic findings OR Operative intervention indicated	Disabling bone pain with radiographic findings causing inability to perform basic self-care functions
**GENITOURINARY**				
Cervicitis (symptoms)<br><br>(For use in studies evaluating topical study agents)<br><br>For other cervicitis see Infection: Infection (any other than HIV infection)	Symptoms causing no or minimal interference with usual social & functional activities	Symptoms causing greater than minimal interference with usual social & functional activities	Symptoms causing inability to perform usual social & functional activities	Symptoms causing inability to perform basic self-care functions
Cervicitis (clinical exam)<br><br>(For use in studies evaluating topical study agents)<br><br>For other cervicitis see Infection: Infection (any other than HIV infection)	Minimal cervical abnormalities on examination (erythema, mucopurulent discharge, or friability) OR Epithelial disruption < 25% of total surface	Moderate cervical abnormalities on examination (erythema, mucopurulent discharge, or friability) OR Epithelial disruption of 25 – 49% total surface	Severe cervical abnormalities on examination (erythema, mucopurulent discharge, or friability) OR Epithelial disruption 50 – 75% total surface	Epithelial disruption > 75% total surface
Inter-menstrual bleeding (IMB)	Spotting observed by participant OR Minimal blood observed during clinical or colposcopic examination	Inter-menstrual bleeding not greater in duration or amount than usual menstrual cycle	Inter-menstrual bleeding greater in duration or amount than usual menstrual cycle	Hemorrhage with lifethreatening hypotension OR Operative intervention indicated
Urinary tract obstruction (e.g., stone)	NA	Signs or symptoms of urinary tract obstruction without hydronephrosis or renal dysfunction	Signs or symptoms of urinary tract obstruction with hydronephrosis or renal dysfunction	Obstruction causing life-threatening consequences
Vulvovaginitis (symptoms)<br><br>(Use in studies evaluating topical study agents)<br><br>For other vulvovaginitis see Infection: Infection (any other than HIV infection)	Symptoms causing no or minimal interference with usual social & functional activities	Symptoms causing greater than minimal interference with usual social & functional activities	Symptoms causing inability to perform usual social & functional activities	Symptoms causing inability to perform basic self-care functions
Vulvovaginitis (clinical exam)<br><br>(Use in studies evaluating topical study agents)<br><br>For other vulvovaginitis see Infection: Infection (any other than HIV infection)	Minimal vaginal abnormalities on examination OR Epithelial disruption < 25% of total surface	Moderate vaginal abnormalities on examination OR Epithelial disruption of 25 - 49% total surface	Severe vaginal abnormalities on examination OR Epithelial disruption 50 - 75% total surface	Vaginal perforation OR Epithelial disruption > 75% total surface

CLINICAL				
**PARAMETER**	**GRADE 1 MILD**	**GRADE 2 MODERATE**	**GRADE 3 SEVERE**	**GRADE 4 POTENTIALLY LIFE-THREATENING**
**OCULAR/VISUAL**				
Uveitis	Asymptomatic but detectable on exam	Symptomatic anterior uveitis OR Medical intervention indicated	Posterior or pan-uveitis OR Operative intervention indicated	Disabling visual loss in affected eye(s)
Visual changes (from baseline)	Visual changes causing no or minimal interference with usual social & functional activities	Visual changes causing greater than minimal interference with usual social & functional activities	Visual changes causing inability to perform usual social & functional activities	Disabling visual loss in affected eye(s)
**ENDOCRINE/METABOLIC**				
Abnormal fat accumulation (e.g., back of neck, breasts, abdomen)	Detectable by study participant (or by caregiver for young children and disabled adults)	Detectable on physical exam by health care provider	Disfiguring OR Obvious changes on casual visual inspection	NA
Diabetes mellitus	NA	New onset without need to initiate medication OR Modification of current medications to regain glucose control	New onset with initiation of medication indicated OR Diabetes uncontrolled despite treatment modification	Life-threatening consequences (e.g., ketoacidosis, hyperosmolar nonketotic coma)
Gynecomastia	Detectable by study participant or caregiver (for young children and disabled adults)	Detectable on physical exam by health care provider	Disfiguring OR Obvious on casual visual inspection	NA
Hyperthyroidism	Asymptomatic	Symptomatic causing greater than minimal interference with usual social & functional activities OR Thyroid suppression therapy indicated	Symptoms causing inability to perform usual social & functional activities OR Uncontrolled despite treatment modification	Life-threatening consequences (e.g., thyroid storm)
Hypothyroidism	Asymptomatic	Symptomatic causing greater than minimal interference with usual social & functional activities OR Thyroid replacement therapy indicated	Symptoms causing inability to perform usual social & functional activities OR Uncontrolled despite treatment modification	Life-threatening consequences (e.g., myxedema coma)
Lipoatrophy (e.g., fat loss from the face, extremities, buttocks)	Detectable by study participant (or by caregiver for young children and disabled adults)	Detectable on physical exam by health care provider	Disfiguring OR Obvious on casual visual inspection	NA

LABORATORY				
**PARAMETER**	**GRADE 1 MILD**	**GRADE 2 MODERATE**	**GRADE 3 SEVERE**	**GRADE 4 POTENTIALLY LIFE-THREATENING**
**HEMATOLOGY**	***Standard International Units are listed in italics***			
Absolute CD4+ count **– Adult and Pediatric > 13 years** (HIV NEGATIVE ONLY)	300 – 400/mm^3 *300 – 400/µL*	200 – 299/mm^3 *200 – 299/µL*	100 – 199/mm^3 *100 – 199/µL*	< 100/mm^3 *< 100/µL*
Absolute lymphocyte count **– Adult and Pediatric > 13 years** (HIV NEGATIVE ONLY)	600 – 650/mm^3 *0.600 x 10^9 – 0.650 x 10^9/L*	500 – 599/mm^3 *0.500 x 10^9 – 0.599 x 10^9/L*	350 – 499/mm^3 *0.350 x 10^9 – 0.499 x 10^9/L*	< 350/mm^3 *< 0.350 x 10^9/L*
Absolute neutrophil count (ANC)				
**Adult and Pediatric, > 7 days**	1,000 – 1,300/mm^3 *1.000 x 10^9 – 1.300 x 10^9/L*	750 – 999/mm^3 *0.750 x 10^9 – 0.999 x 10^9/L*	500 – 749/mm^3 *0.500 x 10^9 – 0.749 x 10^9/L*	< 500/mm^3 *< 0.500 x 10^9/L*

LABORATORY				
**PARAMETER**	**GRADE 1 MILD**	**GRADE 2 MODERATE**	**GRADE 3 SEVERE**	**GRADE 4 POTENTIALLY LIFE-THREATENING**
**Infant□†, 2 – ≤ 7 days**	1,250 – 1,500/mm^3<br>*1.250 x 10^9 – 1.500 x 10^9/L*	1,000 – 1,249/mm^3<br>*1.000 x 10^9 – 1.249 x 10^9/L*	750 – 999/mm^3<br>*0.750 x 10^9 – 0.999 x 10^9/L*	< 750/mm^3<br>*< 0.750 x 10^9/L*
**Infant□†, 1 day**	4,000 – 5,000/mm^3<br>*4.000 x 10^9 – 5.000 x 10^9/L*	3,000 – 3,999/mm^3<br>*3.000 x 10^9 – 3.999 x10^9/L*	1,500 – 2,999/mm^3<br>*1.500 x 10^9 – 2.999 x 10^9/L*	< 1,500/mm^3<br>*< 1.500 x 10^9/L*
Fibrinogen, decreased	100 – 200 mg/dL<br>*1.00 – 2.00 g/L*<br>OR<br>0.75 – 0.99 x LLN	75 – 99 mg/dL<br>*0.75 – 0.99 g/L*<br>OR<br>0.50 – 0.74 x LLN	50 – 74 mg/dL<br>*0.50 – 0.74 g/L*<br>OR<br>0.25 – 0.49 x LLN	< 50 mg/dL<br>*< 0.50 g/L*<br>OR<br>< 0.25 x LLN<br>OR<br>Associated with gross bleeding
Hemoglobin (Hgb)				
**Adult and Pediatric ≥ 57 days** (HIV POSITIVE ONLY)	8.5 – 10.0 g/dL<br>*1.32 – 1.55 mmol/L*	7.5 – 8.4 g/dL<br>*1.16 – 1.31 mmol/L*	6.50 – 7.4 g/dL<br>*1.01 – 1.15 mmol/L*	< 6.5 g/dL<br>*< 1.01 mmol/L*
**Adult and Pediatric ≥ 57 days** (HIV NEGATIVE ONLY)	10.0 – 10.9 g/dL<br>*1.55 – 1.69 mmol/L*<br>OR<br>Any decrease<br>2.5 – 3.4 g/dL<br>*0.39 – 0.53 mmol/L*	9.0 – 9.9 g/dL<br>*1.40 – 1.54 mmol/L*<br>OR<br>Any decrease<br>3.5 – 4.4 g/dL<br>*0.54 – 0.68 mmol/L*	7.0 – 8.9 g/dL<br>*1.09 – 1.39 mmol/L*<br>OR<br>Any decrease<br>≥ 4.5 g/dL<br>*≥ 0.69 mmol/L*	< 7.0 g/dL<br>*< 1.09 mmol/L*
**Infant□†, 36 – 56 days** (HIV POSITIVE OR NEGATIVE)	8.5 – 9.4 g/dL<br>*1.32 – 1.46 mmol/L*	7.0 – 8.4 g/dL<br>*1.09 – 1.31 mmol/L*	6.0 – 6.9 g/dL<br>*0.93 – 1.08 mmol/L*	< 6.00 g/dL<br>*< 0.93 mmol/L*
**Infant□†, 22 – 35 days** (HIV POSITIVE OR NEGATIVE)	9.5 – 10.5 g/dL<br>*1.47 – 1.63 mmol/L*	8.0 – 9.4 g/dL<br>*1.24 – 1.46 mmol/L*	7.0 – 7.9 g/dL<br>*1.09 – 1.23 mmol/L*	< 7.00 g/dL<br>*< 1.09 mmol/L*
**Infant□†, 1 – 21 days** (HIV POSITIVE OR NEGATIVE)	12.0 – 13.0 g/dL<br>*1.86 – 2.02 mmol/L*	10.0 – 11.9 g/dL<br>*1.55 – 1.85 mmol/L*	9.0 – 9.9 g/dL<br>*1.40 – 1.54 mmol/L*	< 9.0 g/dL<br>*< 1.40 mmol/L*
International Normalized Ratio of prothrombin time (INR)	1.1 – 1.5 x ULN	1.6 – 2.0 x ULN	2.1 – 3.0 x ULN	> 3.0 x ULN
Methemoglobin	5.0 – 10.0%	10.1 – 15.0%	15.1 – 20.0%	> 20.0%
Prothrombin Time (PT)	1.1 – 1.25 x ULN	1.26 – 1.50 x ULN	1.51 – 3.00 x ULN	> 3.00 x ULN
Partial Thromboplastin Time (PTT)	1.1 – 1.66 x ULN	1.67 – 2.33 x ULN	2.34 – 3.00 x ULN	> 3.00 x ULN
Platelets, decreased	100,000 – 124,999/mm^3<br>*100.000 x 10^9 – 124.999 x 10^9/L*	50,000 – 99,999/mm^3<br>*50.000 x 10^9 – 99.999 x 10^9/L*	25,000 – 49,999/mm^3<br>*25.000 x 10^9 – 49.999 x 10^9/L*	< 25,000/mm^3<br>*< 25.000 x 10^9/L*
WBC, decreased	2,000 – 2,500/mm^3<br>*2.000 x 10^9 – 2.500 x 10^9/L*	1,500 – 1,999/mm^3<br>*1.500 x 10^9 – 1.999 x 10^9/L*	1,000 – 1,499/mm^3<br>*1.000 x 10^9 – 1.499 x 10^9/L*	< 1,000/mm^3<br>*< 1.000 x 10^9/L*
**CHEMISTRIES**	***Standard International Units are listed in italics***			
Acidosis	NA	pH < normal, but ≥ 7.3	pH < 7.3 without life-threatening consequences	pH < 7.3 with life-threatening consequences
Albumin, serum, low	3.0 g/dL – < LLN<br>*30 g/L – < LLN*	2.0 – 2.9 g/dL<br>*20 – 29 g/L*	< 2.0 g/dL<br>*< 20 g/L*	NA
Alkaline Phosphatase	1.25 – 2.5 x ULN†	2.6 – 5.0 x ULN†	5.1 – 10.0 x ULN†	> 10.0 x ULN†
Alkalosis	NA	pH > normal, but ≤ 7.5	pH > 7.5 without life-threatening consequences	pH > 7.5 with life-threatening consequences
ALT (SGPT)	1.25 – 2.5 x ULN	2.6 – 5.0 x ULN	5.1 – 10.0 x ULN	> 10.0 x ULN
AST (SGOT)	1.25 – 2.5 x ULN	2.6 – 5.0 x ULN	5.1 – 10.0 x ULN	> 10.0 x ULN
Bicarbonate, serum, low	16.0 mEq/L – < LLN<br>*16.0 mmol/L – < LLN*	11.0 – 15.9 mEq/L<br>*11.0 – 15.9 mmol/L*	8.0 – 10.9 mEq/L<br>*8.0 – 10.9 mmol/L*	< 8.0 mEq/L<br>*< 8.0 mmol/L*
Bilirubin (Total)				
**Adult and Pediatric > 14 days**	1.1 – 1.5 x ULN	1.6 – 2.5 x ULN	2.6 – 5.0 x ULN	> 5.0 x ULN
**Infant□†, ≤ 14 days** (non-hemolytic)	NA	20.0 – 25.0 mg/dL<br>*342 – 428 µmol/L*	25.1 – 30.0 mg/dL<br>*429 – 513 µmol/L*	> 30.0 mg/dL<br>*> 513.0 µmol/L*

LABORATORY				
**PARAMETER**	**GRADE 1<br>MILD**	**GRADE 2<br>MODERATE**	**GRADE 3<br>SEVERE**	**GRADE 4<br>POTENTIALLY<br>LIFE-THREATENING**
**Infant□†, ≤ 14 days**<br>(hemolytic)	NA	NA	20.0 – 25.0 mg/dL<br>*342 – 428 µmol/L*	> 25.0 mg/dL<br>*> 428 µmol/L*
Calcium, serum, high (corrected for albumin)				
**Adult and Pediatric ≥ 7 days**	10.6 – 11.5 mg/dL<br>*2.65 – 2.88 mmol/L*	11.6 – 12.5 mg/dL<br>*2.89 – 3.13 mmol/L*	12.6 – 13.5 mg/dL<br>*3.14 – 3.38 mmol/L*	> 13.5 mg/dL<br>*> 3.38 mmol/L*
**Infant□†, < 7 days**	11.5 – 12.4 mg/dL<br>*2.88 – 3.10 mmol/L*	12.5 – 12.9 mg/dL<br>*3.11 – 3.23 mmol/L*	13.0 – 13.5 mg/dL<br>*3.245 – 3.38 mmol/L*	> 13.5 mg/dL<br>*> 3.38 mmol/L*
Calcium, serum, low (corrected for albumin)				
**Adult and Pediatric ≥ 7 days**	7.8 – 8.4 mg/dL<br>*1.95 – 2.10 mmol/L*	7.0 – 7.7 mg/dL<br>*1.75 – 1.94 mmol/L*	6.1 – 6.9 mg/dL<br>*1.53 – 1.74 mmol/L*	< 6.1 mg/dL<br>*< 1.53 mmol/L*
**Infant□†, < 7 days**	6.5 – 7.5 mg/dL<br>*1.63 – 1.88 mmol/L*	6.0 – 6.4 mg/dL<br>*1.50 – 1.62 mmol/L*	5.50 – 5.90 mg/dL<br>*1.38 – 1.51 mmol/L*	< 5.50 mg/dL<br>*< 1.38 mmol/L*
Cardiac troponin I (cTnI)	NA	NA	NA	Levels consistent with myocardial infarction or unstable angina as defined by the manufacturer
Cardiac troponin T (cTnT)	NA	NA	NA	≥ 0.20 ng/mL<br>OR<br>Levels consistent with myocardial infarction or unstable angina as defined by the manufacturer
Cholesterol (fasting)				
**Adult ≥ 18 years**	200 – 239 mg/dL<br>*5.18 – 6.19 mmol/L*	240 – 300 mg/dL<br>*6.20 – 7.77 mmol/L*	> 300 mg/dL<br>*> 7.77 mmol/L*	NA
**Pediatric < 18 years**	170 – 199 mg/dL<br>*4.40 – 5.15 mmol/L*	200 – 300 mg/dL<br>*5.16 – 7.77 mmol/L*	> 300 mg/dL<br>*> 7.77 mmol/L*	NA
Creatine Kinase	3.0 – 5.9 x ULN†	6.0 – 9.9 x ULN†	10.0 – 19.9 x ULN†	≥20.0 x ULN†
Creatinine	1.1 – 1.3 x ULN†	1.4 – 1.8 x ULN†	1.9 – 3.4 x ULN†	≥3.5 x ULN†
Glucose, serum, high				
Nonfasting	116 – 160 mg/dL<br>*6.44 – 8.88 mmol/L*	161 – 250 mg/dL<br>*8.89 – 13.88 mmol/L*	251 – 500 mg/dL<br>*13.89 – 27.75 mmol/L*	> 500 mg/dL<br>*> 27.75 mmol/L*
Fasting	110 – 125 mg/dL<br>*6.11 – 6.94 mmol/L*	126 – 250 mg/dL<br>*6.95 – 13.88 mmol/L*	251 – 500 mg/dL<br>*13.89 – 27.75 mmol/L*	> 500 mg/dL<br>*> 27.75 mmol/L*
Glucose, serum, low				
**Adult and Pediatric ≥ 1 month**	55 – 64 mg/dL<br>*3.05 – 3.55 mmol/L*	40 – 54 mg/dL<br>*2.22 – 3.06 mmol/L*	30 – 39 mg/dL<br>*1.67 – 2.23 mmol/L*	< 30 mg/dL<br>*< 1.67 mmol/L*
**Infant□†, < 1 month**	50 – 54 mg/dL<br>*2.78 – 3.00 mmol/L*	40 – 49 mg/dL<br>*2.22 – 2.77 mmol/L*	30 – 39 mg/dL<br>*1.67 – 2.21 mmol/L*	< 30 mg/dL<br>*< 1.67 mmol/L*
Lactate	< 2.0 x ULN without acidosis	≥ 2.0 x ULN without acidosis	Increased lactate with pH < 7.3 without life-threatening consequences	Increased lactate with pH < 7.3 with life-threatening consequences
LDL cholesterol (fasting)				
**Adult ≥ 18 years**	130 – 159 mg/dL<br>*3.37 – 4.12 mmol/L*	160 – 190 mg/dL<br>*4.13 – 4.90 mmol/L*	≥ 190 mg/dL<br>*≥ 4.91 mmol/L*	NA
**Pediatric > 2 - < 18 years**	110 – 129 mg/dL<br>*2.85 – 3.34 mmol/L*	130 – 189 mg/dL<br>*3.35 – 4.90 mmol/L*	≥ 190 mg/dL<br>*≥ 4.91 mmol/L*	NA
Lipase	1.1 – 1.5 x ULN	1.6 – 3.0 x ULN	3.1 – 5.0 x ULN	> 5.0 x ULN
Magnesium, serum, low	1.2 – 1.4 mEq/L<br>*0.60 – 0.70 mmol/L*	0.9 – 1.1 mEq/L<br>*0.45 – 0.59 mmol/L*	0.6 – 0.8 mEq/L<br>*0.30 – 0.44 mmol/L*	< 0.60 mEq/L<br>*< 0.30 mmol/L*
Pancreatic amylase	1.1 – 1.5 x ULN	1.6 – 2.0 x ULN	2.1 – 5.0 x ULN	> 5.0 x ULN
Phosphate, serum, low				
**Adult and Pediatric > 14 years**	2.5 mg/dL – < LLN<br>*0.81 mmol/L – < LLN*	2.0 – 2.4 mg/dL<br>*0.65 – 0.80 mmol/L*	1.0 – 1.9 mg/dL<br>*0.32 – 0.64 mmol/L*	< 1.00 mg/dL<br>*< 0.32 mmol/L*
**Pediatric 1 year – 14 years**	3.0 – 3.5 mg/dL<br>*0.97 – 1.13 mmol/L*	2.5 – 2.9 mg/dL<br>*0.81 – 0.96 mmol/L*	1.5 – 2.4 mg/dL<br>*0.48 – 0.80 mmol/L*	< 1.50 mg/dL<br>*< 0.48 mmol/L*
**Pediatric < 1 year**	3.5 – 4.5 mg/dL<br>*1.13 – 1.45 mmol/L*	2.5 – 3.4 mg/dL<br>*0.81 – 1.12 mmol/L*	1.5 – 2.4 mg/dL<br>*0.48 – 0.80 mmol/L*	< 1.50 mg/dL<br>*< 0.48 mmol/L*
Potassium, serum, high	5.6 – 6.0 mEq/L<br>*5.6 – 6.0 mmol/L*	6.1 – 6.5 mEq/L<br>*6.1 – 6.5 mmol/L*	6.6 – 7.0 mEq/L<br>*6.6 – 7.0 mmol/L*	> 7.0 mEq/L<br>*> 7.0 mmol/L*
Potassium, serum, low	3.0 – 3.4 mEq/L	2.5 – 2.9 mEq/L	2.0 – 2.4 mEq/L	< 2.0 mEq/L

LABORATORY				
**PARAMETER**	**GRADE 1**<br>**MILD**	**GRADE 2**<br>**MODERATE**	**GRADE 3**<br>**SEVERE**	**GRADE 4**<br>**POTENTIALLY LIFE-THREATENING**
	3.0 – 3.4 mmol/L	*2.5 – 2.9 mmol/L*	*2.0 – 2.4 mmol/L*	*< 2.0 mmol/L*
Sodium, serum, high	146 – 150 mEq/L<br>*146 – 150 mmol/L*	151 – 154 mEq/L<br>*151 – 154 mmol/L*	155 – 159 mEq/L<br>*155 – 159 mmol/L*	≥ 160 mEq/L<br>*≥ 160 mmol/L*
Sodium, serum, low	130 – 135 mEq/L<br>*130 – 135 mmol/L*	125 – 129 mEq/L<br>*125 – 129 mmol/L*	121 – 124 mEq/L<br>*121 – 124 mmol/L*	≤ 120 mEq/L<br>*≤ 120 mmol/L*
Triglycerides (fasting)	NA	500 – 750 mg/dL<br>*5.65 – 8.48 mmol/L*	751 – 1,200 mg/dL<br>*8.49 – 13.56 mmol/L*	> 1,200 mg/dL<br>*> 13.56 mmol/L*
Uric acid	7.5 – 10.0 mg/dL<br>*0.45 – 0.59 mmol/L*	10.1 – 12.0 mg/dL<br>*0.60 – 0.71 mmol/L*	12.1 – 15.0 mg/dL<br>*0.72 – 0.89 mmol/L*	> 15.0 mg/dL<br>*> 0.89 mmol/L*
**URINALYSIS**	***Standard International Units are listed in italics***			
Hematuria (microscopic)	6 – 10 RBC/HPF	> 10 RBC/HPF	Gross, with or without clots OR with RBC casts	Transfusion indicated
Proteinuria, random collection	1 +	2 – 3 +	4 +	NA
Proteinuria, 24 hour collection				
**Adult and Pediatric ≥ 10 years**	200 – 999 mg/24 h<br>*0.200 – 0.999 g/d*	1,000 – 1,999 mg/24 h<br>*1.000 – 1.999 g/d*	2,000 – 3,500 mg/24 h<br>*2.000 – 3.500 g/d*	> 3,500 mg/24 h<br>*> 3.500 g/d*
**Pediatric > 3 mo - < 10 years**	201 – 499 mg/m2/24 h<br>*0.201 – 0.499 g/d*	500 – 799 mg/m2/24 h<br>*0.500 – 0.799 g/d*	800 – 1,000 mg/m2/24 h<br>*0.800 – 1.000 g/d*	> 1,000 mg/ m2/24 h<br>*> 1.000 g/d*

☐ Values are for term infants.
† Use age and sex appropriate values (e.g., bilirubin), including preterm infants.

APPENDIX 8: DIAGNOSTIC CRITERIA FOR SERIOUS AIDS-DEFINING ILLNESS

These criteria are based on the 1993 US Centers for Disease Control and Prevention criteria for category C disease (ref MMWR 1992; 41 [No RR-17]: 1-19), but excluding oesophageal candidiasis and chronic mucocutaneous herpes simplex virus infection. Events that meet either the presumptive criteria (if available) or definitive criteria will count as endpoints in this trial.

	PRESUMPTIVE CRITERIA	DEFINITIVE CRITERIA
**CONSTITUTIONAL DISEASE**		
HIV Wasting syndrome	Unexplained, involuntary weight loss >10% from baseline (week 0) PLUS persistent diarrhoea with 2 or more liquid stools/day > 1 month OR chronic weakness OR persistent fever > 1 month. Should exclude other causes such as cancer, TB, MAI, cryptosporidiosis or other specific enteritis.	none
**INFECTIONS**		
Candidiasis of bronchi, trachea or lungs	none	macroscopic appearance at bronchoscopy or autopsy, or histology or cytology/smear (not culture)
Coccidiodomycosis, disseminated or extrapulmonary	none	histology or cytology, culture or antigen detection from affected tissue
Cryptococcosis, meningitis or extrapulmonary	none	histology or cytology/microscopy, culture or antigen detection from affected tissue
Cryptosporidiosis	none	persistent diarrhoea > 1 month, histology or microscopy
CMV retinitis	Symptomatic or asymptomatic. Typical appearance on fundoscopy of discrete patches of retinal whitening, spreading along blood vessels, associated with vasculitis, haemorrhage and necrosis, confirmed by ophthalmologist.	none
CMV end-organ disease	none	compatible symptoms, plus histology or detection of antigen from affected tissue
CMV radiculomyelitis	Leg weakness and decreased reflexes or syndrome consistent with cord lesion presenting subacutely over days to weeks. Myelogram shows no mass lesion. CSF shows >5 WBC with >50% polymorphs and no other pathogen or persistence of symptoms after appropriate treatment for other pathogens, OR CMV shown by PCR, antigen or culture.	none
CMV meningoencephalitis	Rapid (days to 1-4 weeks) syndrome with progressive delirium, cognitive impairment +/- seizures and fever (often with other CMV disease elsewhere). CT/MRI may show periventricular abnormalities with or without contrast enhancement. CSF may be normal or show evidence of CMV.	none
HSV visceral disease, e.g. bronchitis, pneumonitis, oesophagitis	none	symptoms, plus histology or culture or detection of antigen from affected tissue
Histoplasmosis, disseminated or extrapulmonary	none	symptoms, plus histology or culture or detection of antigen from affected tissues
Isosporiasis	none	persistent diarrhoea > 1 month, histology or microscopy
Leishmaniasis, visceral	none	symptoms, plus histology
Microsporidiosis	none	persistent diarrhoea > 1 month, histology or microscopy
MAC, and other atypical mycobacteriosis	Symptoms of fever, fatigue, anaemia or diarrhoea, plus AFBs seen in stool, blood, body fluid or tissue but not grown on culture, and no concurrent diagnosis of TB, except pulmonary	symptoms of fever, fatigue, anaemia or diarrhoea, culture from stool, blood, body fluid or tissue, except pulmonary
Tuberculosis, pulmonary	Symptoms of fever, dyspnoea, cough, weight loss or fatigue, plus AFBs seen in sputum or lavage or lung tissue but not grown in culture, plus responds to standard TB treatment	symptoms of fever, dyspnoea, cough, weight loss or fatigue, plus culture from sputum or lavage or lung tissue

	PRESUMPTIVE CRITERIA	DEFINITIVE CRITERIA
Tuberculosis, extrapulmonary	Symptoms, plus AFBs seen from affected tissue or blood but not grown in culture, concurrent diagnosis of pulmonary TB or responds to standard TB treatment	symptoms, plus culture from blood or affected tissue
PCP	Recent symptoms, plus typical CXR appearance if on PCP prophylaxis or any CXR appearance if not on prophylaxis and CD4+ <200, negative bronchoscopy if already treated for PCP for > 7 days or not done, no bacterial pathogens in sputum, and responds to PCP treatment	microscopy or histology
Extrapulmonary *pneumocystis*	none	symptoms plus microscopy or histology
Recurrent bacterial pneumonia	Second pneumonic episode within 1 year, new CXR appearance, symptoms and signs, diagnosed by a doctor	second pneumonic episode with 1 year, new CXR appearance, detection of bacterial pathogen
PML, Progressive multifocal leukoencephalopathy	Symptoms and brain scan consistent with PML, and no response to toxo treatment	histology
Recurrent salmonella septicaemia	none	second distinct episode, culture
Cerebral toxoplasmosis	Symptoms of focal intracranial abnormality or decreased consciousness, and brain scan consistent with lesion(s) having mass effect or enhanced by contrast, and either positive toxoplasma serology or responds to treatment clinically and by scan	histology or microscopy
Other extrapulmonary toxoplasmosis	none	symptoms plus histology or microscopy
**NEOPLASMS**		
KS, Kaposi's sarcoma	Typical appearance without resolution. clinicians who have seen few cases should not make presumptive diagnoses	Histology
Primary cerebral lymphoma	Symptoms consistent with lymphoma, at least one lesion with mass effect on brain scan, no response clinically and by scan to toxoplasma treatment	
B-cell, non-Hodgkin's lymphoma	none	histology
Cervical carcinoma, invasive	none	histology, not carcinoma-in-situ
**NEUROLOGICAL**		
HIV encephalopathy	Cognitive or motor dysfunction interfering with usual activity, progressive over weeks or months in the absence of another condition to explain the findings, should have brain scan +/- CSF to exclude other causes. should be grade 2 or worse in at least 2 domains by NARS (see below) excluding abnormal domains at trial entry	none
**OTHER**		
Indeterminate intracerebral lesion(s)	Neurological illness with evidence for an intracerebral lesion(s) by brain scan where the differential diagnosis is either cerebral toxoplasmosis, PML, cerebral lymphoma or HIV encephalopathy	none

ABBREVIATED NARS (Neuropsychiatric AIDS Rating Scale) grading for HIV ENCEPHALOPATHY

Adapted from: Price RW, Brew BJ. The AIDS dementia complex. J Infect Dis 1988; 158 (5): 1079-83, and Hughes CP, Berg L, Danziger WL. A new clinical scale for the staging of dementia. Brit J Psych 1982; 140: 566-92.

	Cognitive-Behavioural Domains					
	**Orientation**	**Memory**	**Motor**	**Behaviour**	**Problem solving**	**Activities of daily living**
**NARS stage**						
0.5	fully oriented	complains of memory problems	fully ambulatory slightly slowed movements	normal	has slight mental slowing	slight impairment in business dealings
1	fully oriented, may have brief periods of "spaciness	mild memory problems	balance, co-ordination and handwriting difficulties	more irritable, labile or apathetic, withdrawn	difficulty planning and completing work	can do simple daily tasks, may need prompting
2	some disorientation	memory moderately impaired, new learning impaired	ambulatory but may require walking aid	some impulsivity or agitated behaviour	severe impairment, poor social judgement, gets lost easily	needs assistance with ADLs
3	frequent disorientation	severe memory loss, only fragments of memory remain	ambulatory with assistance	may have organic psychosis	judgement very poor	cannot live independently
4	confused and disoriented	virtually no memory	bedridden	mute and unresponsive	no problem solving ability	nearly vegetative

APPENDIX 9: DIAGNOSTIC CRITERIA FOR SERIOUS NON-AIDS-DEFINING ILLNESS

These criteria are based on those used in long term clinical endpoint trials by the INSIGHT research network, with additional criteria for acute liver failure, severe acute pancreatitis, severe lactic acidaemia, severe facial lipoatrophy and severe peripheral neuropathy that are based on standard toxicity criteria or definitions developed in case-definition studies.

Serious Non-AIDS event	DIAGNOSTIC CRITERIA
Acute myocardial infarction (MI)	A or (B+C) or (B+D): (A) Acute MI demonstrated as the cause of death on autopsy; (B) Occurrence of a compatible clinical syndrome, including symptoms (e.g. chest pain) consistent with myocardial ischaemia; (C) Development of (i) evolving new Q waves, or (ii) evolving ST elevation, based on at least two EKGs taken during the same hospital admission; (D) Diagnostic elevation of CK-MB to more than twice the upper limit of normal in the laboratory performing the study, or diagnostic elevation of troponin above ULN.
Coronary artery disease requiring invasive procedures	Written report in medical record detailing procedure performed for treating coronary artery disease, including: coronary artery bypass graft, coronary artery stent implant, coronary artherectomy, and percutaneous transluminal angioplasty.
Cirrhosis	(A+B+C) or (A+B+D) or (A+B+E) or F or G: (A) Clinical evidence of cirrhosis, with at least one of the following: ascites, hepatic encephalopathy, gastric or oesophageal varices, or signs of portal hypertension on endoscopy, without another explanation for these symptoms; (B) At least one of the following: Increased PT or INR above ULN, serum AST > serum ALT, platelet count <150,000; (C) albumin <3 g/dL or <30 g/L; (D) A positive result on an approved diagnostic fibrosis panel, e.g. Fibrosure/Fibrotest; (E) A positive result on transient elastography (Fibroscan) consistent with cirrhosis; (F) MRI, CT or ultrasound imaging consistent with cirrhosis (e.g. nodular liver, reversal of flow in portal vein); (G) Histologic evidence obtained by liver biopsy or autopsy.
Acute liver failure	ALT or AST greater than 5 times ULN, with clinical jaundice and encephalopathy.
End-stage renal disease	A or B or C: (A) Haemodialysis or pertitoneal dialysis for a period of at least three months, documented in a clinical note; (B) A kidney transplant, documented in a clinical note; (C) two consecutive measurements of serum creatinine clearance rate < 15 ml/min per 1.73 m^2 calculated using the Cockcroft-Gault equation.
Stroke	(A+D) or (A+B) or (A+C) or D or E: (A) Acute onset with a clinically compatible course, including unequivocal objective findings of a localising neurologic deficit; (B) CT or MRI compatible with diagnosis of stroke and current neurologic signs and symptoms; (C) Positive lumbar puncture compatible with subarachnoid haemorrhage; (D) Stroke diagnosed as cause of death at autopsy; (E) Death certificate or death note from medical record listing stroke as cause of death.
Severe acute pancreatitis	Severe abdominal pain requiring hospitalisation and blood amylase levels greater than 2.5 times the upper limit of normal.
Severe lactic acidaemia	Two consecutive measures of peripheral blood lactate > 5 mmol/l (45 mg/dl) or demonstrated lactic acidosis (arterial blood pH < 7.34, blood bicarbonate < 20 mmol/l and blood lactate levels above normal range).
Severe facial lipoatrophy	Facial fat loss that is considered to be obvious to both patient and clinician and that is considered by the clinician to have the characteristic appearance of HIV-associated lipoatrophy.
Severe peripheral neuropathy	Severe pain, numbness or tingling in the feet and/or legs that interferes with normal activities and requires narcotic analgesia for control.
Non-AIDS malignancy (excluding Kaposi's sarcoma (KS), lymphoma, invasive cervical cancer)	Diagnosis of cancer other than lymphoma, KS or invasive cervical cancer and a written report in the medical record from the hospitalisation during which the diagnosis was established, or in a pathology report that established the diagnosis, or in an autopsy report.

APPENDIX 10: PROTEASE INHIBITOR INFORMATION

BNF November 2007 (www.bnf.org)

Cautions
Protease inhibitors are associated with hyperglycaemia and should be used with caution in diabetes (see Lipodystrophy Syndrome). Caution is also needed in patients with haemophilia who may be at increased risk of bleeding. Protease inhibitors should be used with caution in hepatic impairment (BNF Appendix 2); the risk of hepatic side-effects is increased in patients with chronic hepatitis B or C. Atazanavir, darunavir, fosamprenavir, and tipranavir may be used at usual doses in patients with renal impairment, but other protease inhibitors should be used with caution in renal impairment (BNF Appendix 3). Protease inhibitors should also be used with caution during pregnancy (BNF Appendix 4).

Side-effects
Side-effects of the protease inhibitors include gastro-intestinal disturbances (including diarrhoea, nausea, vomiting, abdominal pain, flatulence), anorexia, hepatic dysfunction, pancreatitis; blood disorders including anaemia, neutropenia, and thrombocytopenia; sleep disturbances, fatigue, headache, dizziness, paraesthesia, myalgia, myositis, rhabdomyolysis; taste disturbances; rash, pruritus, Stevens-Johnson syndrome, hypersensitivity reactions including anaphylaxis; see also notes above for lipodystrophy and metabolic effects (Lipodystrophy Syndrome), and osteonecrosis.

Lipodystrophy Syndrome
Metabolic effects associated with antiretroviral treatment include fat redistribution, insulin resistance and dyslipidaemia; collectively these have been termed lipodystrophy syndrome.

Fat redistribution (with loss of subcutaneous fat, increased abdominal fat, 'buffalo hump' and breast enlargement) is associated with regimens containing protease inhibitors and nucleoside reverse transcriptase inhibitors. Stavudine, and to a lesser extent zidovudine, are associated with a higher risk of lipoatrophy and should be used only if alternative regimens are not suitable.

Dyslipidaemia (with adverse effects on body lipids) is associated with antiretroviral treatment, particularly with protease inhibitors. Protease inhibitors are associated with insulin resistance and hyperglycaemia. Plasma lipids, blood glucose and the usual risk factors for atherosclerotic disease should be taken into account before prescribing regimens containing a protease inhibitor; patients receiving protease inhibitors should be monitored for changes in plasma lipids and blood glucose.

Sub-sections:
AMPRENAVIR
ATAZANAVIR
DARUNAVIR
FOSAMPRENAVIR
INDINAVIR
LOPINAVIR WITH RITONAVIR
NELFINAVIR
RITONAVIR
SAQUINAVIR
TIPRANAVIR

**AMPRENAVIR**		
**Interactions**	Liver disease	Avoid oral solution due to high propylene glycol content; without low-dose ritonavir, reduce dose of amprenavir capsules to 450 mg every 12 hours in moderate hepatic impairment and reduce dose to 300 mg every 12 hours in severe impairment
	Renal impairment	Mild-moderate: Use oral solution with caution due to high propylene glycol content<br>Severe: Avoid oral solution
	Pregnancy	Avoid oral solution due to high propylene glycol content; manufacturer advises use capsules only if potential benefit outweighs risk
**Indications**	HIV infection in combination with other antiretroviral drugs in patients previously treated with other protease inhibitors	
**Cautions**	Rash. Rash may occur, usually in the second week of therapy; discontinue permanently if severe rash with systemic or allergic symptoms or, mucosal involvement; if rash mild or moderate, may continue without interruption—rash usually resolves within 2 weeks and may respond to antihistamines	
**Contra-indications**	Breast-feeding not advised in HIV infection	
**Side-effects**	see notes above; also reported, rash including rarely Stevens-Johnson syndrome (see also above); tremors, oral or perioral paraesthesia, mood disorders including depression	
**Dose**	Agenerase®(GSK)<br>Capsules, ivory, amprenavir 50 mg.<br>Excipients include vitamin E 36 units/50 mg amprenavir (avoid vitamin E supplements)<br>Dose adult and adolescent over 12 years, body-weight over 50 kg, 1.2 g every 12 hours; adult and adolescent over 12 years, body-weight under 50 kg and child 4–12 years, 20 mg/kg every 12 hours (max. 2.4 g daily)<br>With low-dose ritonavir, adult and adolescent over 12 years, body-weight over 50 kg, amprenavir 600 mg every 12 hours with ritonavir 100 mg every 12 hours<br>Oral solution , grape-bubblegum- and peppermint-flavoured, amprenavir 15 mg/mL.<br>Excipients include vitamin E 46 units/mL (avoid vitamin E supplements), propylene glycol 550 mg/mL (see Excipients)<br>Electrolytes: K+ 26 micromol/mL, Na+ 174 micromol/mL<br>Dose adult and child over 4 years, 17 mg/kg every 8 hours (max. 2.8 g daily); child under 4 years not recommended<br>Note: The bioavailability of Agenerase® oral solution is lower than that of capsules; the two formulations are not interchangeable on a milligram-for-milligram basis	
**ATAZANAVIR**		
**Interactions**	Liver disease	Manufacturer advises caution in mild hepatic impairment; avoid in moderate to severe hepatic impairment
	Pregnancy	Manufacturer advises use only if potential benefit outweighs risk; theoretical risk of hyperbilirubinaemia in neonate if used at term
**Indications**	HIV infection in combination with other antiretroviral drugs in patients previously treated with antiretrovirals	
**Cautions**	see notes above; also concomitant use with drugs that prolong PR interval; cardiac conduction disorders; predisposition to QT interval prolongation (including electrolyte disturbances, concomitant use of drugs that prolong QT interval); interactions: BNF Appendix 1 (atazanavir)	
**Contra-indications**	Breast-feeding not advised in HIV infection	
**Side-effects**	see notes above; also peripheral neurological symptoms; less commonly mouth ulcers, hypertension, syncope, chest pain, dyspnoea, abnormal dreams, amnesia, depression, anxiety, weight changes, increased appetite, gynaecomastia, nephrolithiasis, urinary frequency, haematuria, proteinuria, arthralgia, and alopecia; rarely hepatosplenomegaly, oedema, palpitation, and abnormal gait	
**Dose**	with low-dose ritonavir and food, adult over 18 years, 300 mg once daily with ritonavir 100 mg once daily	
	Reyataz®(Bristol-Myers Squibb)<br>Capsules , atazanavir (as sulphate) 100 mg (dark blue/white); 150 mg (dark blue/light blue); 200 mg (dark blue),	
**DARUNAVIR**		
**Interactions**	Liver disease	Manufacturer advises caution in mild to moderate hepatic impairment; avoid in severe hepatic impairment—no information available

	Pregnancy	Manufacturer advises use only if potential benefit outweighs risk
**Indications**	HIV infection (that has not responded to treatment with other protease inhibitors) in combination with other antiretroviral drugs	
**Cautions**	see notes above; also sulphonamide sensitivity	
**Contra-indications**	Breast-feeding not advised in HIV infection	
**Side-effects**	see notes above; also myocardial infarction, transient ischaemic attack, syncope, tachycardia, hypertension, flushing, peripheral oedema, dyspnoea, cough, hiccups, peripheral neuropathy, anxiety, confusion, memory impairment, mood changes, abnormal coordination, weight gain, hyperthermia, hypothyroidism, osteoporosis, gynaecomastia, erectile dysfunction, dysuria, polyuria, nephrolithiasis, renal failure, hyponatraemia, arthralgia, keratoconjuntivitis sicca, salivation changes, mouth ulcers, increased sweating, and alopecia	
**Dose**	With low-dose ritonavir, adult over 18 years, 600 mg twice daily<br>Missed dose If a dose is more than 6 hours late, the missed dose should not be taken and the next dose should be taken at the normal time	
	Prezista®(Janssen-Cilag)<br>Tablets , orange, f/c, darunavir (as ethanolate) 300 mg	
**FOSAMPRENAVIR**		
**Interactions**	Note Fosamprenavir is a pro-drug of amprenavir	
**Indications**	HIV infection in combination with other antiretroviral drugs	
**Cautions**	see notes above and under Amprenavir	
**Contra-indications**	Breast-feeding not advised in HIV infection	
**Side-effects**	see notes above and under Amprenavir	
**Dose**	with low-dose ritonavir, adult over 18 years, 700 mg twice daily<br>Note 700 mg fosamprenavir is equivalent to approx. 600 mg amprenavir	
	Telzir®(GSK)<br>Tablets , f/c, pink, fosamprenavir (as calcium) 700 mg<br>Oral suspension , fosamprenavir (as calcium) 50 mg/mL, (grape-bubblegum-and peppermint-flavoured) (with 10-mL oral syringe)	
**INDINAVIR**		
**Interactions**	Liver disease	Increased risk of nephrolithiasis; reduce dose to 600 mg every 8 hours in mild to moderate hepatic impairment; not studied in severe impairment
	Pregnancy	Toxicity in *animal* studies; manufacturer advises use only if potential benefit outweighs risk; theoretical risk of hyperbilirubinaemia and renal stones in neonate if used at term
**Indications**	HIV infection in combination with nucleoside reverse transcriptase inhibitors	
**Cautions**	see notes above; also ensure adequate hydration (risk of nephrolithiasis especially in children); patients at risk of nephrolithiasis (monitor for nephrolithiasis); avoid in porphyria (section 9.8.2); interactions: BNF Appendix 1 (indinavir)	
**Contra-indications**	Breast-feeding not advised in HIV infection	
**Side-effects**	see notes above; also reported, dry mouth, hypoaesthesia, dry skin, hyperpigmentation, alopecia, paronychia, interstitial nephritis (with medullary calcification and cortical atrophy in asymptomatic severe leucocyturia), nephrolithiasis (may require interruption or discontinuation; more frequent in children), dysuria, haematuria, crystalluria, proteinuria, pyuria (in children), pyelonephritis; haemolytic anaemia	
**Dose**	800 mg every 8 hours; child and adolescent4–17 years, 500 mg/m2 every 8 hours (max. 800 mg every 8 hours); child under 4 years, safety and efficacy not established	
	Crixivan®(MSD)<br>Capsules , indinavir (as sulphate), 200 mg; 400 mg, Counselling Administer 1 hour before or 2 hours after a meal; may be administered with a low-fat light meal; in combination with didanosine tablets, allow 1 hour between each drug (antacids in didanosine tablets reduce absorption of indinavir); in combination with low-dose ritonavir, give with food<br>Note Dispense in original container (contains dessicant)	
**LOPINAVIR WITH RITONAVIR**		
**Interactions**	Liver disease	Avoid oral solution because of propylene glycol content; manufacturer advises avoid capsules and tablets in severe hepatic impairment
	Renal impairment	Avoid oral solution due to propylene glycol content; use capsules and tablets with caution in severe impairment

	Pregnancy	Avoid oral solution due to high propylene glycol content; manufacturer advises use capsules and tablets only if potential benefit outweighs risk (toxicity in animal studies)
**Indications**	HIV infection in combination with other antiretroviral drugs	
**Cautions**	see notes above; concomitant use with drugs that prolong QT interval; pancreatitis (see below); interactions: BNF Appendix 1 (lopinavir, ritonavir)<br>Pancreatitis Signs and symptoms suggestive of pancreatitis (including raised serum lipase) should be evaluated—discontinue if pancreatitis diagnosed	
**Contra-indications**	Breast-feeding not advised in HIV infection	
**Side-effects**	see notes and Cautions above; also electrolyte disturbances in children; less commonly dysphagia, appetite changes, weight changes, cholecystitis, hypertension, myocardial infarction, palpitation, thrombophlebitis, vasculitis, chest pain, oedema, dyspnoea, cough, agitation, anxiety, amnesia, ataxia, hypertonia, confusion, depression, abnormal dreams, extrapyramidal effects, neuropathy, influenza-like syndrome, Cushing's syndrome, hypothyroidism, menorrhagia, amenorrhoea, sexual dysfunction, breast enlargement, dehydration, nephritis, hypercalciuria, lactic acidosis, arthralgia, hyperuricaemia, abnormal vision, otitis media, tinnitus, dry mouth, sialadenitis, mouth ulceration, periodontitis, acne, alopecia, dry skin, sweating, skin discoloration, nail disorders, rarely prolonged PR interval	
**Dose**	Kaletra®(Abbott)<br>Capsules , orange, lopinavir 133.3 mg, ritonavir 33.3 mg<br>Dose adult and child over 2 years with body surface area of 1.4 m2 or greater, 3 capsules twice daily with food; child over 2 years with body surface area less than 1.4 m2, oral solution preferred; if oral solution inappropriate and body surface area 0.4–0.75 m2, 1 capsule twice daily, body surface area 0.8–1.3 m2, 2 capsules twice daily<br>Tablets , yellow, f/c, lopinavir 200 mg, ritonavir 50 mg<br>Dose adult and child with body surface area greater than 1.3 m2 or body-weight 40 kg and over, 2 tablets twice daily<br>Oral solution , lopinavir 400 mg, ritonavir 100 mg/5 mL<br>Excipients include propylene glycol 153 mg/mL (see Excipients), alcohol 42%<br>Dose adult and adolescent, 5 mL twice daily with food; child over 2 years 2.9 mL/m2 twice daily with food, max. 5 mL twice daily; child under 2 years, safety and efficacy not established<br>Note 5 mL oral solution ≡ 3 capsules ≡ 2 tablets; where appropriate, capsules may be used instead of oral solution	
**NELFINAVIR**		
**Interactions**	Liver disease	No information available—manufacturer advises caution
	Renal impairment	No information available—manufacturer advises caution
	Pregnancy	No information available—manufacturer advises use only if potential benefit outweighs risk
**Indications**	HIV infection in combination with other antiretroviral drugs	
**Cautions**	see notes above; interactions: see BNF Appendix 1 (nelfinavir)	
**Contra-indications**	Breast-feeding not advised in HIV infection	
**Side-effects**	see notes above; also reported, fever	
**Dose**	1.25 g twice daily or 750 mg 3 times daily; child 3–13 years, initially 50–55 mg/kg twice daily (max. 1.25 g twice daily) or 25–30 mg/kg 3 times daily (max. 750 mg 3 times daily)	
	Viracept®(Roche)<br>Tablets , blue, f/c, nelfinavir (as mesilate) 250 mg<br>Oral powder , nelfinavir (as mesilate) 50 mg/g.<br>Excipients include aspartame (section 9.4.1)<br>Counselling Powder may be mixed with water, milk, formula feeds or pudding; it should not be mixed with acidic foods or juices owing to its taste	
**RITONAVIR**		
**Interactions**	Hepatic impairment	Avoid in decompensated liver disease; in severe hepatic impairment without decompensation, use 'booster' doses with caution (avoid treatment doses)
	Pregnancy	Manufacturer advises use only if potential benefit outweighs risk—no information available
**Indications**	HIV infection in combination with nucleoside reverse transcriptase inhibitors; low doses used to increase effect of some protease inhibitors	
**Cautions**	see notes above; avoid in porphyria (section 9.8.2); pancreatitis (see below); interactions: see BNF Appendix 1 (ritonavir).<br>Pancreatitis Signs and symptoms suggestive of pancreatitis (including raised serum lipase) should be evaluated—discontinue if pancreatitis diagnosed	

**Contra-indications**	Breast-feeding not advised in HIV infection	
**Side-effects**	see notes and Cautions above; also diarrhoea (may impair absorption—close monitoring required), vasodilatation, cough, throat irritation, anxiety, perioral and peripheral paraesthesia, hyperaesthesia, fever, decreased blood thyroxine concentration, electrolyte disturbances, raised uric acid, dry mouth, mouth ulcers, and sweating; less commonly increased prothrombin time and dehydration; syncope, postural hypotension, seizures, menorrhagia, and renal failure also reported	
**Dose**	initially 300 mg every 12 hours for 3 days, increased in steps of 100 mg every 12 hours over not longer than 14 days to 600 mg every 12 hours; child over 2 years initially 250 mg/m2 every 12 hours, increased by 50 mg/m2 at intervals of 2–3 days to 350 mg/m2 every 12 hours (max. 600 mg every 12 hours).<br>Low-dose booster to increase effect of other protease inhibitors, 100–200 mg once or twice daily	
	Norvir®(Abbott)<br>Capsules , ritonavir 100 mg<br>Excipients include alcohol 12%<br>Oral solution , sugar-free, ritonavir 400 mg/5 mL<br>Counselling Oral solution contains 43% alcohol; bitter taste can be masked by mixing with chocolate milk; do not mix with water, measuring cup must be dry	
	With lopinavir: See under Lopinavir with ritonavir	
**SAQUINAVIR**		
**Interactions**	Hepatic impairment	Manufacturer advises caution in moderate hepatic impairment; avoid in severe impairment
	Renal impairment	severe Dose adjustment possibly required
**Indications**	HIV infection in combination with other antiretroviral drugs	
**Cautions**	see notes above; concomitant use of garlic (avoid garlic capsules—reduces plasma-saquinavir concentration); interactions: BNF Appendix 1 (saquinavir)	
**Contra-indications**	Breast-feeding not advised in HIV infection	
**Side-effects**	see notes above; also dyspnoea, increased appetite, peripheral neuropathy, convulsions, changes in libido, renal impairment, dry mouth, and alopecia	
**Dose**	with low-dose ritonavir, adult and adolescent over 16 years, 1 g every 12 hours	
	Invirase®(Roche)<br>Capsules , brown/green, saquinavir (as mesilate) 200 mg<br>Tablets , orange, f/c, saquinavir (as mesilate) 500 mg	
**TIPRANAVIR**		
**Interactions**	See BNF Appendix 1	
**Indications**	HIV infection resistant to other protease inhibitors, in combination with other antiretroviral drugs in patients previously treated with antiretrovirals	
**Cautions**	see notes above; also patients at risk of increased bleeding from trauma, surgery or other pathological conditions; concomitant use of drugs that increase risk of bleeding; interactions: BNF Appendix 1 (tipranavir).<br>Hepatotoxicity Potentially life-threatening hepatotoxicity reported; monitor liver function before treatment then on weeks 2, 4 and 8 of treatment, then every 2–3 months (every 2 weeks for first 3 months then monthly in those with hepatic impairment (BNF Appendix 2)). Discontinue if signs or symptoms of hepatitis develop or if liver-function abnormality develops (consult product literature)	
**Contra-indications**	Breast-feeding not advised in HIV infection	
**Side-effects**	see notes above; also dyspnoea, anorexia, peripheral neuropathy, influenza-like symptoms, renal impairment and photosensitivity; rarely dehydration	
**Dose**	With low-dose ritonavir, 500 mg twice daily; child safety and efficacy not established	
	Aptivus®(Boehringer Ingelheim)<br>Capsules , pink, tipranavir 250 mg<br>Excipients include ethanol 100 mg per capsule	

APPENDIX 11: JOINT BRITISH SOCIETIES CARDIOVASCULAR RISK PREDICTION CHARTS

BNF September 2006 (www.bnf.org)

Cardiovascular Risk Prediction Charts

Heart 2005; **91**(Suppl V): v1–v52

How to use the Cardiovascular Risk Prediction Charts for Primary Prevention

These charts are for estimating cardiovascular disease (CVD) risk (non-fatal myocardial infarction and stroke, coronary and stroke death and new angina pectoris) for individuals who have **not** already developed coronary heart disease (CHD) or other major atherosclerotic disease. They are an aid to making clinical decisions about how intensively to intervene on lifestyle and whether to use antihypertensive, lipid lowering and anti-platelet medication, but should **not replace clinical judgment**.

- The use of these charts is **not appropriate** for patients who have existing diseases which already put them at high risk such as:
  - coronary heart disease or other major atherosclerotic disease;
  - familial hypercholesterolaemia or other inherited dyslipidaemias;
  - renal dysfunction including diabetic nephropathy;
  - type 1 and 2 diabetes mellitus.
- The charts should **not** be used to decide whether to introduce antihypertensive medication when blood pressure is persistently at or above 160/100 mmHg or when target organ damage due to hypertension is present. In both cases antihypertensive medication is recommended regardless of CVD risk. Similarly the charts should **not** be used to decide whether to introduce lipid-lowering medication when the ratio of serum total to HDL cholesterol exceeds 6. Such medication is generally then indicated regardless of estimated CVD risk.
- To estimate an individual's absolute 10-year risk of developing CVD choose the chart for his or her sex, lifetime smoking status and age. Within this square identify the level of risk according to the point where the coordinates for systolic blood pressure and the ratio of total cholesterol to high density lipoprotein (HDL) cholesterol meet. If no HDL cholesterol result is available, then assume this is 1.0 mmol/litre and the lipid scale can be used for total cholesterol alone.
- Higher risk individuals (red areas) are defined as those whose 10-year CVD risk exceeds 20%, which is approximately equivalent to the coronary heart disease risk of > 15% over the same period.
- The chart also assists in identifying individuals whose 10-year CVD risk is moderately increased in the range 10–20% (orange areas) and those in whom risk is lower than 10% over 10 years (green areas).
- Smoking status should reflect lifetime exposure to tobacco and not simply tobacco use at the time of assessment. For example, those who have given up smoking within 5 years should be regarded as current smokers for the purposes of the charts.
- The initial blood pressure and the first random (non-fasting) total cholesterol and HDL cholesterol can be used to estimate an individual's risk. However, the decision on using drug therapy should generally be based on repeat risk factor measurements over a period of time.

(Continued over)

- Men and women do not reach the level of risk predicted by the charts for the three age bands until they reach the ages 49, 59, and 69 years respectively. The charts will overestimate current risk most in the under 40s. Clinical judgement must be exercised in deciding on treatment in younger patients. However, it should be recognised that blood pressure and cholesterol tend to rise most and HDL cholesterol to decline most in younger people already with adverse levels. Left untreated, their risk at the age 49 years is likely to be higher than the projected risk shown on the age-under-50-years chart. From age 70 years the CVD risk, especially for men, is usually ≥ 20% over 10 years and the charts will underestimate true total CVD risk.

- These charts (and all other currently available methods of CVD risk prediction) are based on groups of people with **untreated** levels of blood pressure, total cholesterol and HDL cholesterol. In patients already receiving antihypertensive therapy in whom the decision is to be made about whether to introduce lipid-lowering medication, or vice versa, the charts can only act as a guide. Unless recent pre-treatment risk factor values are available it is generally safest to assume that CVD risk is higher than that predicted by current levels of blood pressure or lipids on treatment.

- CVD risk is also higher than indicated in the charts for:
  - those with a family history of premature CVD or stroke (male first-degree relatives aged < 55 years and female first-degree relatives aged < 65 years) which increases the risk by a factor of approximately 1.3;
  - those with raised triglyceride levels (> 1.7 mmol/litre);
  - women with premature menopause;
  - those who are not yet diabetic, but have impaired fasting glycaemia (6.1–6.9 mmol/litre) or impaired glucose tolerance (2 hour glucose ≥ 7.8 mmol/litre but < 11.1 mmol/litre in an oral glucose tolerance test).

- The charts have not been validated in ethnic minorities and in some may underestimate CVD risk. For example, in people originating from the Indian subcontinent it is safest to assume that the CVD risk is higher than predicted from the charts (1.4 times).

- An individual can be shown on the chart the direction in which his or her risk of CVD can be reduced by changing smoking status, blood pressure, or cholesterol, but it should be borne in mind that the estimate of risk is for a group of people with similar risk factors and that within that group there will be considerable variation in risk. It should also be pointed out in younger people that the estimated risk will generally not be reached before the age of 50, if their current blood pressure and lipid levels remain unchanged. The charts are primarily to assist in directing intervention to those who typically stand to benefit most.

(Continued over)

NONDIABETIC MEN

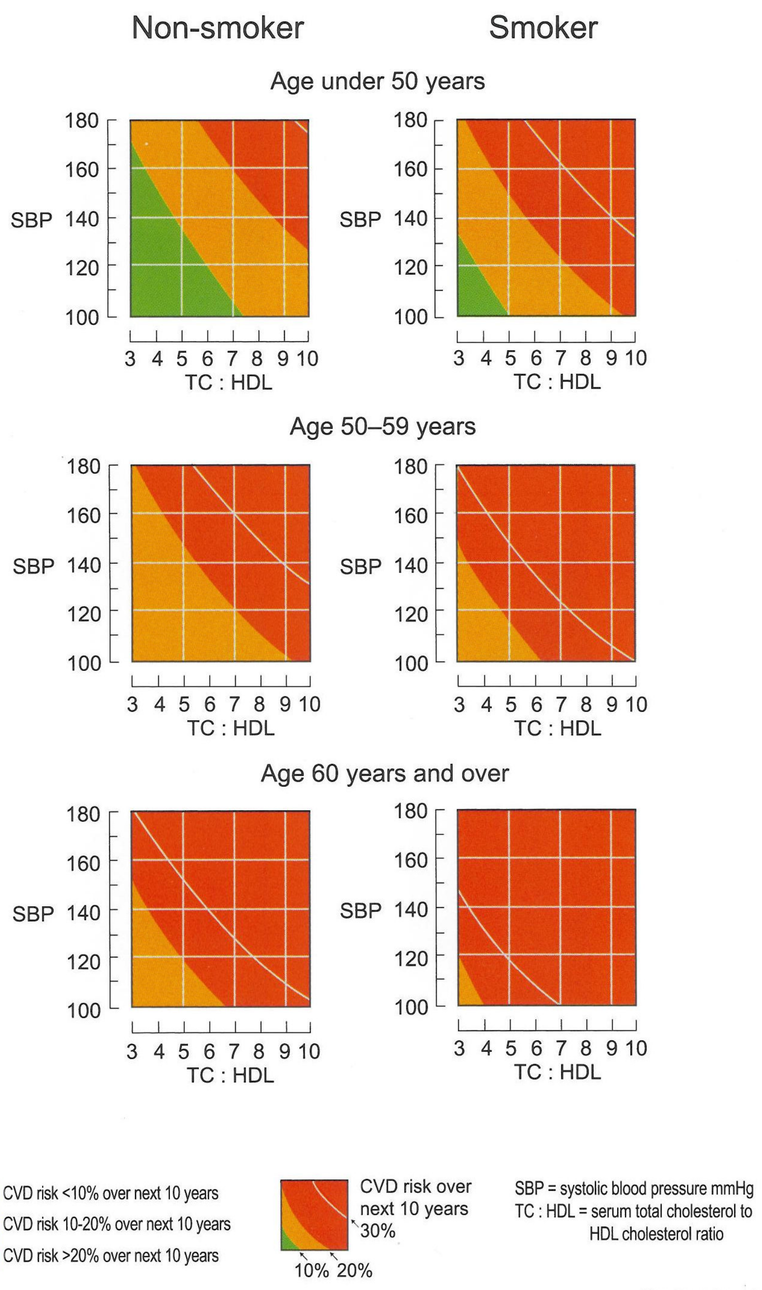

(Continued over)

NONDIABETIC WOMEN

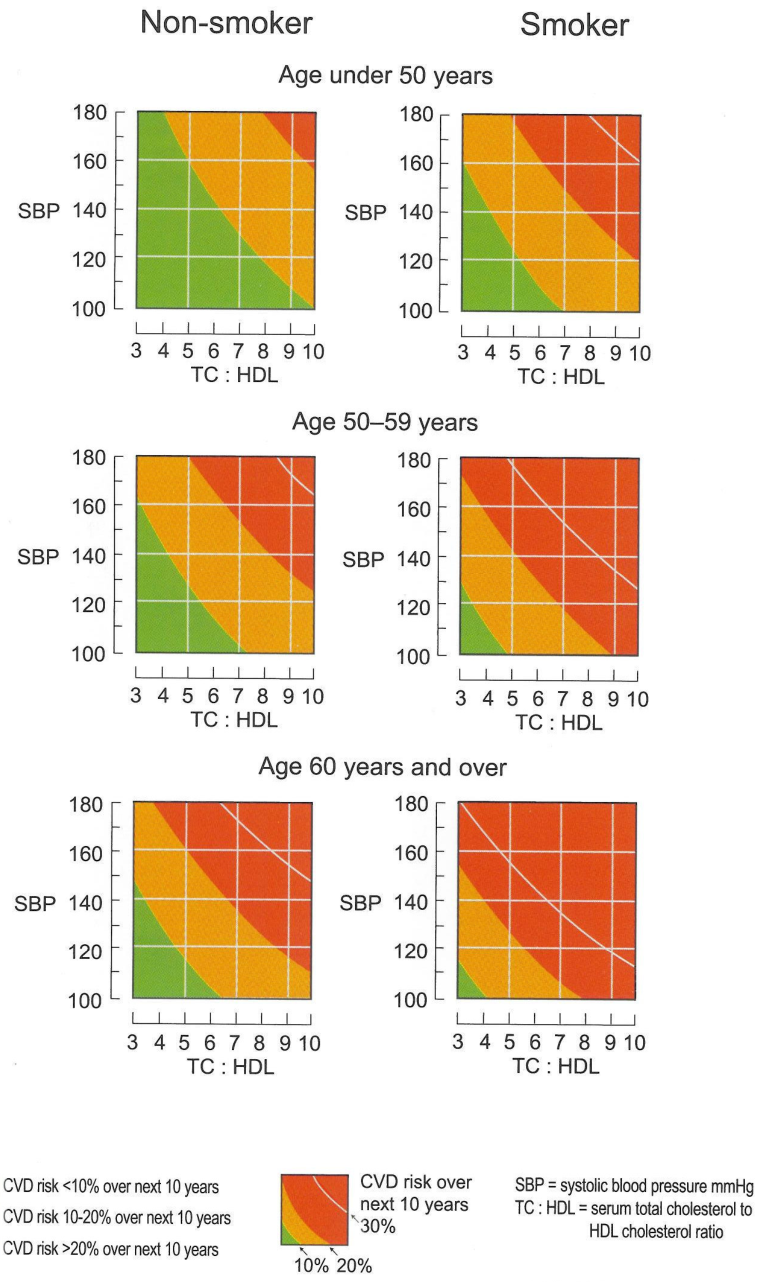

© The University of Manchester

APPENDIX 12: LABORATORY METHODS & SPECIMEN STORAGE

Storage specimens
8ml of EDTA blood to be taken for storage at screening and 4ml EDTA blood to be taken for storage at Week 4 (PI monotherapy arm only), Week 12 and then annually. An additional 8ml EDTA sample will be taken at any visit where a second VL test is performed as confirmation of viral rebound.

These samples will be collected in a 1x 4ml EDTA collection tube, processed within 4 hours from the time of blood draw. Any leftover blood will be separated, and the plasma stored in 1ml aliquots at -70°C in case future testing is required.

Leftover urine samples will be stored at the screening and annual study visits.

Stored blood and urine samples will be labelled with the patient's trial number and draw date. Samples must be processed, stored and documented until collection and centralisation of the samples at the central repository at Mill Hill. Shipping of specimens from sites to the central repository will be conducted annually.

Central resistance testing
In order to be able to extrapolate the findings beyond the 5-year period of the trial, it will be important to document that the patients on PI monotherapy who remain virologically suppressed during the trial have full virological suppression (to the same extent as patients on triple-therapy). Therefore, a single sample will be sent for testing by a very low copy assay (<5 copies/ml) at a central laboratory in all patients who have VL <50 copies/ml on conventional testing at the last follow-up visit of the trial.

Therapeutic Drug Monitoring
Patients in the PI monotherapy arm will have a blood sample taken at the Week 4 visit for measurement of the trough level of the PI. Samples in the UK will be sent for TDM processing via normal hospital procedures to Delphic Diagnostics. Results will be made available to the sites within approximately 2 weeks of sample collection.

Arrangements for TDM at non-UK sites are to be confirmed.

APPENDIX 13: NEUROCOGNITIVE ASSESSMENT TOOLS

Hopkins Verbal Learning test-revised (HVLT-R)

For this test the examiner will read aloud a list of 12 words and immediately after finishing that, the participant will be asked to freely recall them. The test is performed a further two times asking the participant to recall the words immediately after each reading. After the third exercise of free recall is completed, the examiner will read aloud a list of 24 words which includes the 12 words used for the previous part of the test. The participant will be asked to answer "yes" or "no" as the examiner reads each word if s/he recognises that word as one of the words included in the original 12 word list used for the first part of the test. The list of 24 words to be used in the second part of the test (recognition trial) includes, apart from the 12 "target words", 6 words that are categorically related to the target words. It also includes 6 unrelated words. After completing all the other neuropsychological tests and the MOS-HIV quality of life questionnaire (i.e. after about 15-20 minutes) the examiner will ask the participant to recall the words read at the beginning of the test (without the examiner repeating the list again) in order to assess delayed memory.

Each word recalled is scored during the immediate free recalling section of the test (range 0 – 36) and during the delayed recall test (range 0 – 12). In addition, the total number of correct responses is scored from the recognition section of the test. To control for the effect of practising on repeated administration a different version of the test, with different lists of words, will be used at each visit. A number of different versions of the test are commercially produced (Psychological Assessment Resources Inc).

Colour Trail Test: Part 1

Procedure

1) Position the participant at a table.
2) Place the practice sheet for part 1 flat on the table in front of them.
3) Provide the participant with a pen and explain the test to them showing the elements in the practice sheet. Ask the participant to draw a line between number 1 and number 2, then to draw a line between 2 and 3 and so on, in order, until reaching the end (number 8 in the practice sheet).
4) Ask the participant to not lift the pen from the paper.
5) Ask the participant to complete the practice test.
6) After successful completion of the practice test, show the participant the test sheet. Explain to the participant that on this page there are more numbers, but the procedure is the same.
7) Ask the participant to complete the test in the same way s/he completed the practice sheet. The participant must draw lines between numbers in numerical order starting from number 1 and finishing at number 25.
8) Ask the participant to draw the lines as fast as s/he can, but remind them not to lift the pen from the paper.
9) Start timing and continue timing the test even if the patient makes errors until s/he reaches the end (number 25).
10) If the participant makes an error, say "stop" and return the participant to his or her last correct response.
11) Stop timing when the participant reaches number 25 and record the time.

Colour Trail Test: Part 2

Procedure

1) Place the practice sheet for part 2 on the table in front of the participant.
2) Explain to the participant that this time they must draw a line from number 1 in "this" colour (pink), to number 2 in "this" colour (yellow) and continue to alternate the colours as they join successive numbers.
3) After successfully completing the practice sheet, show the test sheet to the participant.
4) Again, explain to the participant that in this sheet there are more number and letters. Ask the participant to complete the test in the same way s/he completed the practice one.
5) Ask the participant to draw the lines as fast as s/he can, but remind them not to lift the pen from the paper until the test is completed.
6) Start timing and continue timing the test even if the participant makes errors until s/he reaches the end (number 25). As in part 1, if the participant makes an error, return the participant to his or her last correct response.
7) Stop timing when the participant reaches letter L and record the time.

Grooved Pegboard test

The Grooved Pegboard (Lafayette Instruments) is a metal board (10 x 10 cm) with 25 holes arranged in 5 rows containing 5 holes each. Every hole on the board has a channel or groove randomly orientated in different directions. The kit also includes 25 metal round pegs with a key or ridge running longitudinally which must be placed in the holes. To do so, the participant needs to rotate the pegs to the correct position for insertion.

For the test, the examiner will ask the participant to insert all 25 pegs as fast as s/he can.

The participant must complete one row before starting a new one.

The test is timed, so the examiner will start timing when the participant is ready to begin the test and will stop timing only when the last peg is properly inserted. The test will be performed with each hand starting with the dominant one. Time to completion is scored separately for each hand.

Detailed instructions for all neurocognitive tests will be provided to the study sites and these will be followed closely for all assessments.

APPENDIX 14: PROTOCOL VERSION 2.0 LISTING OF CHANGES

Amendments to inclusion / exclusion criteria

Rationale: A number of changes to the criteria have been discussed at the Trial Steering Committee to facilitate recruitment to the trial as well as to maximise the generalisability and importance of the trial findings.

- **Page 20, section 4.1, Inclusion criterion 5**
  Version 1.1: "Plasma VL <50 copies/ml for at least 24 weeks prior to screening …"
  Version 2.0: "Plasma VL <50 copies/ml at screening and for at least 24 weeks prior to screening…."
  Reason: Original version would allow entry of a patient who had a detectable VL at screening which is inappropriate.

- **Page 20, section 4.2, Exclusion criteria 2 and 3**
  Version 1.1: Evidence of previous failure while taking a PI-containing regimen…. And Evidence of previous failure on an NNRTI-containing regimen….
  Version 2.0: Previous change in ART drug regimen for reasons of unsatisfactory virological response (patients who have changed regimen for prevention or management of toxicity or to improve regimen convenience are permitted to enter the trial).
  Reason: Patients who have failed treatment in the past will likely have NRTI drugs that are partially compromised by drug resistance, and hence there may be less to lose by dropping these drugs in the PI monotherapy strategy. However, the aim of this trial is to determine the place of PI monotherapy as an alternative to fully active first-line therapy.

- **Page 21, section 4.2, Exclusion criterion 11**
  Version 1.1: Past or current history of cardiovascular disease, or 10 year absolute coronary heart disease risk of >30% (calculated from the Framingham equation) and assessed using the Joint British Societies cardiovascular risk prediction charts.
  Version 2.0: Past or current history of cardiovascular disease, or 10 year absolute coronary heart disease risk of >30%, or risk of >20% if the patient has diabetes or a family history of premature ischaemic heart disease or stroke (based on the Framingham equation and assessed using the Joint British Societies cardiovascular risk prediction charts).
  Reason: The charts do not allow estimation of the impact of these additional risk factors, and in such patients it is important to use a lower threshold for exclusion in patients with additional risk factors.

- **Page 21, section 4.2, Exclusion criterion 13**
  Version 1.1: Patient currently receiving interferon therapy for Hepatitis C virus infection or considered likely to need such therapy during the course of the trial.
  Version 2.0: Patient currently receiving interferon therapy for Hepatitis C virus infection or planning to start treatment for Hepatitis C at the time of study entry.
  Reason: Predicting future Hepatitis C treatment requirements is unrealistic in this long-term trial.

- **Page 21, section 4.2, Exclusion criterion 14**
  Version 1.1: Co-infection with Hepatitis B, defined as Hepatitis BsAg positive at screening or at any time since HIV diagnosis

Version 2.0: Co-infection with Hepatitis B, defined as Hepatitis BsAg positive at screening or at any time since HIV diagnosis, unless the patient has had a documented Hepatitis B DNA measurement of less than 1000 copies/ml taken whilst off Hepatitis B active drugs.
Reason: Patients with low level hepatitis B viral replication are unlikely to be at risk of progressive hepatitis if NRTI drugs are discontinued.

- **Page 21, section 4.2, Exclusion criteria 15, 16, 17, 19 and 20**
  Version 1.1:
  15. "Any other active clinically significant condition, or findings during screening medical history or examination that would, in the opinion of the investigator, compromise the patient's safety or outcome in the trial"
  16. Anaemia…
  17. ALT ….
  19. Fasting plasma triglyceride level…
  20. Fasting cholesterol level
  Version 2.0: "Any other active clinically significant condition, or findings during screening medical history or examination, or abnormality on screening laboratory blood tests that would, in the opinion of the investigator, compromise the patient's safety or outcome in the trial".
  Reason: Many patients on HIV therapy have mildly deranged laboratory tests some of which might improve on switching to PI monotherapy. These criteria are too prescriptive and the decision on suitability of the patient is best left to the discretion of the treating physician.

- **Page 9, section 1.1.2, Summary**
  Version 1.1: Patients who have previously failed on a PI-containing regimen…
  Version 2.0: Patients who have had virological failure on any previous regimen…
  Reason: to reflect change in exclusion criteria.

Amendments to schedule of study visits and assessments

Rationale: Ongoing discussions with investigators have highlighted a few additional urine tests that could be performed for little effort and provide potentially useful information, as well as a few tests that can be done less frequently or omitted. Amendments have also been made to clarify sections of the protocol where the management strategy was not well described.

- **Page 11, Trial schedule table**
  Added to Version 2.0: This table has been updated, some rows have been combined or rearranged for clarity.
  Reason: to reflect the changes in assessments described below.

- **Page 22, section 4.3.2 Screening visit, paragraph 1**
  Added to Version 2.0: In the event that the patient has not fasted at the screening visit, the glucose and cholesterol measurement will be omitted and they will be asked to return within 1 week in the fasted state for these tests to be performed.

- **Page 22, section 4.3.2, Screening visit, paragraph 3:**
  Version 1.1: "charts based on the Framingham equation"
  Version 2.0: "the Joint British Societies cardiovascular risk prediction charts.
  Reason: It is important to standardise the assessment to a specified chart.

- **Page 22, section 4.3.2, Screening visit, paragraph 3:**
  Added to Version 2.0: Urine albumin / creatinine ratio and urinary phosphate tests, and urine storage.
  Reason: Decreased renal toxicity may be one advantage of PI monotherapy and it may be helpful (and is no extra burden to the patients) to perform these additional standard tests on the urine samples already collected at screening and annual visits. For the same reason it makes sense to store a urine sample at baseline, at annual visits and at study end in case further tests to explore any renal toxicity differences between the treatment groups might be of scientific interest. Consent will be obtained for urine storage.

- **Page 23, section 5.1, paragraph 3**
  Deleted from Version 2.0: "review of healthcare resource utilisation"
  Reason: Data will not be informative for the health economics analysis.

- **Page 25, section 6.2**
  Reason for changes in this section: It is important that patients in the triple therapy arm be managed according to a standard of care approach that is as similar as possible to the PI monotherapy arm. This was not well described in version 1.1 of the protocol. This section has therefore been expanded to describe more thoroughly the standard of care applicable in the control arm. The changes are as follows:

  Paragraph 1
  Added to Version 2.0: heading "6.2.1 Choice of triple therapy drugs".
  Added to Version 2.0: The sentence "They will have regular viral load ……" has been moved to the following section.

  Paragraph 2
  Added to Version 2.0: "In the event that the patient develops drug resistance to all three standard classes of drugs, the clinician may select any combination of drugs that is considered clinically appropriate and this need not be limited to standard-of-care triple therapy.
  Any change in therapy should be followed by a viral load at 4 weeks to verify that undetectable viral load is maintained."

  Section 6.2.2
  Added to Version 2.0: Section 6.2.2 Virological monitoring and switching strategy
  Patients in the triple therapy arm will have VL testing performed every 12 weeks during the study (see trial schedule, section 1.3). In the event of VL rebound greater than 50 copies/ml (confirmed on a second laboratory run, as described in section 7.2.1), the patient will be contacted by telephone, counselled on medication adherence, and asked to return for a repeat blood test within 4-6 weeks from the date of the first test. At this next visit, blood will be drawn for a repeat VL test, for resistance testing (saved and performed only if VL rebound confirmed) and for PIVOT blood storage (2 X 4ml plasma samples). The assessments performed at this visit are described in section 7.1.5.
  Patients who develop confirmed virological rebound (defined as 2 consecutive VL values above 50 copies/ml taken 4-6 weeks apart, with the first being confirmed by a repeat assay on the first sample) whilst taking triple therapy will ordinarily be expected to change therapy to a new optimised regimen, taking into account the results any resistance tests performed. However the clinician and /or patient may elect to continue the current regimen without change if considered appropriate, and the reasons for not changing therapy will be documented on the case report form. A further VL must be obtained within 4 weeks (together with an additional 2X 4ml sample for plasma storage) and if this remains detectable patients should change therapy.

If the clinician and/or patient choose to continue the triple therapy regimen, the reasons for not changing therapy will again be documented on the case report form. VL testing will be repeated at least as often as every 12 weeks (more often if clinically warranted). A blood sample for resistance testing and PIVOT plasma sample storage (1 X 4ml) will also be repeated every 12 weeks until VL suppression occurs spontaneously or treatment is changed. At the time of changing therapy a repeat sample will be sent for resistance testing and a PIVOT plasma sample will be taken (1 X 4ml) if these have not been performed in the 4-weeks prior to switching therapy.

- **Page 26, section 6.3.1, paragraph 3**
  Added to Version 2.0: "Patients will be reminded of the importance of maintaining excellent adherence when they switch to PI monotherapy. They will be instructed that, in the event of experiencing new side effects or for other reasons, they should not interrupt or change medication themselves but rather should seek medical advice from the study team so that changes or interruption in medication can be managed appropriately".

- **Page 26, section 6.3.2, paragraph 3**
  Added to Version 2.0: "The PI drug concentration level will also be measured to ensure that therapeutic drug levels are attained with the new PI".

- **Page 27, section 6.3.4**
  Reason for changes in this section: This section has been modified slightly to increase clarity and to achieve consistency with the approach in the triple therapy arm.

  Paragraph 1
  Version 2.0: First paragraph replaced with "Patients in the PI monotherapy strategy arm will have VL testing performed at Weeks 4, 8 and 12, and every 12 weeks thereafter (see trial schedule, section 1.3). In the event of VL rebound greater than 50 copies/ml (confirmed on a second laboratory run, as described in section 7.2.1), the patient will be contacted by telephone, counselled on medication adherence, and asked to return for a repeat blood test within 4-6 weeks from the date of the first test. At this next visit, blood will be drawn for a repeat VL test, for resistance testing (saved and performed only if VL rebound confirmed) and for PIVOT blood storage (2 X 4ml plasma samples). The assessments performed at this visit are described in section 7.1.5.

  Paragraph 2
  Version 1.1: "will switch promptly back"
  Version 2.0: "should switch promptly back"
  Added to Version 2.0: "The reasons will be documented on the case report form."
  Version 2.0: last sentence replaced with "A further VL must be obtained within 4 weeks (together with an additional 2 X 4ml plasma sample for storage). If the VL remains detectable, patients should switch back to triple-therapy immediately."
  Reason: some investigators have indicated that they would wish to have some leeway to continue PI monotherapy if the level of detectable VL is very low.

  Paragraph 3
  Added to Version 2.0: If the clinician and /or patient choose to continue the PI monotherapy regimen, the reasons for not restarting triple therapy will again be documented on the case report form. This should also be discussed with the chief investigator at MRC CTU. If the PI monotherapy is maintained, VL testing will be repeated at least as often as every 12 weeks (more often if clinically warranted). A blood sample for resistance testing and PIVOT plasma sample storage (1 X 4ml) will be repeated every 12 weeks until VL suppression occurs spontaneously or treatment is changed. At the time of changing therapy a repeat sample will be sent for resistance

testing and a PIVOT plasma sample will be taken (1 X 4ml) if these have not been performed in the 4-weeks prior to switching therapy.

Paragraph 4:
Added to Version 2.0: When a patient resumes triple therapy they will be managed exactly as for patients in the control arm, as described in section 6.2.

- **Page 28, section 6.5, paragraph 4**
  Version 1.1: 10-hour fast
  Version 2.0: minimum 8-hour fast
  Reason: consistency with other parts of protocol

- **Page 29, section 6.7, paragraph 1**
  Version 1.1: 3-month supply
  Version 2.0: One to three month supply

- **Page 30, section 6.8, title**
  Version 1.1: "measures of adherence"
  Version 2.0: "Adherence assessment and counselling"

  Paragraph 1
  Version 2.0: paragraph replaced with "At each of the trial visits, patients will be asked several simple questions to assess their adherence (number of missed doses since the last trial visit, number of missed doses in previous 2 weeks). The answers will be captured on the visit CRF, and patients will be given counselling to reinforce the critical importance of adherence if their responses indicate that adherence is not optimal.

- **Page 31, section 7.1.1, List of tests**
  Added to Version 2.0: Review of healthcare resource utilisation since last visit (patient self-report and review of case sheet); EQ-5D health status questionnaire.
  Reason: It is important to assess the health economic parameters at all study visits.

- **Page 32, section 7.1.2, List of tests**
  Added to Version 2.0: Review of healthcare resource utilisation since last visit (patient self-report and review of case sheet); EQ-5D health status questionnaire.
  Reason: It is important to assess the health economic parameters at all study visits.

- **Page 32, section 7.1.3, List of tests - Bone profile**
  Deleted from Version 2.0: "only required if patients taking tenofovir"

- **Page 32, section 7.1.3, List of tests - Urine protein/creatinine ratio**
  Deleted from Version 2.0: "only required if patients taking tenofovir"

- **Page 33, section 7.1.3, Subsequent visits**
  Version 2.0: renumbered 7.1.4

- **Page 33, section 7.1.4 and section 7.1.5**
  Version 1.1: The visit windows are ± 4 weeks….
  Version 2.0: The visit windows are ± 6 weeks

- **Page 33, section 7.1.5, List of tests**
  Version 1.1: section 7.1.4
  Version 2.0: renumbered section 7.1.5

Version 2.0: First bullet changed to "Review of symptoms (including specific questions about neuropathy and body fat changes)".
Added to Version 2.0: Urine albumin / creatinine ratio and phosphate, and urine storage.

- **Page 34, section 7.1.5, Additional visits for virological rebound**
  Reason: The management of patients who have viral load rebound is critical in this trial. In the previous version of the protocol, the assessments needed at these visits were not well described. This section has therefore been expanded to more fully describe the visits and assessments in patients who have VL rebound.

Added to Version 2.0: ***Second VL rebound visit***
For this repeat visit, patients in the PI monotherapy arm should be instructed not to take their morning dose of PI medication (other medications can be taken) until after the blood has been drawn. All other patients can take their morning medication as scheduled. The following assessments will be performed at this visit:

- Review of symptoms
- Review of concomitant medications
- Adherence to ART (no. of missed doses in previous 2 weeks and 3 months)
- Targeted physical examination (as needed to evaluate reported symptoms)
- Review of healthcare resource utilisation since last visit (patient self-report and review of case sheet)
- EQ-5D health status questionnaire (self-completed by patient)
- Laboratory tests:
  - HIV viral load
  - Resistance testing (store until VL test result available and only proceed if >50 copies/ml on the second sample)
  - Plasma storage (2x 4ml EDTA sample for later batched tests which may include PI drug levels)
  - Additional tests as per trial schedule or if clinical indication

If the second VL test shows an undetectable VL, follow-up will resume according to the next routine visit in the trial schedule. If the second VL test shows a VL >50 copies/ml, patients will be managed as described in section 6.2.2 and 6.3.4. Ordinarily, therapy will be changed and patients will subsequently be managed as described in section 7.1.7 below. If therapy is continued unchanged, then a third VL rebound visit will be arranged to occur within 4 weeks.

Third VL rebound visit
The same assessments listed above (for the 2nd rebound visit) will be performed.

If the third test shows undetectable VL, follow-up will resume according to the next routine visit in the trial schedule. If the third VL test shows a VL >50 copies/ml, patients will be managed as described in section 6.2.2 and 6.3.4. Ordinarily, therapy will be changed and patients will subsequently be managed as described in section 7.1.7 below. If therapy is continued unchanged, then subsequent visits will be arranged at no less often than every 12 weeks until the patient changes treatment.

Subsequent VL rebound visits
At these subsequent visits the following assessments will be performed:

- Review of symptoms
- Review of concomitant medications
- Adherence to ART (no. of missed doses in previous 2 weeks and 3 months)
- Targeted physical examination (as needed to evaluate reported symptoms)

- Review of healthcare resource utilisation since last visit (patient self-report and review of case sheet)
- EQ-5D health status questionnaire (self-completed by patient)
- Laboratory tests:
    - HIV viral load
    - Resistance testing (needed if more than 70 days have elapsed since the date of the last resistance test sample; store blood and only proceed if VL > 50 copies/ml on this sample)
    - Plasma storage (1 x 4ml EDTA sample for later batched tests which may include PI drug levels)
    - Additional tests as per trial schedule or if clinical indication

When the patient eventually changes treatment, an additional visit will be arranged as described in section 7.1.7 below.

- **Page 37, section 7.2.5, paragraph 3**
  Version 1.1: Trail making tests (two parts)
  Version 2.0: Colour trails tests (two parts)
  Added to Version 2.0: In Part 1, participants will be asked to connect a series of encircled numbers in numerical order. In part 2 they will be asked to connect a series of 25 encircled numbers alternating between two colours"

- **Page 37, section 7.2.5, paragraph 5**
  Version 1.1: "a table in which raw scores are categorised as average, above average and well above average based on standard population data…."
  Version 2.0: "a table of normal ranges for each of the tests based on standard population data…"

- **Page 37, section 7.3, paragraph 2**
  Added to Version 2.0: "and the final study visit"

- **Page 37, section 7.3, paragraph 5**
  Version 1.1: "Patients will be asked about any loss of fat in the face, and asked to say whether they consider…."
  Version 2.0: Patients will be asked about any change in fat in the face, arms, legs or abdomen. Taking into account the patient's opinion, the research nurse or doctor will grade facial lipoatrophy as none, moderate (evident on inspection), or severe (immediately obvious).

- **Page 37, section 7.3, paragraph 6**
  Added to Version 2.0: Patients will be asked about symptoms of peripheral neuropathy at baseline, annual visits and the final study visit. A brief neuropathy examination will be performed at baseline and the final study visit in all patients, and at intervening annual study visits if the patient reports neuropathy symptoms.

- **Page 37, section 7.3, paragraph 7**
  Version 1.1: "charts based on the Framingham equation"
  Version 2.0: "the Joint British Societies cardiovascular risk prediction charts"

- **Page 38, section 7.3.1, paragraph 1**
  Deleted from Version 2.0: "(and possibly repeated at week 8 or 12)"
  Added to Version 2.0: If the PI concentration is low at week 4, then the test may be repeated at week 8 and/or week 12, at the clinician's discretion. A blood sample for PI concentration will also be taken at 4 weeks after any change in PI drug in patients on PI

monotherapy to ensure that adequate concentrations are attained with the new drug. If the levels are low, this may also be repeated at 8 weeks or 12 weeks following the introduction of the new PI (at the clinician's discretion).

- **Page 38, section 7.4**
  Version 1.1: "starting 3 months prior to randomisation and continuing for the duration of follow up"
  Version 2.0: "throughout the trial"

  Version 1.1: "visits to HIV clinics or GPs"
  Version 2.0: "visits to HIV clinics, outpatient (non-HIV) clinics, A&E departments or to GPs"
  Deleted from Version 2.0: "or during the 3 months prior to randomisation"

- **Page 41, section 9.2.2, Virological rebound**
  Version 2.0: paragraph replaced with "Defined as two consecutive tests, taken at least 4 weeks apart, with VL more than 50 copies/ml (the first test must also be confirmed by re-testing the same blood sample). There are a number of alternative definitions for virological rebound, each of which has advantages and limitations (28). The alternative definitions to be used in this trial will be described in the full statistical analysis plan".

- **Page 43, section 9.4**
  Version 1.1: "The IDMC will meet around the time…and at yearly intervals thereafter"
  Version 2.0: "The IDMC will meet once before the start of the trial, once within one year of start of recruitment, and at yearly intervals thereafter. In addition to the scheduled meetings, an IDMC meeting may be requested by IDMC members, the Chief Investigator or the Trial Steering Committee at any time"
  Reason: This reflects a decision made by the IDMC at their first meeting.

- **Page 46, section 11.2.1 (c)**
  Added to Version 2.0: "…. and is based on the information listed in the package insert or summary of product characteristics (available online at http://www.medicines.org.uk)."
  Reason: to provide clearer guidance on how to determine if an event is a SUSAR.

Changes to the PIVOT patient information sheet

Rationale: A few changes are required to reflect the changes in the study assessments, in particular the incorporation of urine storage.

- **Page 61**
  Version 1.1: Version 1.1, 25 March 2008
  Version 2.0: Version 2.0, 18 November 2008

- **Page 63, Study assessments and follow-up, paragraph 5**
  Added to Version 2.0: You will also need to provide a urine sample at screening and at annual study visits for routine tests, and a small amount of this (approximately 5ml, 1 teaspoon) will be stored in case of the need for future tests.

- **Page 63, Study assessments and follow-up, paragraph 6**
  Added to Version 2.0: Two additional tubes of blood will be collected for storage (8ml or 2 teaspoons) in case of the need for future tests.

- **Page 67**
  Version 1.1: Version 1.1, 25 March 2008
  Version 2.0: Version 2.0, 18 November 2008

- **Page 68, section 6**
  Added to Version 2.0: "and urine" samples to first sentence
  Added to Version 2.0: "any leftover blood and urine and these extra blood samples…"

- **Page 70**
  Version 1.1: Version 1.1, 25 March 2008
  Version 2.0: Version 2.0, 18 November 2008
  Added to Version 2.0: Section 6: insert "or urine"

Changes to sites and to study personnel

- **Page 3**
  Added to Version 2.0: Trial Physician
  Dr Alejandro Arenas-Pinto
  MRC Clinical Trials Unit
  222 Euston Road
  Email: AAP@ctu.mrc.ac.uk
  Tel: 0207 670 4716
  Fax: 0207 670 4817

- **Page 4, External Principal Investigator**
  Version 1.1: patricia.sanderson@chelewst.nhs.uk
  Version 2.0: Yvonne.Stupple@chelwest.nhs.uk

- **Page 4, Trial Management**
  Version 1.1: fvh@ctu.mrc.ac.uk
  Version 2.0: pivot@ctu.mrc.ac.uk

  Version 1.1: Data manager
  Version 2.0: Ellen Dyer, Tel: 0207 670 4926

- **Page 74, Appendix 4**
  Added to Version 2.0:
  Dr Iain Reeves
  Homerton University Hospital
  Homerton Row
  London, E9 6SR

  Added to Version 2.0:
  Prof Charles Lacey
  Hull York Medical School
  University of York
  York, YO10 5DD

  Added to Version 2.0:
  Dr Jane Minton
  St James's University Hospital
  Beckett Street
  Leeds, LS9 7TF

Deleted from Version 2.0: Two sites in Italy.
Reason: It is unlikely that they will participate.

Minor edits to appendices

- **Appendix 5:**
  Deleted from Version 2.0: page 2 of EQ-5D questionnaire
  Reason: No longer needed in this study.

- **Appendix 7:**
  Version 1.1: DAIDS table for grading severity of adult adverse experiences (1992).
  Version 2.0: DAIDS table for grading the severity of adult and pediatric adverse events (2004).
  Reason: We have updated the toxicity grading table to 2004 version.

- **Appendix 12, paragraph 1**
  Version 1.1: 4ml of EDTA blood to be taken for storage at baseline, Week 4….
  Version 2.0: 8ml EDTA blood to be taken for storage at screening, and 4ml EDTA blood to be taken for storage at week 4 …..

  Version 1.1: An additional sample……..
  Version 2.0: An additional 8ml EDTA sample …..

- **Appendix 12, paragraph 3**
  Added to Version 2.0: "Leftover urine samples will be stored at the screening and annual study visits"

- **Appendix 12, paragraph 4**
  Version 1.1: Stored blood samples….
  Version 2.0: Stored blood and urine samples….

- **Appendix 13**
  Version 1.1: "trail-making test"
  Version 2.0: "colour trails test"
  Reason: The colour trails test is now used in place of the trail making test, because the latter was not available from a reliable manufacturer and the text has been revised accordingly. The pictures previously in this section have been removed as all the sites are now familiar with the materials.
